# Influence of Rare Earth Elements on Prebiotic Reaction Networks Resembling the Biologically Relevant Krebs Cycle

**DOI:** 10.1002/anie.202516853

**Published:** 2025-11-26

**Authors:** Jonathan Gutenthaler‐Tietze, Carolina G. Heßler, Lena J. Daumann

**Affiliations:** ^1^ Chair of Bioinorganic Chemistry Heinrich‐Heine‐Universität Düsseldorf 40225 Düsseldorf Germany

**Keywords:** Krebs cycle, Lanthanides, Origins, Prebiotic reaction networks, Rare earth elements

## Abstract

Rare earth elements (REEs) are not rare, but rather abundant in the earth's crust and excellent catalysts for a multitude of organic reactions. They have been recently shown to be used in the active sites of bacterial enzymes and thus essential for metabolic processes. However, these elements have so far been disregarded with respect to their possible contributions to the emergence of complex molecules. Here, we investigate the potential of REEs to act as mediators in a prebiotic reaction network resembling the biological Krebs cycle starting from glyoxylate and pyruvate. Special focus is put on a comparison between trivalent REEs and ferrous iron. Reaction products were analyzed by gas chromatography–mass spectrometry (GC–MS) and nuclear magnetic resonance (NMR) spectroscopy. Contrary to Fe^2+^, the formation of the reduced starting materials seems to be a major pathway when REEs are involved. Their high coordination numbers, flexible coordination spheres and their hard Lewis acidic properties make REEs excellent reagents in abiotic chemical reaction networks resembling conserved biochemical pathways.

## Graphical abstract

Rare earth elements can act as mediators in a prebiotic reaction network, producing various intermediates of the citric acid cycle starting from glyoxylate and pyruvate. The ionic radius and the resulting variation in Lewis acidity has a significant influence on the observed product scope.

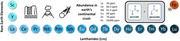

## Introduction

A main goal of Origin of Life (OoL) research is to narrow the gaps between prebiotic chemical reactions on the early Earth and modern biochemistry. To this end, it is necessary to get a complete picture of prebiotically plausible reactions covering all possible environments and conditions. However, one class of elements has so far been underrepresented with regards to OoL research—the rare earth elements (REEs) consisting of the lanthanides (Lns), scandium, and yttrium. These elements are, contrary to what their name might suggest, omnipresent in Earth's continental crust today with abundances comparable to other common metals, such as copper and zinc (La: 39 ppm, Ce: 66.5 ppm, Cu: 60 ppm, Zn: 70 ppm).^[^ ^1^ ^]^ In a prebiotic context, REEs are often used to investigate the nature and formation processes of the oldest terrestrial rocks^[^ ^2^, ^3^ ^]^ and are in part represented in minerals most likely to be found on Hadean Earth.^[^ ^4^, ^5^ ^]^ Thorium and uranium‐containing lanthanide minerals have also been discussed as a possible energy source for prebiotic chemistry in a natural nuclear reactor scenario.^[^ ^6^ ^]^ Additionally, REEs can be found in carbonaceous chondrite meteors.^[^ ^7^, ^8^ ^]^ From a reactivity stand point, their weak ligand field stabilization^[^ ^9^ ^]^ and subsequent high water exchange rate coupled with a low hydrolysis constant makes them efficient Lewis acid catalysts in water.^[^ ^10^, ^11^ ^]^ This fact alone makes it even more surprising that REEs have so far been neglected in OoL research. In contrast, the transition metal iron plays an integral part in the research into the abiotic generation of biological building blocks,^[^ ^12^ ^]^ given its important role in many different biological processes.^[^ ^13^ ^]^ Members of the REEs are the most recent addition to the group of biologically relevant metals.^[^ ^14^ ^]^ Methylotrophic bacteria can use lanthanides instead of calcium in the active sites of methanol dehydrogenase enzymes (MDH), an essential component in their C1 metabolism.^[^ ^15^, ^16^, ^17^, ^18^, ^19^ ^]^ The gene *xoxF* encodes for this Ln‐dependent MDH and as Keltjens et al. conclude, XoxF represents an evolutionary older prototype of the calcium‐dependent MDH (MxaF‐type).^[^ ^20^ ^]^ This fact, in combination with the likely existence of many more Ln‐dependent enzymes, hints toward an essential role of rare earth metals in life from its very beginning on.

OoL research is often inspired by biological processes. One example for this is the Krebs cycle, also known as tricarboxylic acid (TCA) cycle, which is at the center of the cellular metabolism of organisms relying on respiration. Within an abiotic framework, complex reaction networks starting from the prebiotically plausible pyruvate^[^ ^21^, ^22^ ^]^ and glyoxylate^[^ ^23^, ^24^ ^]^ have been shown to produce intermediates of the TCA cycle.^[^ ^25^, ^26^ ^]^ Additionally, under a reducing atmosphere of hydrogen and in the presence of nickel and ammonia, the formation of amino acids can be observed.^[^ ^27^ ^]^ In the presence of Fe^2+^ a formation and subsequent breakdown of Krebs cycle intermediates can be observed.^[^ ^28^ ^]^

In this study, we investigate the potential of REEs as reagents in a prebiotic reaction network resembling the biological Krebs cycle starting from glyoxylate and pyruvate. A special focus is put on a comparison between trivalent REEs and ferrous iron.

## Results and Discussion

Based on the seminal work of Moran and coworkers,^[^ ^28^ ^]^ we initially focused on a direct comparison of different REE chloride salts and ferrous chloride to survey their ability to mediate reactions in a prebiotic reaction network resembling the metabolic Krebs cycle. To that end, glyoxylic acid and sodium pyruvate were reacted with the respective metal salts for 3 h in mildly acidic water at 70 °C in a sealed vessel purged with nitrogen (concentrations: glyoxylate (66 mm); pyruvate (33 mm); metal chloride (66 mm)) simulating the environment of a hydrothermal vent. After subsequent work‐up and derivatization according to a modified literature procedure,^[^ ^28^ ^]^ the reaction products were investigated by gas chromatography coupled with mass spectrometry (GC‐MS) and NMR spectroscopy.

**Figure 1:**
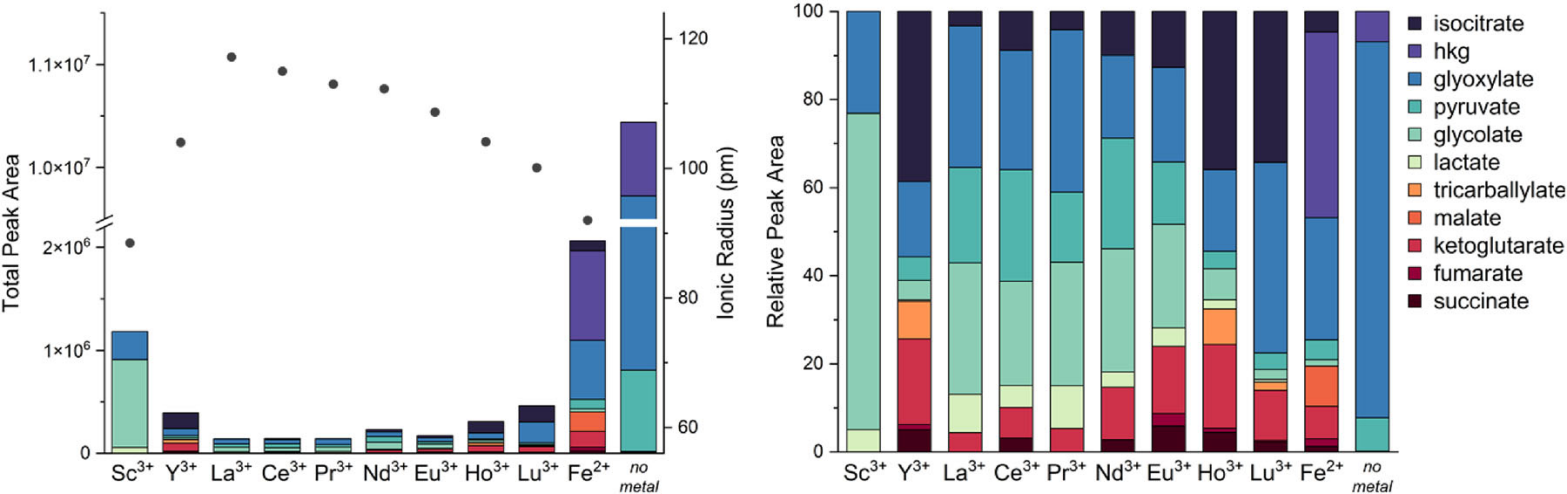
Absolute (plotted in combination with the respective ionic radii)^[^ ^29^ ^]^ and relative peak areas of reaction product gas chromatograms of glyoxlate and pyruvate in the presence of different REE chlorides, ferrous chloride, and no metal ions after 3 h at 70 °C (hkg = α‐hydroxyketoglutarate).

To avoid a harsh basification step to precipitate all present metal cations, which in itself could influence the product spectrum, we decided on an alternative, milder protocol using Chelex 100 resin (sodium form). Additional to the GC‐MS analysis (results shown in Figure 1, ^1^H NMR spectra were recorded for each reaction, and the pH was determined. The REEs that were chosen represent metal cations over the whole series, with different sizes and thus charge densities, possibly resulting in altered Lewis acidity and coordination numbers (Figure 1).

**Figure 2:**
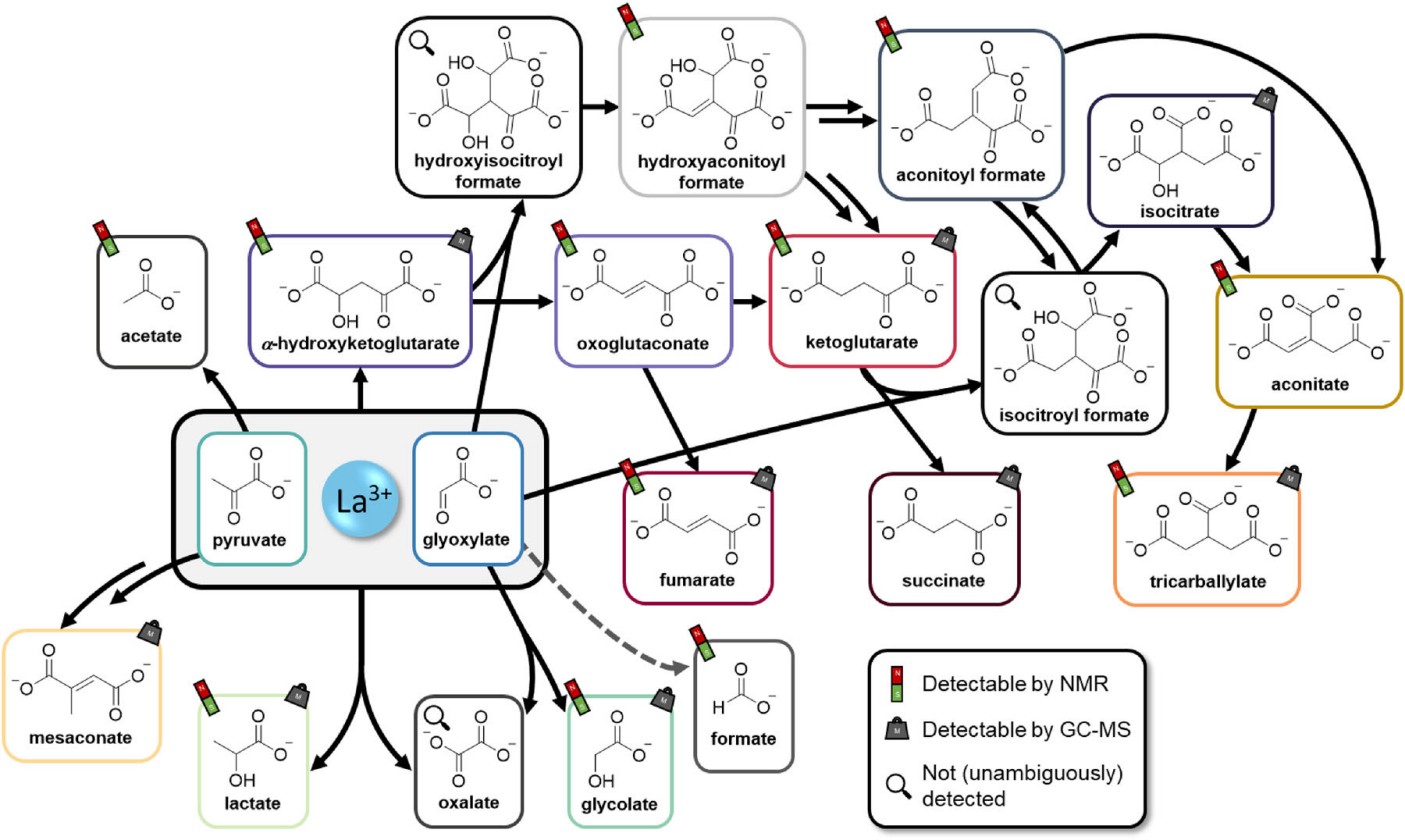
Reaction network arising from the La^3+^‐mediated reaction of glyoxylate and pyruvate, including non‐detected intermediates with the most probable routes represented by solid arrows requiring either one (direct arrow) or both of the reactants (arrow starting from grey box). The frame colors are consistent with respective substance coloring in the analytical data (see Figure 1 and Supporting Information).

In the REE‐containing reactions, with the exception of Sc^3+^, the formation of substantial amounts of precipitate can be observed (see Figure S40). Additionally, in the reactions containing the REE salts the pH of the reaction mixture drops during the reaction, whereas in the case of Fe^2+^ and the reaction containing no metal ions the pH rises. Nonetheless, very similar products were obtained using REEs or Fe^2+^. However, there is a readily observable difference in the relative product distributions between the trivalent REE chloride salts and FeCl_2_. The amount of dissolved products detected by NMR and GC‐MS is higher in the case of Fe^2+^ in comparison to the REE ions, disregarding Sc^3+^, which correlates with the aforementioned observation of precipitate formation. Within the REE series, reactivity seems to be primarily influenced by the ionic radius, where the smaller ions favor the products requiring multiple steps, such as *α*‐ketoglutarate and isocitrate. This point is further emphasized by the almost identical product distribution according to GC‐MS analysis of the reactions containing Y^3+^ and Ho^3+^, both of which have very similar ionic radii (Y^3+^: 104 pm; Ho^3+^: 104.1 pm).^[^ ^29^ ^]^ A key difference between the reactivity of Fe^2+^ and the REE ions is the formation of lactate, the product of a reduction of the starting material pyruvate. Also, the reduction of glyoxylate itself seems to be a major pathway in the REE‐containing reactions with higher amounts of glycolate formed in the reactions containing the larger ions, such as La^3+^. Again, Sc^3+^ is an outlier here as it has the smallest ionic radius of the rare earths but shows the highest amount of formed glycolate. These observations could be explained by the significantly lower pH of the reaction mixture, which leads to different reactivity and solubility of the formed species. Overall, the trends observed by GC‐MS and NMR are similar over the whole series (see Figure S41) in the case where products could be detected by both methods (Figure 2). When it comes to Eu^3+^ and Ho^3+^, it should be mentioned that even when Chelex is used, a small amount of these paramagnetic ions in solution leads to spectral broadening in the NMR spectra and interfering with the water suppression. This makes integration unreliable as signals cannot be easily assigned as it is the case with the other metal ions. The product spectrum of the reaction mixture without added metal salt shows an overall concentration of pyruvate‐derived products of 22.1 mm compared to an expected concentration of 33.3 mm. In the absence of observable precipitate, this can be explained with product adsorption during work up using the Chelex exchange resin, which has been described for a similar setup.^[^ ^30^ ^]^ The observed percentages of lost material during resin‐incubation lay between 20% and 27%. Considering that not all signals observed in the NMR spectrum could be assigned, these values align well with the 34% discrepancy observed here.

Next, we wanted to track the variation in the product spectrum over time. As metal salts FeSO_4_ and La_2_(SO_4_)_3_ were chosen, as preliminary experiments showed a higher reactivity for La_2_(SO_4_)_3_. This might be explained by the presence of the weakly basic sulfate anion. The stoichiometry was changed to 2:1:1 (glyoxylate:pyruvate:Fe^2+^/La^3+^). The reaction mixture was analyzed before metal salt addition (0 h) and after 1, 3, 5, 24, 48, and 72 h, respectively. The product spectrum at each point in time was analyzed via GC‐MS and ^1^H NMR spectroscopy. Additionally, the pH was determined.

Gas chromatograms of the reaction containing Fe^2+^ show no significant changes after 24 h (see Figure S34). The NMR spectra paint a similar picture with only slight changes after 24 h (see Figures S15 and S16). Especially in the NMR spectra after 1 h and 3 h the presence of paramagnetic iron in solution is yielding broad peaks making reliable integration impossible. This is an indication that the removal of metal ions is insufficient with the chosen protocol. After 5 h and beyond, the improved resolution of the spectra coincides with the observed formation of significant amounts of precipitate in the reaction vessel (see Figure S36). Overall, the formation of α‐ketoglutarate and tricarballylate as main products is verifiable by NMR and GC‐MS. In the low‐field region of the spectra, the formation of oxoglutaconate and formate can clearly be observed, both of which could not be detected by GC‐MS. The pH of the reaction mixture starts after metal salt addition at 4.7 and settles at about 6.5 after 24 h (see Figure S36).

**Figure 3:**
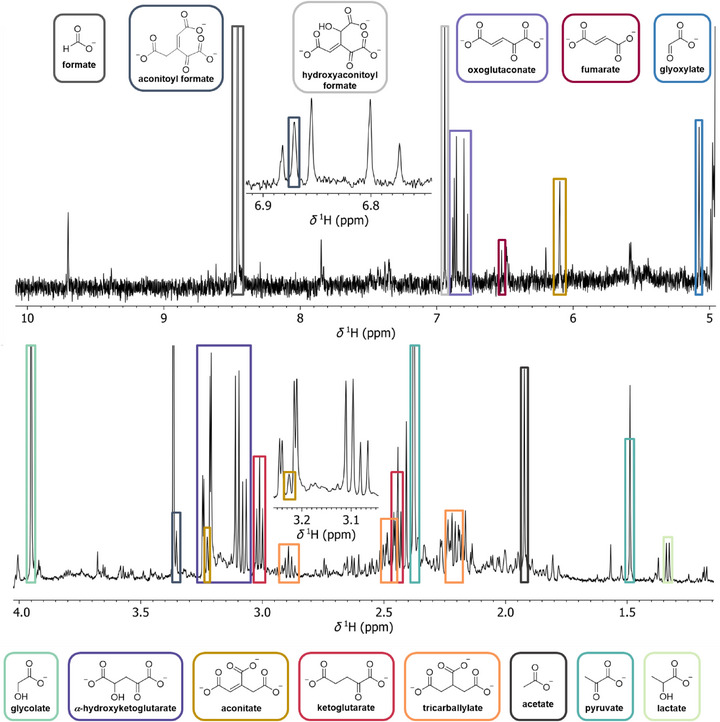
Representative ^1^H NMR spectrum of the 2:1:1 (glyoxylate:pyruvate:La^3+^) reaction mixture containing La_2_(SO_4_)_3_ after 72 h at 70 °C with the assignable signals marked in colored boxes and the respective structures marked with the same color.

Similar to the reaction containing Fe^2+^, in the reaction involving La_2_(SO_4_)_3_ the product spectrum and distribution stays relatively constant after 24 h (see Figure S33) according to GC‐MS. However, just as in the reactions involving the REE chlorides and contrary to Fe^2+^, the formation of the reduced starting materials seems to be a major pathway which can also be confirmed by NMR spectroscopy (see Figures 3 and S13). While the quantity of lactate is comparatively stable over time, the quantity of glycolate is increasing continuously. Another distinction, just as in the aforementioned 3 h reactions involving various metal chlorides, is the initial drop in pH. While the La^3+^ reaction starts off at a pH of about 5.7, after 3 h the pH of the reaction mixture reaches a minimum of 5.1 and subsequently reaches 5.4 after 72 h.

At the start of the reaction, La_2_(SO_4_)_3_ never fully dissolves, and a small amount of colorless precipitate is observable in the reaction vessel (see Figure S35). Already after 3 h a substantial increase in the amount of precipitate can be detected. The precipitate is still present after the treatment with the Chelex resin. This prompted us to investigate the lanthanum concentration in solution at this point. Inductively coupled plasma‐optical emission spectroscopy (ICP‐OES) gave a value of 330 µg∙mL^−1^, which corresponds to 2.4% of the initially added amount of La^3+^. An elemental analysis of the precipitate after 3 h revealed a very low sulfur content in combination with a relatively low hydrogen content (C: 15.49, H: 2.56, S: 1.01). The precipitate after 3 h with one equivalent of La^3+^ was also investigated by IR spectroscopy (see Figure S44). As a classical Cannizzaro reaction of glyoxylate yields oxalate as a product and as metal oxalates are usually poorly soluble, this might point to the possible nature of the precipitate. The IR spectra indicate the presence of an absorption band of the precipitate at 1313 cm^−1^, which would correspond to the C═O stretch of oxalate.^[^ ^31^ ^]^ Thus, the resulting precipitate is most likely a mixture of La^3+^ with oxalate, molecules of higher carbon content, such as pyruvate, and small amounts of sulfate.

The fact that the reaction continues at very low concentrations of dissolved La^3+^ might indicate catalytic activity of La^3+^ species present in solution or in a heterogenous fashion and in turn prompted us to investigate the influence of La^3+^ concentration at the start of the reaction. To this end, the reaction was repeated with 0.5, 0.2, 0.1 and 0 equiv. of La^3+^ in the form of La_2_(SO_4_)_3_ and the reaction mixtures were investigated via ^1^H NMR after 3 h. The NMR spectra (see Figures S17 and S18) show an increase in reaction dynamics at higher La^3+^ concentration detectable by a more pronounced decrease in concentration of the starting materials. However, the overall product spectrum and concentrations in all reactions containing La^3+^ are highly similar. The reaction including the starting materials without La^3+^ does not show the formation of oxoglutaconate, ketoglutarate, or lactate. Additionally, to probe the activity of the precipitate, after 3 h the solid obtained from a previous run was added to the reaction mixture as sole La^3+^ source, and the reaction mixture was investigated after 72 h. For comparison, analogous reactions containing 0.1 equiv. of La^3+^ in the form of La_2_(SO_4_)_3_ and reactions containing no additional metal ions were performed. Whereas no lactate and only very small amounts of glycolate and ketoglutarate are formed in the metal‐free reaction according to the NMR spectra the formation of these products is clearly visible for either La‐containing reaction (see Figures S23 and S24). Additionally, the remaining amount of unreacted glyoxylate is much higher in the metal‐free reaction. Overall, these results may suggest catalytic activity of La^3+^ in this reaction network.

As the most notable difference between the REE‐containing reactions and those with Fe^2^⁺ is the formation of significant amounts of glycolate and lactate, the reduced forms of the two starting materials, these reactions justify a more detailed discussion. While Fe^2+^ could in principle act as a reducing agent,^[^ ^32^ ^]^ this is not the case for the used REEs. The reduction, however, could be explained by the intrinsic reactivity of aldehydes. A disproportionation of aldehydes is known as the Cannizzaro reaction^[^ ^33^ ^]^ and involves the hydride transfer of a reactive diol anion to the carbonyl function of a second aldehyde yielding the respective alcohol and carboxylate after a proton transfer.^[^ ^34^ ^]^ When a different carbonyl‐containing molecule is present as hydride acceptor the reaction is generally called crossed Cannizzaro reaction. These reactions usually occur under strongly basic conditions as the first step involves the nucleophilic attack of a hydroxide anion to the aldehyde. In the case of aldehydes with another carbonyl in the *α*‐position, a different mechanism comes into play when metal ions are involved. The coordination of the metal center by the 1,2‐dicarbonyl‐containing molecule allows for an intramolecular 1,2‐hydride shift.^[^ ^35^ ^]^ This reaction has been reported with different metal ions including the REEs scandium and ytterbium.^[^ ^36^ ^]^ With regards to OoL research, an aza‐Cannizzaro reaction has been shown to form glycine from glyoxylate in ammonium‐rich water at neutral pH.^[^ ^37^ ^]^ In the case of lactate formation in the context of this work, a possible mechanism is shown in Scheme S2. In a first step, the glyoxylate hydrate loses a proton and forms the reactive diol anion. Subsequently, a hydride is transferred to the carbonyl of an adjacent pyruvate molecule, with a proton transfer yielding the products oxalate and lactate. The role of La^3+^ in this reaction could be the activation of the carbonyl function, as well as bringing both substrates in close proximity to one another. In the latter case, the more flexible coordination sphere of La^3+^ in comparison to transition metals might be advantageous.

**Figure 4:**
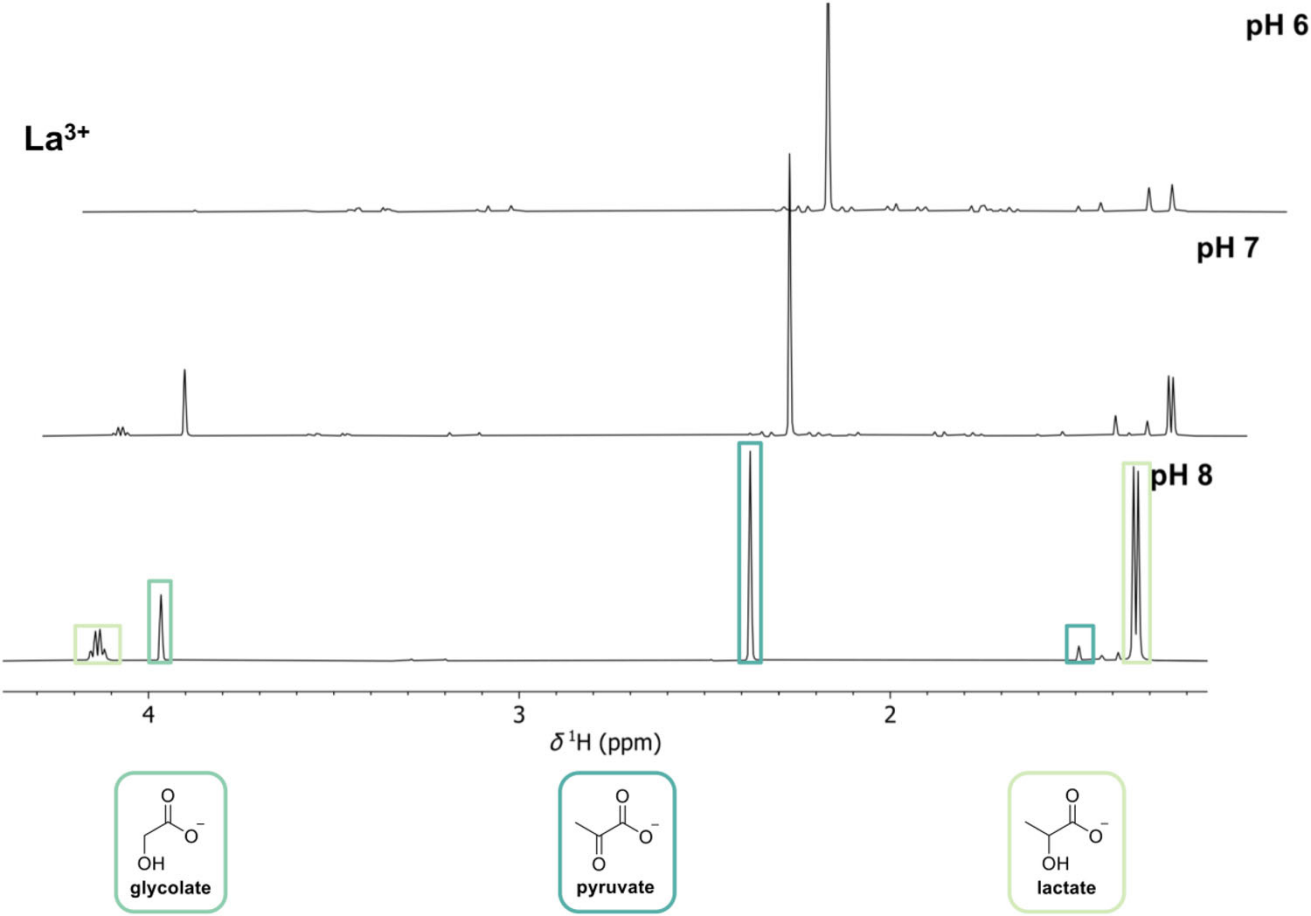
^1^H NMR spectra (higher field) of the reaction mixtures with different starting pH values with the assignable products marked in boxes and the corresponding structures marked with the same color below.

As the Cannizzaro reaction is highly dependent on the pH of the reaction medium, we tested the effect of different starting pH values. To suppress the reaction of glyoxylate with itself, the stoichiometry was changed to 1:1:0.5 (glyoxylate:pyruvate:La_2_(SO_4_)_3_) and the pH after metal salt addition was adjusted to 6, 7, and 8, respectively. NMR spectra of the reaction mixtures after 3 h paint a clear picture (see Figure 4). While the product spectrum of the reaction with a pH starting value of 6 shows almost no lactate and glycolate, the lactate signals become more pronounced at pH 7. At pH 8, the ratio of lactate to pyruvate is 1.9:1 with no other significant product peaks, apart from a small amount of glycolate (see Table 1 for yields of lactate and glycolate depending on pH). The IR spectra of the respective precipitates isolated after the reactions show pronounced similarities with lanthanum(III) oxalate at pH 7 and pH 8 (see Figure S43). At pH 6, the spectrum of the precipitate looks almost identical to the corresponding IR spectrum of the aforementioned 2:1 reaction after 3 h. Thus, it can be concluded that at pH 7 and 8 the reaction becomes much more selective towards the crossed Cannizzaro reaction with oxalate as a byproduct. At lower pH, aldol condensation reactions are more prominent.

**Table 1:** NMR yields of lactate and glycolate, as well as pyruvate conversion with respect to the adjusted starting pH of a 1:1:0.5 reaction (glyoxylate:pyruvate:La_2_(SO_4_)_3_) after 3 h at 70 °C.

pH	Pyruvate conversion	Lactate yield	Glycolate yield
6	80.0%	0.2%	0.3%
7	79.8%	8.8%	7.9%
8	85.1%	28.1%	7.7%

These observations also highlight the importance of controlling and testing pH for such reaction networks, also during the workup of samples. With the omission of the basification step during derivatization for GC‐MS analysis and additional analysis via NMR, we could suppress possible unwanted reactions and better observe molecules actually present in the reaction mixture. For example, *α*‐hydroxyketoglutarate is no longer detectable with either GC‐MS or via NMR after addition of base. The recommended amount of Chelex according to the manufacturer instructions was not sufficient in the case of stoichiometric use of Fe^2+^, Eu^3+^, and Ho^3+^ as indicated by line‐broadening in the ^1^H NMR. A 3‐fold increase in the used amount of exchange resin leads to sharp signals in the case of Fe^2+^ indicating complete removal of the paramagnetic metal ions (see Figures S21 and S22). On the other hand, for reactions involving La^3+^, one might assume that the negligible concentration of metal ions in solution, due to extensive precipitation, renders Chelex treatment unnecessary. However, a comparison of ^1^H NMR spectra of the supernatant and the resin‐treated resuspended reaction mixture after 72 h shows notable differences (see Figures S19 and S20). Both the observation of line sharpening for the diastereotropic methylene protons and the increase in signal intensity for tricarballylate suggest the presence of dynamic coordinated species in the untreated supernatant.

Beyond the Cannizzaro reaction, additional redox processes take place within the La^3+^‐mediated reaction network. The formation of fumarate and acetate most likely involves the oxidative decarboxylation of *α*‐keto acids. In the analogous iron‐mediated case, these pathways were previously explained by the presence of oxidative Fe^3+^, which is supposedly formed during the reduction of oxoglutaconate to ketoglutarate.^[^ ^28^ ^]^ In the metal‐free case, an additional oxidant, such as hydrogen peroxide, is needed for the decarboxylations.^[^ ^26^ ^]^ However, these reactions do not explain the observed reactivity in the presence of the redox inert La^3+^ ion. Instead, the decarboxylations could be accompanied by simultaneous La^3+^‐mediated autooxidation and hydride‐transfer reactions.

## Conclusion

The important role of metals in prebiotic reactions is undisputed.^[^ ^38^, ^39^ ^]^ However, the potential of REEs in this respect has so far been overlooked. In this work, we have shown that REEs have a substantially different reactivity compared to iron in the framework of a prebiotic reaction network based on the biological Krebs cycle starting from the prebiotically plausible glyoxylate and pyruvate. Especially, the formation of the reduced starting materials as a major reaction pathway at neutral pH might represent an intriguing niche for REEs in origins research. Also, the observation of La^3+^ reactivity in catalytic amounts may indicate the ability of REEs to catalyze these types of reactions in a prebiotic setting, especially where a flexible coordination sphere involving multiple reactants and strong Lewis acids are needed. Furthermore, the hard Lewis acid character is especially relevant in the context of carbonyl/oxygen‐donor ligand containing molecules. Although many reaction pathways shown here are hard‐wired into the starting materials,^[^ ^26^ ^]^ the abundance of REEs and their favorable properties put these elements in a new light as potent mediators in an OoL scenario. While this study focuses on the Lewis acidity of REEs in a prebiotically plausible scenario, a variety of other aspects remain to be addressed in future studies, such as the redox chemistry of cerium^[^ ^40^ ^]^ and photoredox catalysis.^[^ ^41^ ^]^

## Supporting Information

Additional information on experimental procedures, used methods and materials, as well as data supporting the findings in this manuscript can be found in the Supporting Information.

## Author Contributions

J.G.‐T. and L.J.D designed the study and wrote the initial draft of the manuscript. J.G.‐T. and C.G.H. performed the experiments and did the analytical work. L.J.D. provided the necessary infrastructure. All authors were involved in the review of the manuscript.

## Conflict of Interests

The authors declare no conflict of interest.

## Supporting information



Supporting Information

## Data Availability

All NMR, GC‐MS and IR raw data have been deposited at RADAR4Chem with the following DOI: 10.22000/mbt1j4jx33wj0tkj.
