# Supplementary material for: Influence of Rare Earth Elements on Prebiotic Reaction Networks Resembling the Biologically Relevant Krebs Cycle
Source: Angew Chem Int Ed Engl. 2025 Nov 26;65(4):e16853. doi: 10.1002/anie.202516853 (PMC12828449; doi:10.1002/anie.202516853)
Supplement: Supplementary file 1 — Supporting Information [file ANIE-65-e16853-s001.pdf]

Supporting Information

Influence of Rare-Earth-Elements on Prebiotic Reaction Networks Resembling the Biologically Relevant Krebs Cycle

Jonathan Gutenthaler-Tietze,[a] Carolina G. Heßler,[a] Lena J. Daumann*[a]

[a]Chair of Bioinorganic Chemistry

Heinrich-Heine-Universität Düsseldorf

40225 Düsseldorf, Germany

Lena.daumann@hhu.de

Table of Contents

General Remarks 2

Analytical Methods 2

NMR Spectroscopy 2

Mass Spectrometry 2

Synthetic Procedures 3

Synthesis of Tricarballylate 3

Synthesis of Parapyruvic Acid 3

Derivatization 3

Reactions 4

Analytical Data 8

NMR Spectra 8

GC-MS Data 35

Rare-earth Chlorides *vs.* $FeCl_2$ 44

IR Spectra 47

Comparison of the La^{3+}-mediated Reaction Network with the biological Krebs Cycle 49

References 50

General Remarks

α-Hydroxyketoglutarate was synthesized following a literature procedure[1] and tricarballylic acid[2] and parapyruvic acid[3] were synthesized following modified literature procedures. All other chemicals were purchased from commercial sources and used as received. Ultrapure water (type 1, pH 5.6, 18.2 MΩ·cm at 25 °C) was used for all experiments. To this end, deionized water was further purified using a Synergy® UV system from Merck Millipore®. Measurements of pH were realized with a Mettler Toledo FiveEasy Benchtop F20 pH/mV Meter in combination with either an InLab Micro pro ISM pH electrode or the standard kit electrode. ATR infrared spectra were recorded with a Perkin Elmer Spektrum Two FT-IR spectrometer. For inductively coupled plasma-optical emission spectroscopy (ICP-OES) an Agilent ICP-OES 5800 instrument linked to an Agilent SPS4 autosampler was used. Elemental analysis of the precipitate was performed on an Elementar vario micro cube.

Analytical Methods

NMR Spectroscopy

NMR spectra were recorded at the CeMSA@HHU (Center for Molecular and Structural Analytics @ Heinrich-Heine-Universität) either on a Bruker Avance III 600 or a Bruker Avance NEO Evo 600 spectrometer. Chemical shifts of reaction samples dissolved in mixtures of water and deuterium oxide are referenced to sodium 3-(trimethylsilyl)-propionate-2,2,3,3-d_4 (TMSP-d_4) as internal standard ($\delta_{\text{R-TMS}}$ = 0 ppm). Spectra were recorded at 25 °C. The signal of water was suppressed using the *zgesgp* pulse program (32 scans, pulse delay of 30 or 60 seconds) supplied by Bruker. Data analysis was performed with *MestReNova* 15.0.1 by Mestrelab Research S.L.

Mass Spectrometry

GC-MS analysis was performed on a GC System 7820A coupled to an Agilent 5977B EI mass spectrometer with helium as the carrier gas, supplied at a constant flow rate of 1 $mL \cdot min^{-1}$. An Agilent High Resolution Gas Chromatography Column (DB5–MS Ultra Inert, 30 m × 0.250 mm x 0.25 µm, SN US 3733365H) was used. The analysis was carried out on a 1 µL injection volume (split mode). Split ratio was 10:1 with a split flow of 10 $mL \cdot min^{-1}$. The injection port temperature was set to 250 °C, and the column oven temperature program was as follows: 1.) 60 °C for 1 min. 2.) temperature increased to 260 °C with a 25 $°C \cdot min^{-1}$ ramp. 3.) 4 min hold. The mass spectrometer was turned on after a 4 min delay and was operated at the electron ionization mode with a quadrupole temperature of 150 °C. Data was acquired in the full-scan mode (50-500). Data analysis and manual integration of peaks were performed using Agilent *Mass Hunter Workstation* v.B.08.00 software.

Synthetic Procedures

Synthesis of Tricarballylate

This synthesis was performed according to a modified literature procedure.[4]

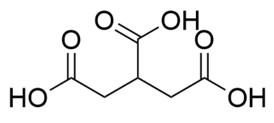

$C_6H_8O_6$
176.12 g·mol^{-1}

Aconitic acid (500 mg, 2.87 mmol, 1.00 equiv) was dissolved in THF (5 mL) in an argon-flushed Schlenk flask. Palladium on carbon (10wt%, 61.1 mg, 0.06 mmol, 0.02 equiv) was added and the atmosphere was changed to hydrogen (1 atm). The reaction mixture was stirred under H_2 atmosphere for 18 h and subsequently filtered through a celite pad. The filtrate was concentrated *in vacuo* and the residue recrystallized from a hexane/EtOAc/EtOH mixture. Colorless crystals could be obtained after a couple of days at −20 °C.

^{1}H NMR (300 MHz, DMSO-*d*$_6$) δ 12.29 (s, 3H, COO**H**), 2.94 (tt, $^3J_{H,HA}$ = 7.2 Hz, $^3J_{H,HB}$ = 6.1 Hz,1H, **H**-CCOOH), 2.56 (dd, $^3J_{HA,HB}$ = 16.7 Hz, $^3J_{HA,H}$ = 7.2 Hz, 2H, **H$_A$**-CH$_B$), 2.43 (dd, $^3J_{HB,HA}$ = 16.7 Hz, $^3J_{HB,H}$ = 6.1 Hz, 2H, **H$_B$**-CH$_A$) ppm.

Synthesis of Parapyruvic Acid

This synthesis was performed according to a modified literature procedure.[3]

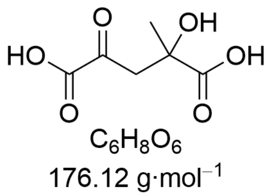

$C_6H_8O_6$
176.12 g·mol^{-1}

Sodium pyruvate (200 mg, 1.82 mmol, 2.00 equiv) was dissolved in water (5 mL). The pH of the reaction solution was adjusted to 12 with aqueous KOH solution (6 M) and the reaction mixture was stirred at room temperature. After 15 min, the pH was adjusted to 6 using sulfuric acid (1 M) and the solution was diluted with water to a final concentration of 3 mM, assuming quantitative conversion. NMR measurements showed 66% conversion.

^{1}H NMR (600 MHz, H_2O:D_2O, 9:1) δ 3.30 (d, $^2J_{HA,HB}$ = 18.0 Hz, 1H, **H$_A$**-CH$_B$), 3.17 (d, $^2J_{HB,HA}$ = 18.0 Hz, 1H, **H$_B$**-CH$_A$), 1.37 (s, 3H, C**H$_3$**).

Derivatization

To analyze the reaction mixture by GC-MS, the sample was first derivatized following a modified literature procedure.[1]

General Procedure (GP): In the first derivatization step, a 700 µL aliquot of the resuspended sample was added to 115±2 mg of Chelex® (sodium form) in a PP Eppendorf tube and the resulting mixture was shaken for 1 h (1000 rpm, 25 °C). Afterwards, 600 µL of the treated and centrifuged sample was transferred to a 15 mL falcon (to mitigate pressure build up during derivatization), subsequently mixed with 300 µL ethanol, 40 µL pyridine and 40 µL ECF and vortexed for 30 s. A second 40 µL portion of ECF was added, followed by vortex mixing for

30 s. For the extraction of the derivatized products, 200 µL $CHCl_3$ was added to the mixture and vortexed for 10 s. To this, 600 µL of a saturated aqueous $NaHCO_3$ solution was added and vortex mixed for 10 s. A portion of 150 µL was taken from the $CHCl_3$ layer and dried over anhydrous Na_2SO_4. Subsequently, 50 µL of the dried $CHCl_3$ layer was mixed with 150 µL of ethyl acetate and transferred *via* a syringe filter to a glass vial for GC-MS analysis.

Reactions

Comparison of $FeCl_2$ and REE chloride reactivity in 3 h reaction ($ScCl_3 \cdot 6\ H_2O$, $YCl_3 \cdot 6\ H_2O$, $LaCl_3 \cdot 7\ H_2O$, $CeCl_3 \cdot 7\ H_2O$, $PrCl_3 \cdot 6\ H_2O$, $NdCl_3 \cdot 6\ H_2O$, $EuCl_3 \cdot 6\ H_2O$, $HoCl_3 \cdot 6\ H_2O$, $LuCl_3 \cdot 6\ H_2O$)

Sodium pyruvate (11.0 mg, 0.10 mmol, 1.00 equiv) and glyoxylic acid monohydrate (18.4 mg, 0.20 mmol, 2.00 equiv) were added to a 15 mL pressure tube (Ace Glass Inc.) with 2.5 mL of water. The pH was adjusted to 6.5 with aqueous NaOH (1 M/0.1 M) and the volume adjusted to 3 mL. Subsequently, the metal salt (0.20 mmol, 2.00 equiv) was added and the pH was determined after thorough mixing. The reaction mixture was flushed with nitrogen for 1 min and the pressure tube was sealed with a PTFE plug seal. The reagents were put into a heating block heated to 70 °C and stirred for 3 h at that temperature. Afterwards, the reaction vessel was taken out of the heating block and once the reaction mixture had cooled to ambient temperature, 700 µL were removed and derivatized as described in the *GP*. Another 800 µL of the resuspended reaction mixture were added to 400±5 mg of Chelex® (sodium form) and subsequently shaken for 1 h (1000 rpm, 25 °C). For NMR measurements 450 µL of the resin-treated supernatant were added to 50 µL of D_2O containing 25 mM TMSP-d_4. To isolate the precipitate, 1000 µL from the resuspended mixture were centrifuged, the resulting pellet was washed with water and subsequently dried at 40 °C overnight for IR analysis.

Comparison of $FeSO_4$ and $La_2(SO_4)_3$ reactivity (time-resolved)

Two reaction vessels were prepared in an identical way for each metal salt. Sodium pyruvate (18.3 mg, 0.17 mmol, 1.00 equiv) and glyoxylic acid monohydrate (30.7 mg, 0.33 mmol, 2.00 equiv) were added to 4.5 mL of water. The pH was adjusted to 6.5 with aqueous NaOH (1 M/0.1 M) and the volume adjusted to 5 mL. Afterwards, the solution was transferred to a 15 mL pressure tube (Ace Glass Inc.) and $La_2(SO_4)_3 \cdot 13\ H_2O$ (66.7 mg, 0.83 mmol, 1.00 equiv of La^{3+}) was added. The iron-containing reaction mixture was prepared analogously with $FeSO_4 \cdot 7\ H_2O$ (46.3 mg, 0.17 mmol, 1.00 equiv of Fe^{2+}). The reaction mixtures were flushed with nitrogen for 1 min and the pressure tubes were each sealed with a PTFE plug seal. The reaction vessels were put into a heating block heated to 70 °C. One of the reaction vessels was stirred for 5 h and the other for 72 h in total. After 1 h, 3 h and 5 h, 700 µL were removed from the first vessel of each metal salt after cooling and derivatized as described in *GP* and

another 700 µL were taken out, treated with Chelex® analogous to the *GP* and subsequently prepared for NMR measurements by addition of 450 µL of the resin-treated supernatant to 50 µL of D_2O containing 25 mM TMSP-d_4. For the second vessel the analogous procedure was performed after 24 h, 48 h and 72 h. After the aliquots were taken, the remaining reaction mixture was flushed with nitrogen for 1 min and the vessel was resealed and put back into the heating block.

Reaction with varying equivalents of La^{3+} for 3 h

For each La^{3+}-concentration, sodium pyruvate (11.0 mg, 0.10 mmol, 1.00 equiv) and glyoxylic acid (18.4 mg, 0.20 mmol, 2.00 equiv) were added to a vessel with 2.5 mL of water. The pH was adjusted to 6.5 with aqueous NaOH (1 M/0.1 M) and the volume adjusted to 3 mL. Afterwards, the solution was transferred to a 15 mL pressure tube (Ace Glass Inc.) and $La_2(SO_4)_3 \cdot 13\ H_2O$ (0.25 equiv, 0.10 equiv, 0.05 equiv, 0.00 equiv) was added. The reaction mixture was flushed with nitrogen for 1 min and the pressure tube was sealed with a PTFE plug seal. The reaction vessels were put into a heating block heated to 70 °C and stirred for 3 h. Afterwards, the reaction mixture was cooled to room temperature and 450 µL of the supernatant were combined with 50 µL of D_2O containing 25 mM TMSP-d_4 for NMR measurements.

La^{3+}-containing 3 h reaction (1:1 glyoxylate:pyruvate ratio) with varying pH

For each starting pH value, sodium pyruvate (11.0 mg, 0.10 mmol, 1.00 equiv) and glyoxylic acid (9.2 mg, 0.10 mmol, 1.00 equiv) were added to a vessel with 2.5 mL of water. The pH was adjusted to 6.7 with aqueous NaOH (1 M/0.1 M). Afterwards, $La_2(SO_4)_3 \cdot 13\ H_2O$ (40.0 mg, 0.05 mmol, 0.50 equiv) was added, the pH was adjusted (6.0, 7.0, 8.0) and the reaction mixture was topped up to 3 mL. Subsequently, the reaction mixture was transferred to a 15 mL pressure tube (Ace Glass Inc.), flushed with nitrogen for 1 min and the pressure tube was sealed with a PTFE plug seal. The reaction vessel was put into a heating block heated to 70 °C and stirred for 3 h. Afterwards, the reaction mixture was cooled to room temperature and the pH was checked ($pH_{start} \rightarrow pH_{end}$: 6.0 → 4.8, 7.0 → 6.9, 8.0 → 8.0). Then, 450 µL of the supernatant were combined with 50 µL of D_2O containing 25 mM TMSP-d_4 for NMR measurements. The resulting precipitate was centrifuged (10.000 rpm, 5 min), washed with water, centrifuged again and subsequently collected on a piece of filter paper to dry in air over night for the IR measurements.

Test of increased amount Chelex during workup of 72 h reaction

Identical to the time-resolved reaction mentioned earlier, two reaction vessels were prepared in the same way. Sodium pyruvate (18.3 mg, 0.17 mmol, 1.00 equiv) and glyoxylic acid

(30.7 mg, 0.33 mmol, 2.00 equiv) were added to 4.5 mL of water. The pH was adjusted to 6.5 with aqueous NaOH (1 M/0.1 M) and the volume adjusted to 5 mL. Afterwards, the solution was transferred to a 15 mL pressure tube (Ace Glass Inc.) and $La_2(SO_4)_3 \cdot 13\ H_2O$ (66.7 mg, 0.83 mmol, 1.00 equiv of La^{3+}) was added. The iron-containing reaction mixture was prepared analogously with $FeSO_4 \cdot 7\ H_2O$ (46.3 mg, 0.17 mmol, 1.00 equiv of Fe^{2+}). The reaction mixtures were flushed with nitrogen for 1 min and the pressure tubes were each sealed with a PTFE plug seal. The reaction vessels were put into a heating block heated to 70 °C. After 72 h the vessels were cooled to room temperature and a 800 µL aliquot of each of the resuspended reaction mixtures was added to 400±5 mg of Chelex® 100 resin (sodium form) and the resulting mixtures were shaken at 25 °C for 60 min at 1000 rpm. Then, 450 µL of the supernatants of each reaction were combined with 50 µL of D_2O containing 25 mM TMSP-d_4 for NMR measurements.

Reaction with 10% La^{3+} for 72 h

Sodium pyruvate (18.3 mg, 0.17 mmol, 1.00 equiv) and glyoxylic acid (30.7 mg, 0.33 mmol, 2.00 equiv) were added to 4.5 mL of water. The pH was adjusted to 6.5 with aqueous NaOH (1 M/0.1 M) and the volume adjusted to 5 mL. Afterwards, the solution was transferred to a 15 mL pressure tube (Ace Glass Inc.) and $La_2(SO_4)_3 \cdot 13\ H_2O$ (6.7 mg, 0.08 mmol, 0.10 equiv of La^{3+}) was added. Subsequently, the reaction mixture was transferred to a 15 mL pressure tube (Ace Glass Inc.), flushed with nitrogen for 1 min and the pressure tube was sealed with a PTFE plug seal. The reaction vessel was put into a heating block heated to 70 °C. After 72 h the vessels were cooled to room temperature and a 800 µL aliquot of each of the resuspended reaction mixtures was added to 400±5 mg of Chelex® 100 resin (sodium form) and the resulting mixtures were shaken at 25 °C for 60 min at 1000 rpm. Then, 450 µL of the supernatants of each reaction were combined with 50 µL of D_2O containing 25 mM TMSP-d_4 for NMR measurements.

Reaction with precipitate for 72 h

Sodium pyruvate (18.3 mg, 0.17 mmol, 1.00 equiv) and glyoxylic acid (30.7 mg, 0.33 mmol, 2.00 equiv) were added to 4.5 mL of water. The pH was adjusted to 6.5 with aqueous NaOH (1 M/0.1 M) and the volume adjusted to 5 mL. Afterwards, the solution was transferred to a 15 mL pressure tube (Ace Glass Inc.) and the La^{3+}-containing precipitate of a previous 72 h reaction (64.2 mg, corresponding to 0.17 mmol, 1.00 equiv La^{3+} according to elemental analysis) was added. Subsequently, the reaction mixture was transferred to a 15 mL pressure tube (Ace Glass Inc.), flushed with nitrogen for 1 min and the pressure tube was sealed with a PTFE plug seal. The reaction vessel was put into a heating block heated to 70 °C. After 72 h the vessels were cooled to room temperature and a 800 µL aliquot of each of the resuspended

reaction mixtures was added to 400±5 mg of Chelex® 100 resin (sodium form) and the resulting mixtures were shaken at 25 °C for 60 min at 1000 rpm. Then, 450 µL of the supernatants of each reaction were combined with 50 µL of D_2O containing 25 mM TMSP-d_4 for NMR measurements.

Reaction without addition of metal for 72 h with adjusted pH

Sodium pyruvate (18.3 mg, 0.17 mmol, 1.00 equiv) and glyoxylic acid (30.7 mg, 0.33 mmol, 2.00 equiv) were added to 4.5 mL of water. The pH was adjusted to 5.8 with aqueous NaOH (1 M/0.1 M) and the volume adjusted to 5 mL. Afterwards, the solution was transferred to a 15 mL pressure tube (Ace Glass Inc.). Subsequently, the reaction mixture was transferred to a 15 mL pressure tube (Ace Glass Inc.), flushed with nitrogen for 1 min and the pressure tube was sealed with a PTFE plug seal. The reaction vessel was put into a heating block heated to 70 °C. After 72 h the vessels were cooled to room temperature and a 800 µL aliquot of each of the resuspended reaction mixtures was added to 400±5 mg of Chelex® 100 resin (sodium form) and the resulting mixtures were shaken at 25 °C for 60 min at 1000 rpm. Then, 450 µL of the supernatants of each reaction were combined with 50 µL of D_2O containing 25 mM TMSP-d_4 for NMR measurements.

Reaction of La^{3+} with glyoxylate only for 3 h

Glyxoylic acid (9.2 mg, 0.10 mmol, 1.00 equiv) and $La_2(SO_4)_3{\cdot}13\ H_2O$ (40.0 mg, 0.05 mmol, 0.50 equiv) were added to a vessel with 2.5 mL of water. The pH was adjusted to 5.7 with aqueous NaOH (1 M/0.1 M) and the reaction mixture was subsequently topped up to 3 mL. The reaction mixture was transferred to a 15 mL pressure tube (Ace Glass Inc.), flushed with nitrogen for 1 min and the pressure tube was sealed with a PTFE plug seal. The reaction vessel was put into a heating block heated to 70 °C and stirred for 3 h. Afterwards, the reaction mixture was cooled to room temperature and 450 µL of the supernatant were combined with 50 µL of D_2O containing 25 mM TMSP-d_4 for NMR measurements.

Reaction of La^{3+} with pyruvate only for 3 h

Sodium pyruvate (11.0 mg, 0.10 mmol, 1.00 equiv) and $La_2(SO_4)_3 \cdot 13\ H_2O$ (40.0 mg, 0.05 mmol, 0.50 equiv) were added to a vessel with 2.5 mL of water. The pH was adjusted to 5.7 with aqueous NaOH (1 M/0.1 M) and the reaction mixture was subsequently topped up to 3 mL. The reaction mixture was transferred to a 15 mL pressure tube (Ace Glass Inc.), flushed with nitrogen for 1 min and the pressure tube was sealed with a PTFE plug seal. The reaction vessel was put into a heating block heated to 70 °C and stirred for 3 h. Afterwards, the reaction mixture was cooled to room temperature and 450 µL of the supernatant were combined with 50 µL of D_2O containing 25 mM TMSP-d_4 for NMR measurements.

Analytical Data

NMR Spectra

Reference Spectra

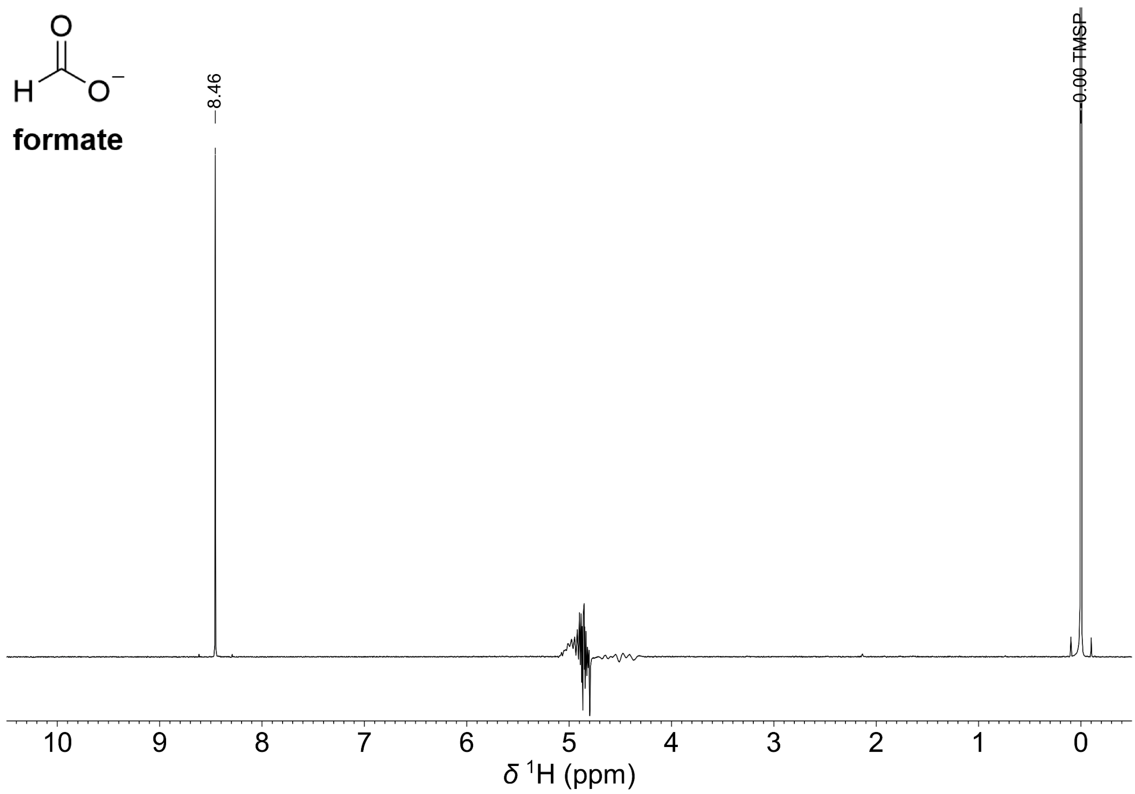

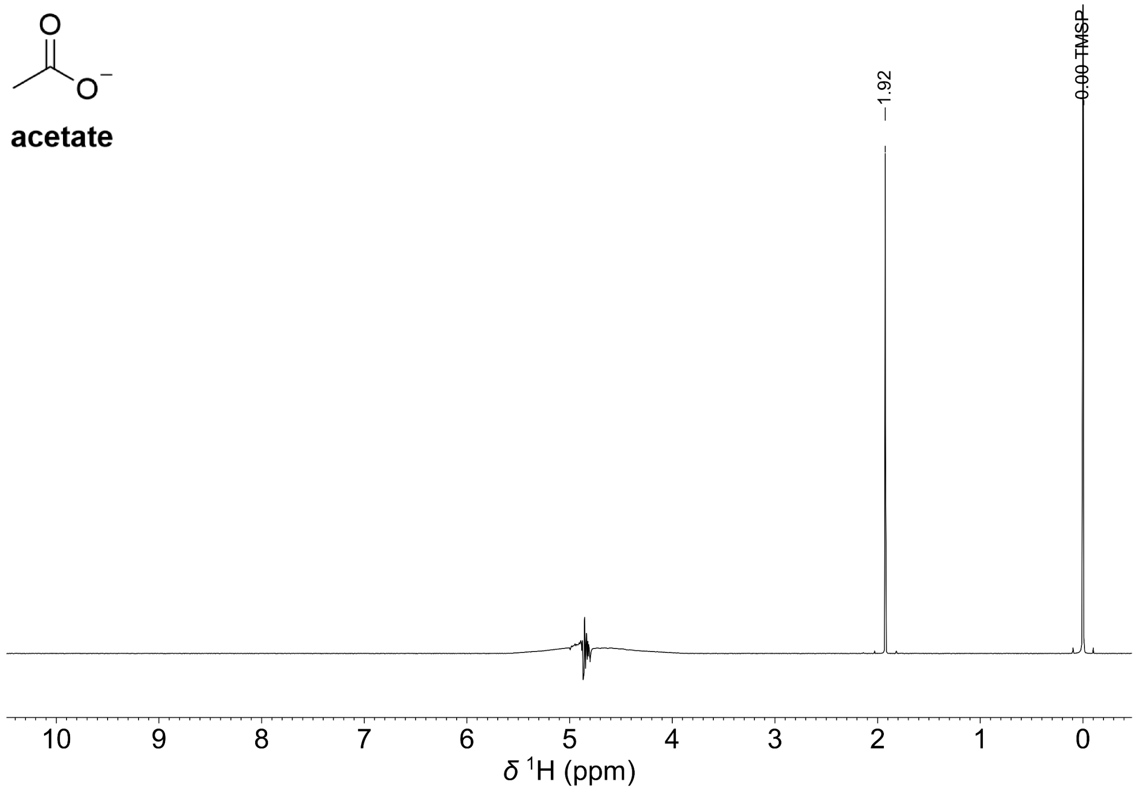

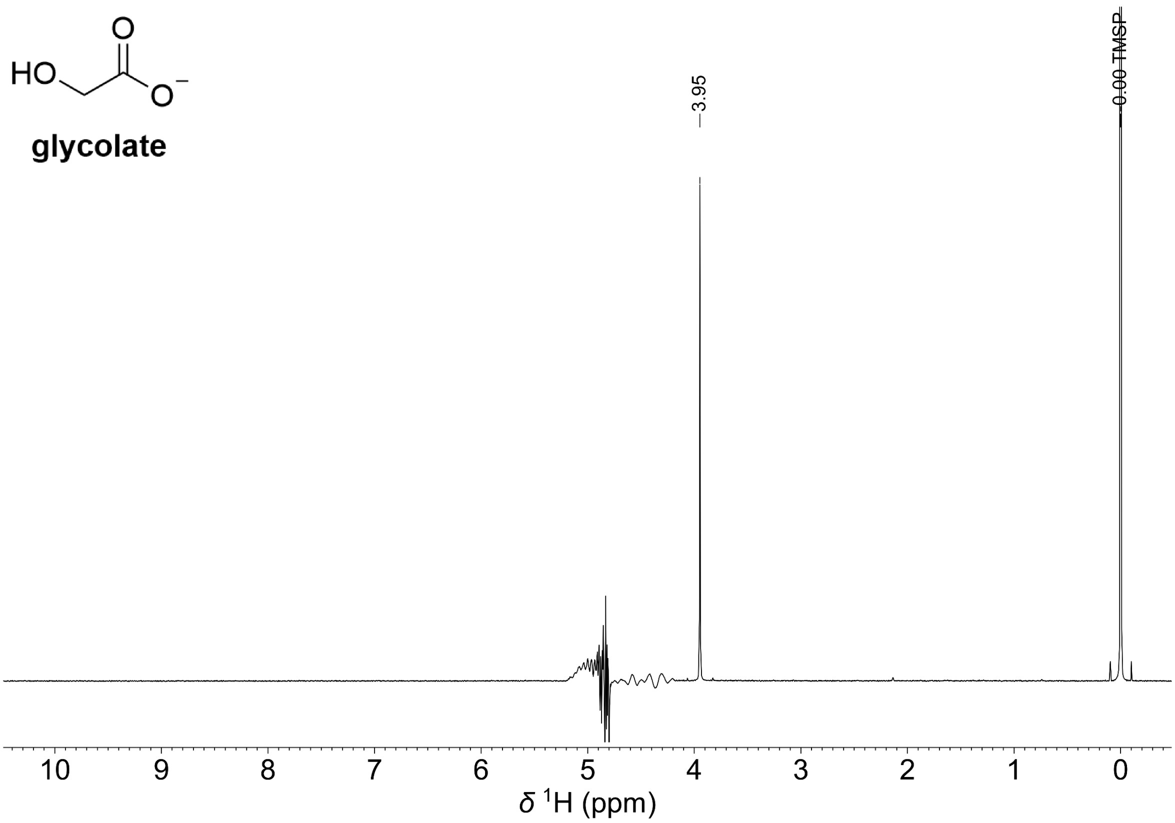
HO
O
O−
glycolate
3.95
0.00 TMSP
10 9 8 7 6 5 4 3 2 1 0
δ 1H (ppm)

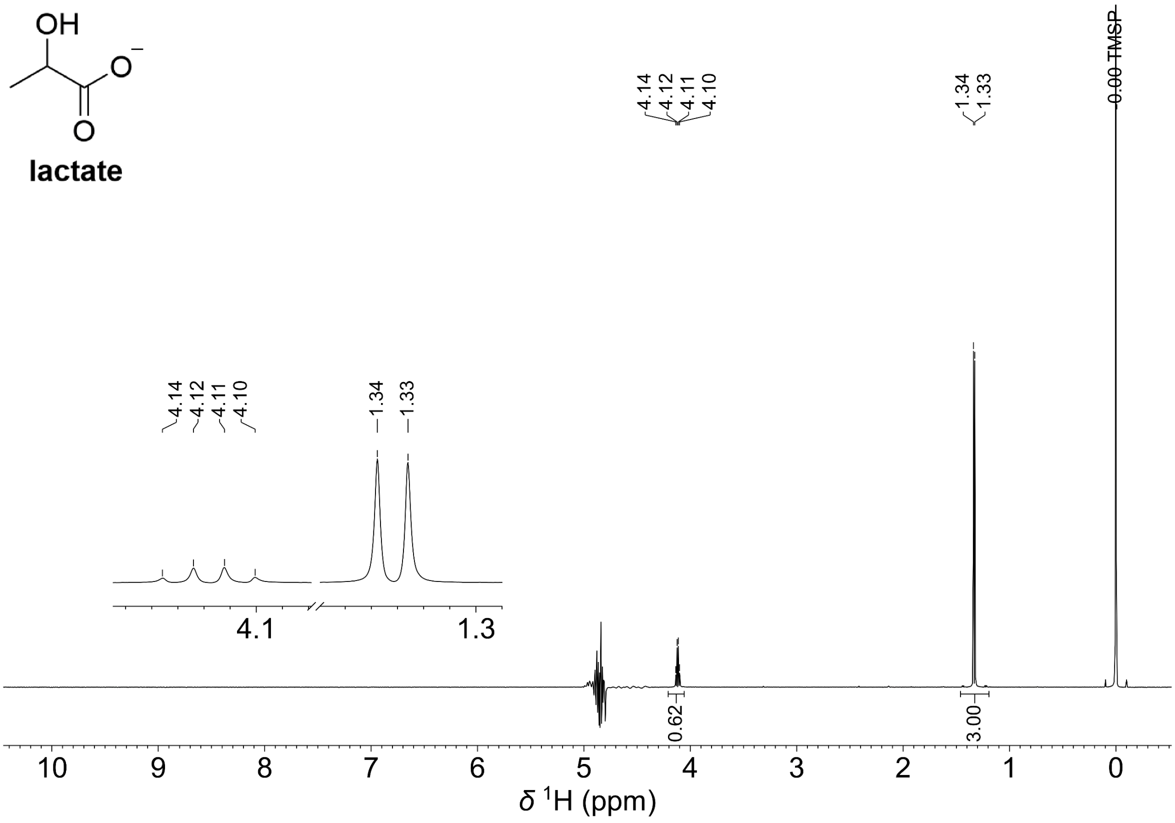
OH
O−
O
lactate
4.14
4.12
4.11
4.10
1.34
1.33
0.00 TMSP
4.1
1.3
0.62
3.00
10 9 8 7 6 5 4 3 2 1 0
δ 1H (ppm)

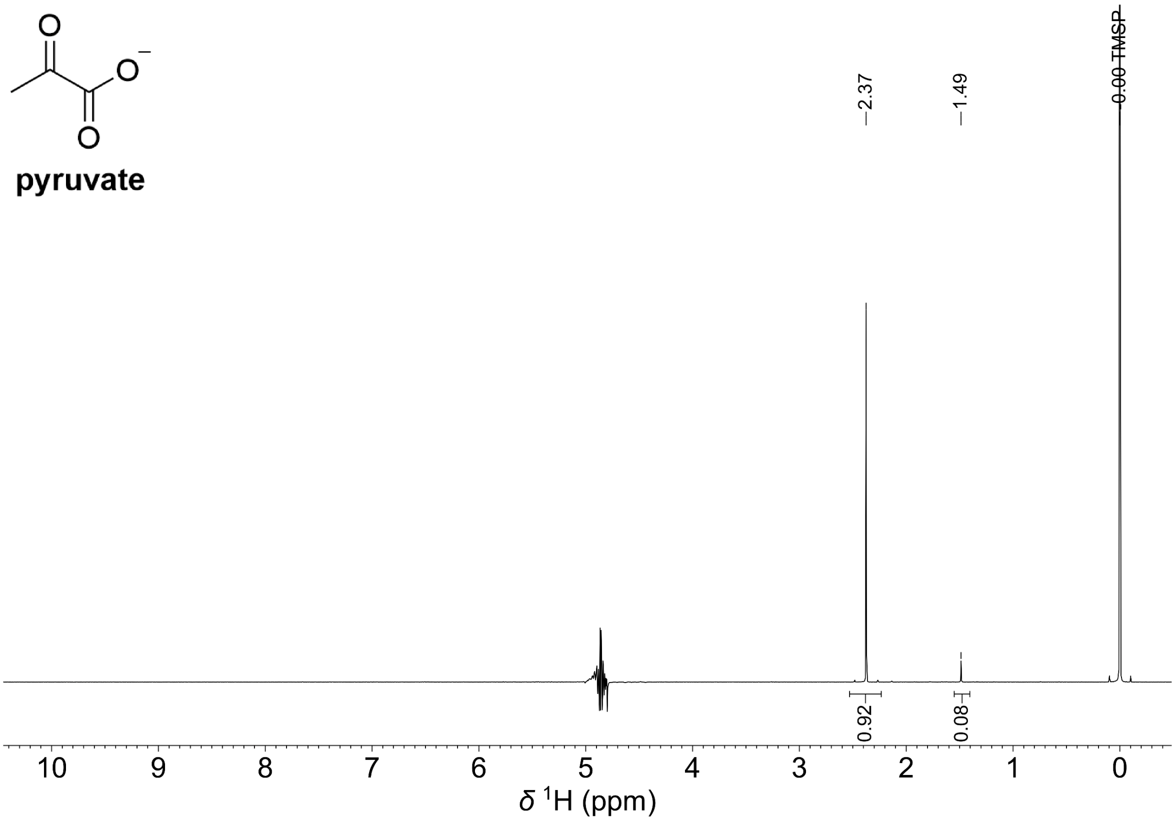
pyruvate
2.37
1.49
0.00 TMSP
0.92
0.08
10 9 8 7 6 5 4 3 2 1 0
δ 1H (ppm)

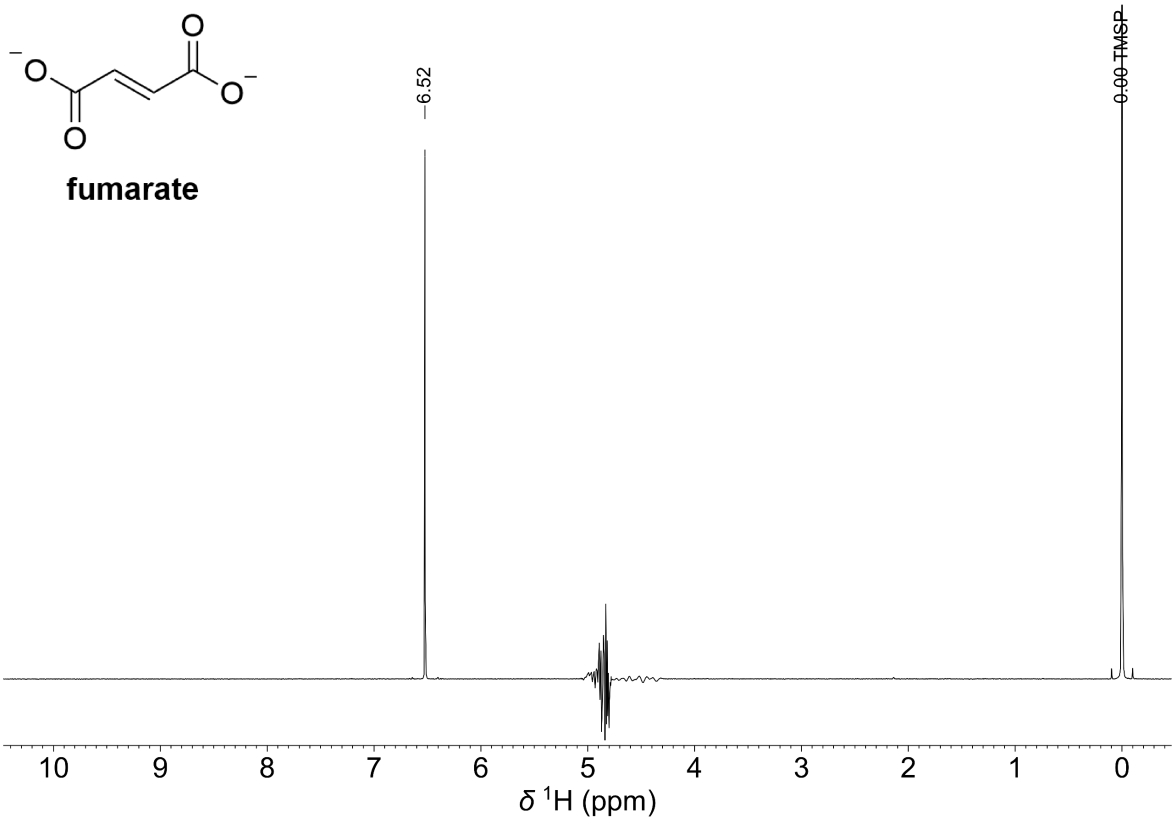
fumarate
6.52
0.00 TMSP
10 9 8 7 6 5 4 3 2 1 0
δ 1H (ppm)

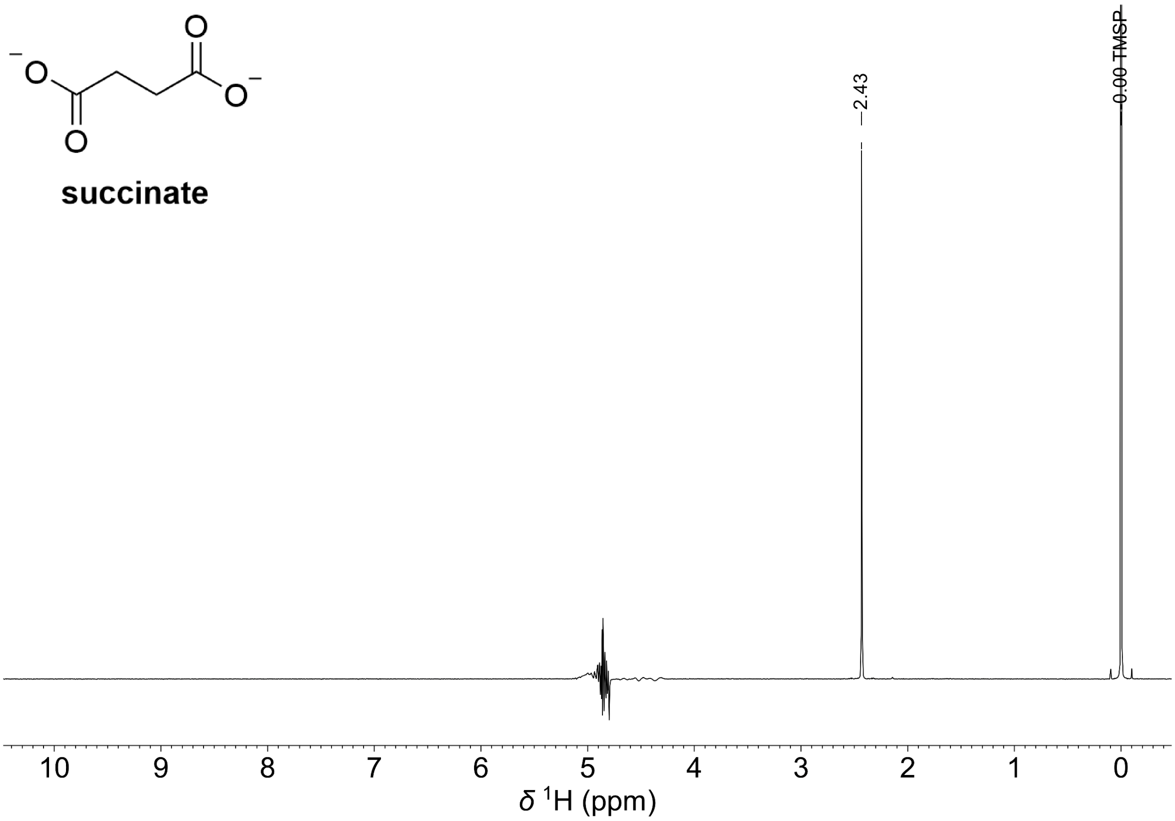
succinate
2.43
0.00 TMSP
10 9 8 7 6 5 4 3 2 1 0
δ ¹H (ppm)

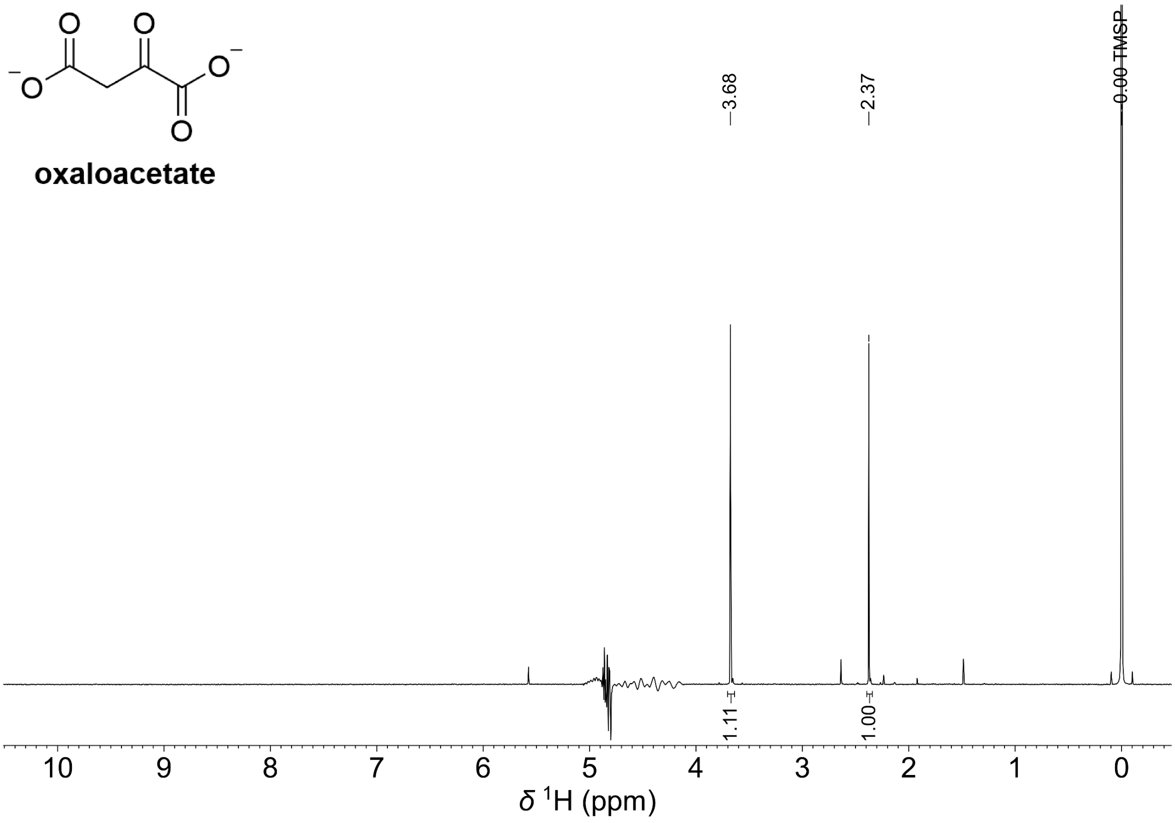
oxaloacetate
3.68
2.37
0.00 TMSP
1.11
1.00
10 9 8 7 6 5 4 3 2 1 0
δ ¹H (ppm)

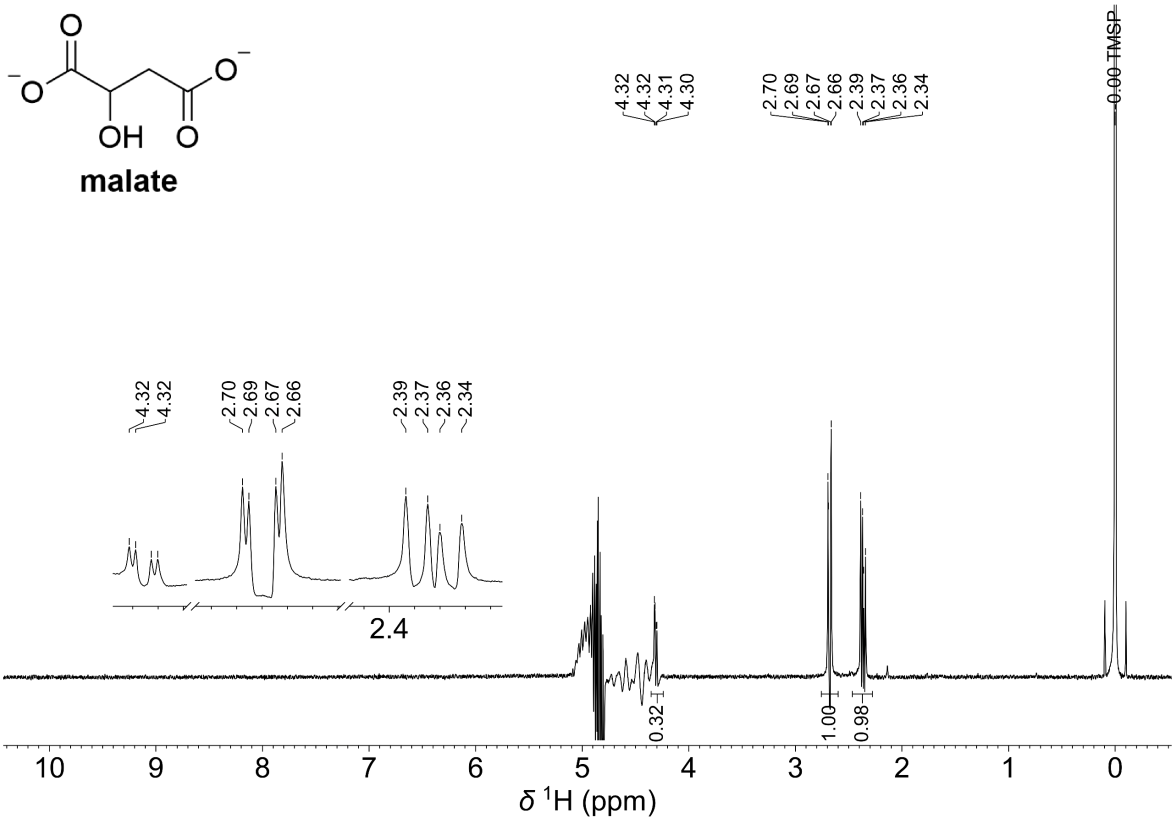

malate
OH
4.32 4.32 4.31 4.30
2.70 2.69 2.67 2.66 2.39 2.37 2.36 2.34
0.00 TMSP
4.32 4.32
2.70 2.69 2.67 2.66
2.39 2.37 2.36 2.34
2.4
0.32
1.00
0.98
10 9 8 7 6 5 4 3 2 1 0
δ 1H (ppm)

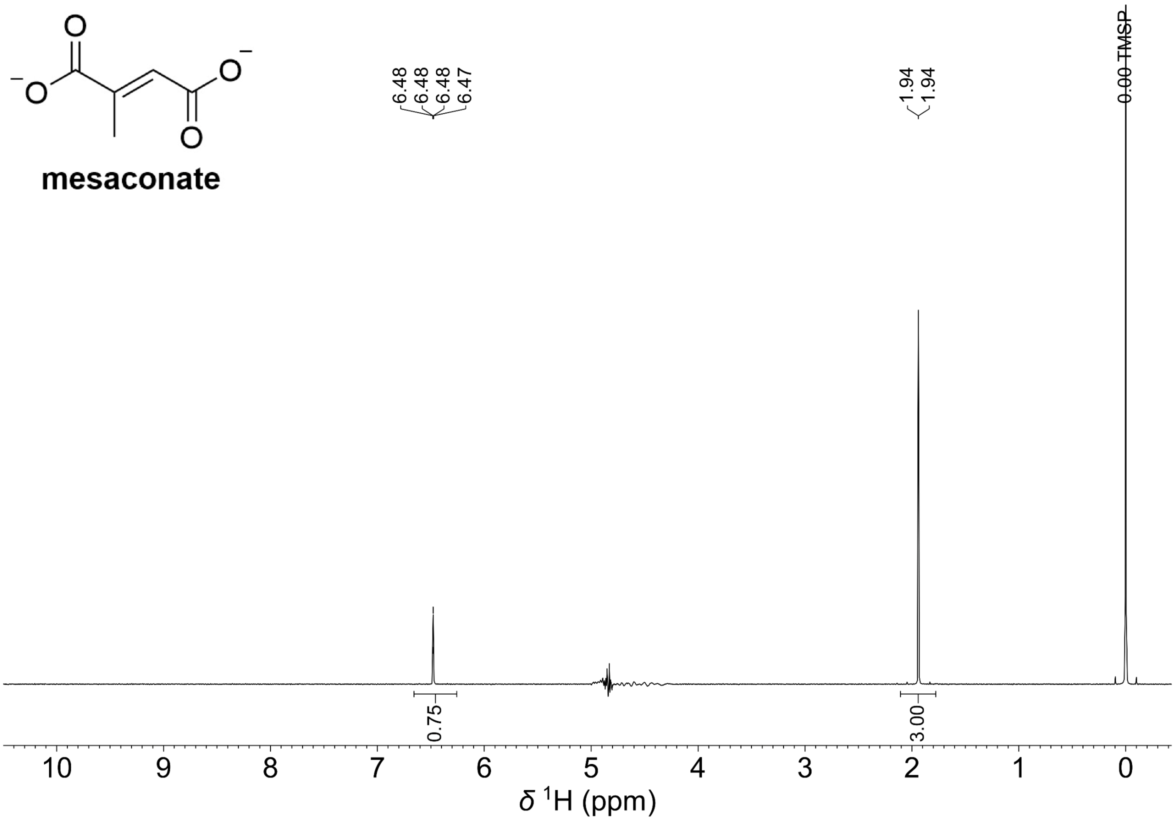

mesaconate
6.48 6.48 6.48 6.47
1.94 1.94
0.00 TMSP
0.75
3.00
10 9 8 7 6 5 4 3 2 1 0
δ 1H (ppm)

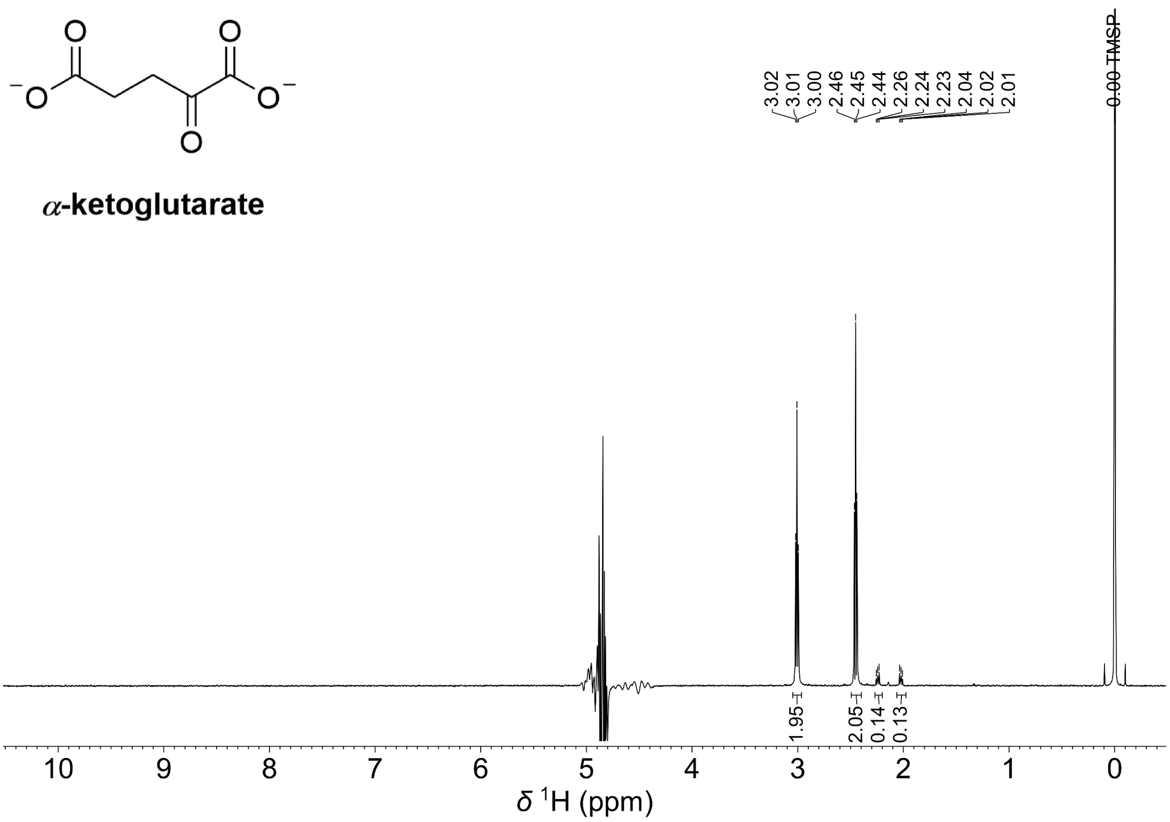
α-ketoglutarate
3.02
3.01
3.00
2.46
2.45
2.44
2.26
2.24
2.23
2.04
2.02
2.01
0.00 TMSP
1.95
2.05
0.14
0.13
10 9 8 7 6 5 4 3 2 1 0
δ 1H (ppm)

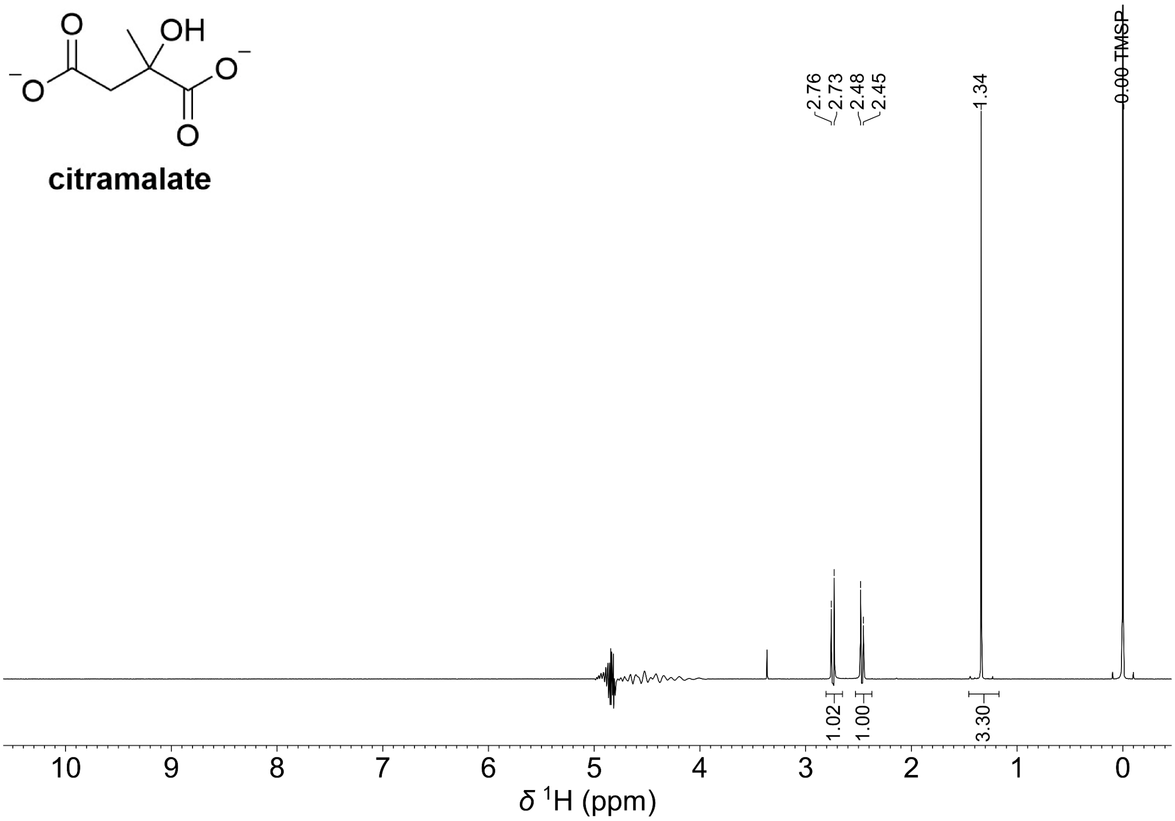
citramalate
2.76
2.73
2.48
2.45
1.34
0.00 TMSP
1.02
1.00
3.30
10 9 8 7 6 5 4 3 2 1 0
δ 1H (ppm)

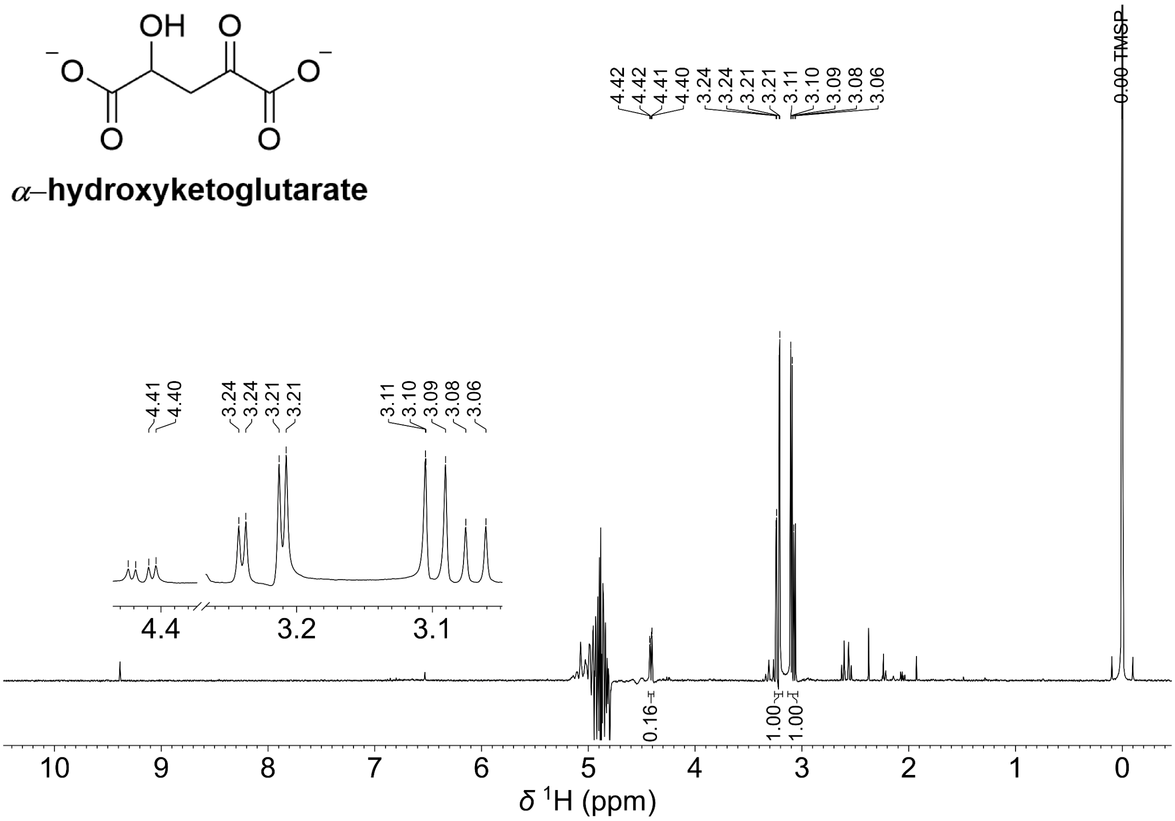
OH
O
α–hydroxyketoglutarate
4.42
4.42
4.41
4.40
3.24
3.24
3.21
3.21
3.11
3.10
3.09
3.08
3.06
0.00 TMSP
4.41
4.40
3.24
3.24
3.21
3.21
3.11
3.10
3.09
3.08
3.06
4.4
3.2
3.1
0.16
1.00
1.00
10
9
8
7
6
5
4
3
2
1
0
δ ¹H (ppm)

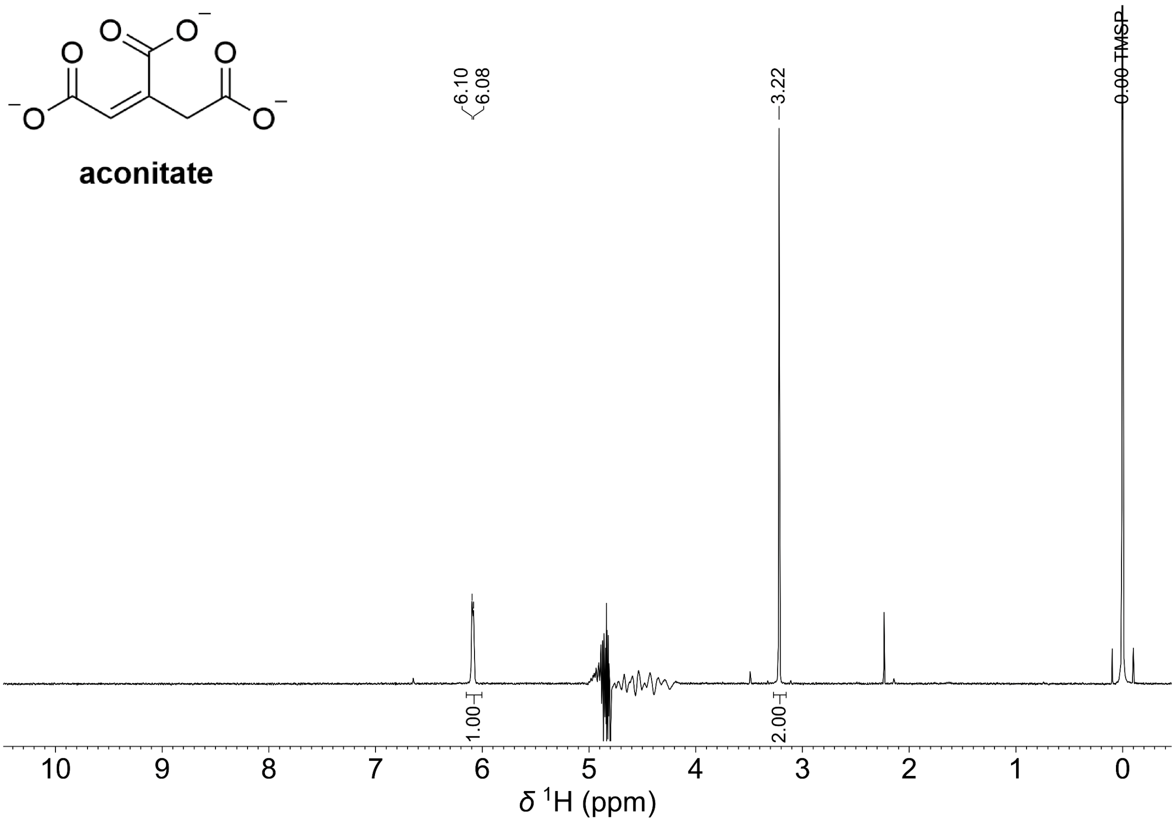
aconitate
6.10
6.08
3.22
0.00 TMSP
1.00
2.00
10
9
8
7
6
5
4
3
2
1
0
δ ¹H (ppm)

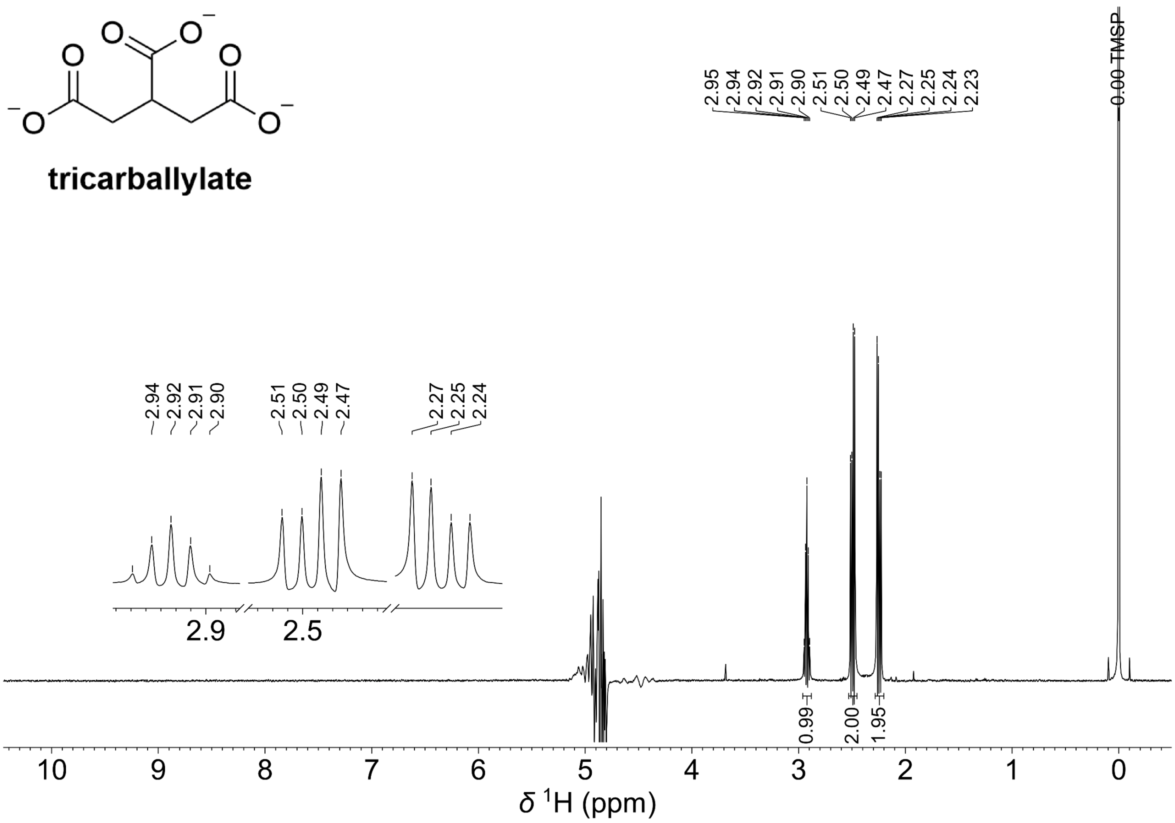
tricarballylate
2.95 2.94 2.92 2.91 2.90 2.51 2.50 2.49 2.47 2.27 2.25 2.24 2.23
0.00 TMSP
2.94 2.92 2.91 2.90
2.51 2.50 2.49 2.47
2.27 2.25 2.24
2.9 2.5
0.99 2.00 1.95
10 9 8 7 6 5 4 3 2 1 0
δ 1H (ppm)

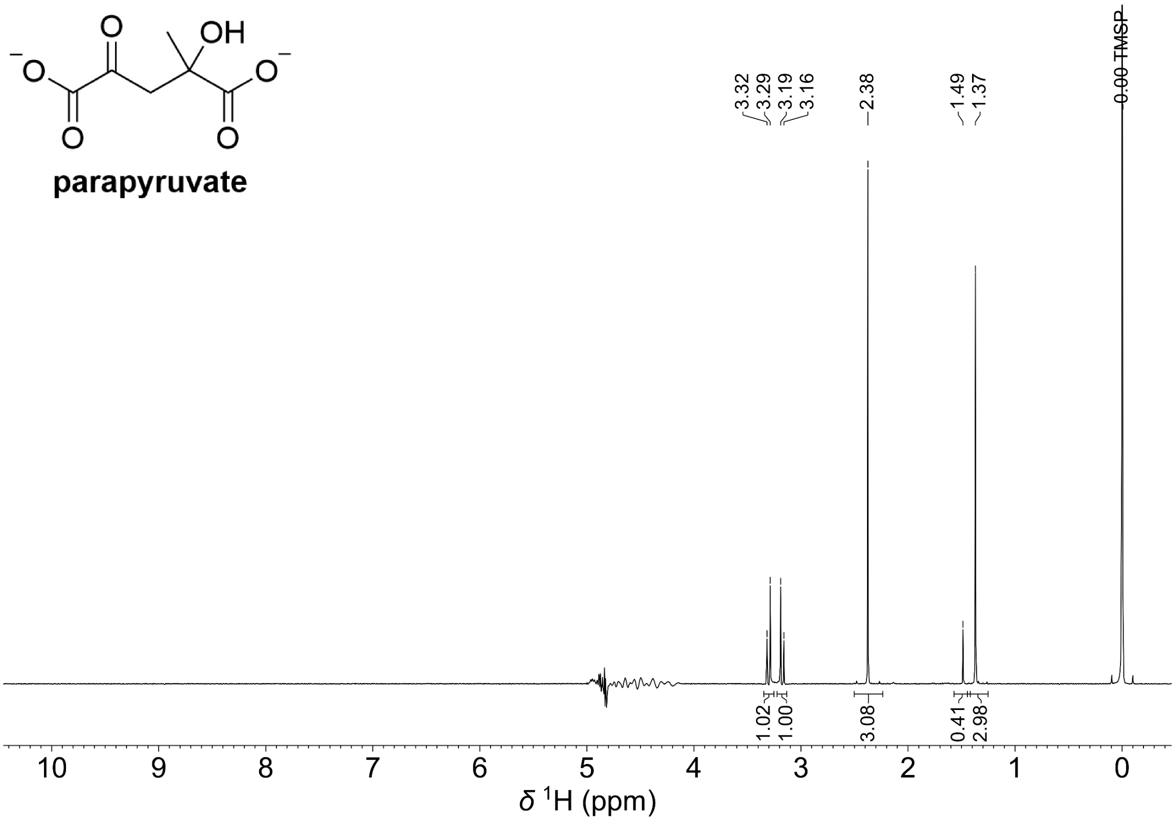
parapyruvate
3.32 3.29 3.19 3.16
2.38
1.49 1.37
0.00 TMSP
1.02 1.00 3.08 0.41 2.98
10 9 8 7 6 5 4 3 2 1 0
δ 1H (ppm)

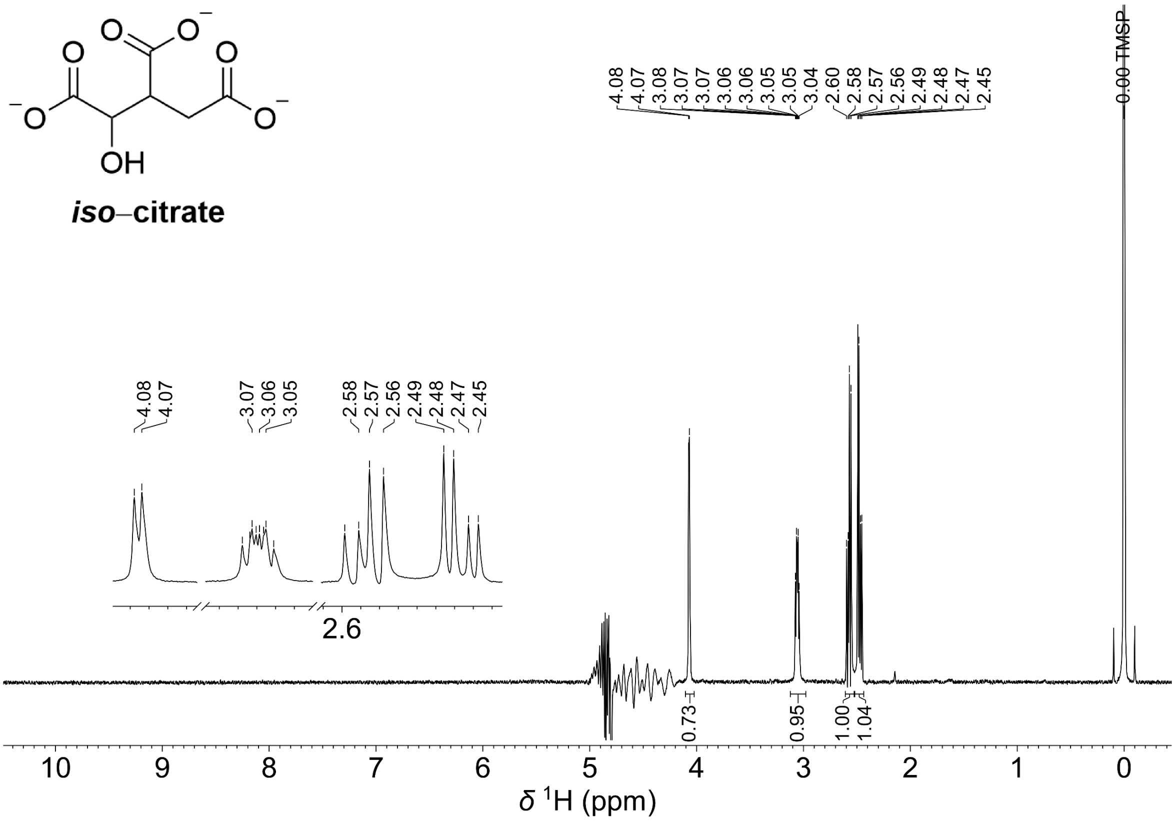

Figure S1. Reference ^{1}H NMR spectra of possible products in H_2O:D_2O 9:1 (pH 6) with 2.5 mM TMSP-d_4 as internal standard.

Table S1. Reference ^{1}H NMR shift of possible products in H_2O:D_2O 9:1 (pH 6) referenced to TMSP-d_4 as internal standard.

Compound	Chemical shifts (ppm) and coupling constants
**formate**	8.46 (s)
**acetate**	1.92 (s)
**glyoxylate**	5.08 (s)
**glycolate**	3.95 (s)
**pyruvate**	2.37 (s, pyruvate), 1.49 (s, pyruvate hydrate)
**lactate**	4.12 (q, $^3J_{H,H}$ = 7.1 Hz, 1H, **H**-COH), 1.33 (d, 7.1 Hz, 3H, C**H$_3$**)
**fumarate**	6.52 (s)
**succinate**	2.43 (s)
**oxaloacetate**	3.68 (s), 2.37 (s, pyruvate formed *via* *β*-decarboxylation)
**mesaconate**	6.48 (q, $^4J_{H,H}$ = 1.4 Hz, 1H, C**H**), 1.94 (d, $^4J_{H,H}$ = 1.4 Hz, 3H, C**H$_3$**)
**malate**	4.31 (dd, $^3J_{H,HB}$ = 10.4 Hz, $^3J_{H,HA}$ = 3.0 Hz, 1H, **H**-COH), 2.68 (dd, $^2J_{HA,HB}$ = 15.9 Hz, $^3J_{HA,H}$ = 3.0 Hz, 1H, **H$_A$**-CH$_B$), 2.36 (dd, $^2J_{HB,HA}$ = 15.9 Hz, $^3J_{HB,H}$ = 10.4 Hz, 1H, **H$_B$**-CH$_A$)
**citramalate**	2.74 (d, $^2J_{HA,HB}$ = 16.0 Hz, 1H, **H$_A$**-CH$_B$), 2.47 (d, $^2J_{HB,HA}$ = 15.8 Hz, 1H, **H$_B$**-CH$_A$), 1.34 (s, 2H, C**H$_3$**)
***α*-ketoglutarate**	3.01 (t, $^3J_{H,H}$ = 7.2 Hz, 2H, **H$_2$**CC(O)), 2.45 (t, $^3J_{H,H}$ = 7.2 Hz, 2H, **H$_2$**CCO$_2$),
***α*-hydroxyketoglutarate**	4.41 (dd, $^3J_{H,HB}$ = 8.9 Hz, $^3J_{H,HA}$ = 3.1 Hz, 1H, **H**-COH), 3.23 (dd, $^3J_{HA,HB}$ = 17.8 Hz, $^3J_{HA,H}$ = 3.1 Hz, 1H, **H$_A$**-CH$_B$), 3.08 (dd, $^3J_{HB,HA}$ = 17.8 Hz, $^3J_{HB,H}$ = 8.9 Hz, 1H **H$_B$**-CH$_A$)
**aconitate**	6.09 (s), 3.22 (s)
**tricarballylate**	2.92 (p$_{app}$, J_{app} = 7.7 Hz, 1H, **H**-CCO$_2$), 2.49 (dd, $^2J_{HA,HB}$ = 15.7, $^3J_{HA,H}$ = 8.0 Hz, 2H, **H$_A$**-CH$_B$), 2.25 (dd, $^2J_{HB,HA}$ = 15.7 Hz, $^3J_{HB,H}$ = 7.6 Hz, 2H, **H$_B$**-CH$_A$)
**parapyruvate**	3.30 (d, $^2J_{HA,HB}$ = 18.0 Hz, 1H, **H$_A$**-CH$_B$), 3.17 (d, $^2J_{HB,HA}$ = 18.0 Hz, 1H, **H$_B$**-CH$_A$), 1.37 (s, C**H$_3$**)
**isocitrate**	4.07 (d, $^3J_{H,H}$ = 4.9 Hz, 1H, **H**-COH), 3.06 (ddd, $^3J_{H,HA}$ = 9.2 Hz, $^3J_{H,HB}$ = 6.3 Hz, $^3J_{H,H}$ = 4.9 Hz, 1H, **H**-CCO$_2$), 2.58 (dd, $^3J_{HA,HB}$ = 15.8 Hz, $^3J_{HA,H}$ = 9.2 Hz, 1H, **H$_A$**-CH$_B$), 2.47 (dd, $^3J_{HB,HA}$ = 15.8 Hz, $^3J_{HB,H}$ = 6.3 Hz, 1H, **H$_B$**-CH$_A$)

Rare Earth Chlorides vs. $FeCl_2$ vs. no Metal

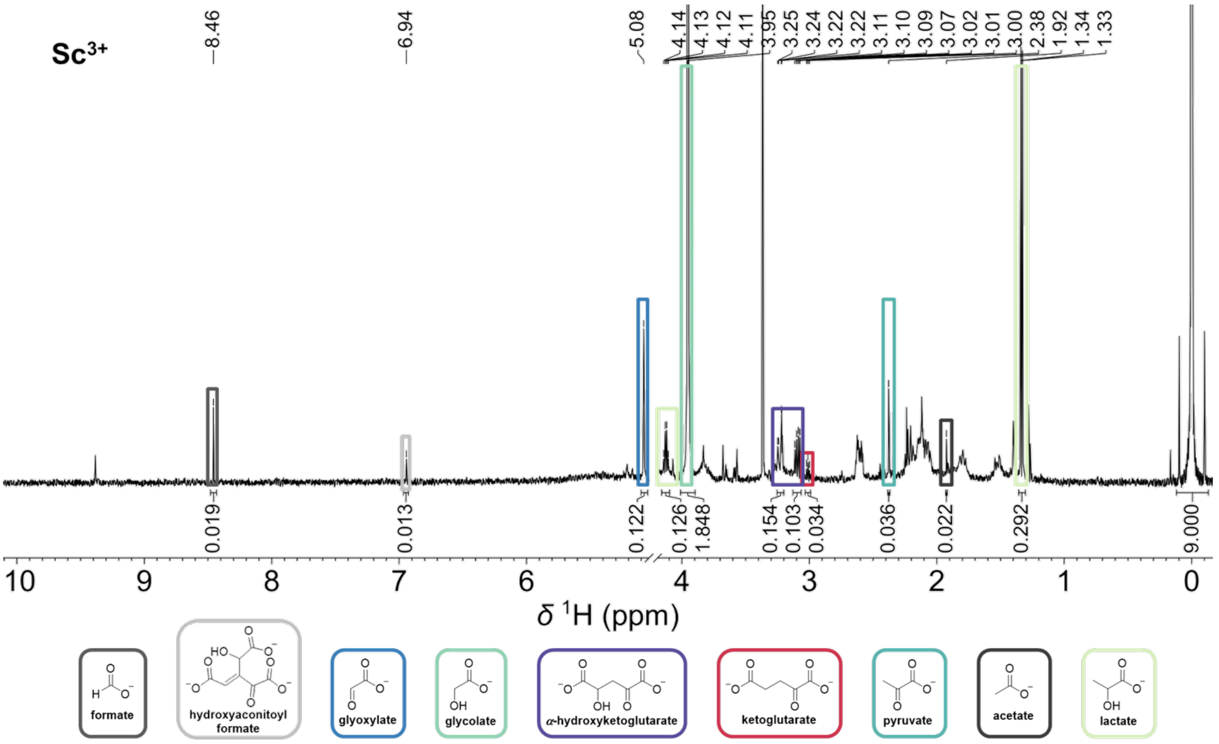

Figure S2. ^{1}H NMR spectrum with integral values of the reaction mixture containing $ScCl_3$ after 3 h at 70 °C with the assignable products marked in boxes and the corresponding structures marked with the same color below.

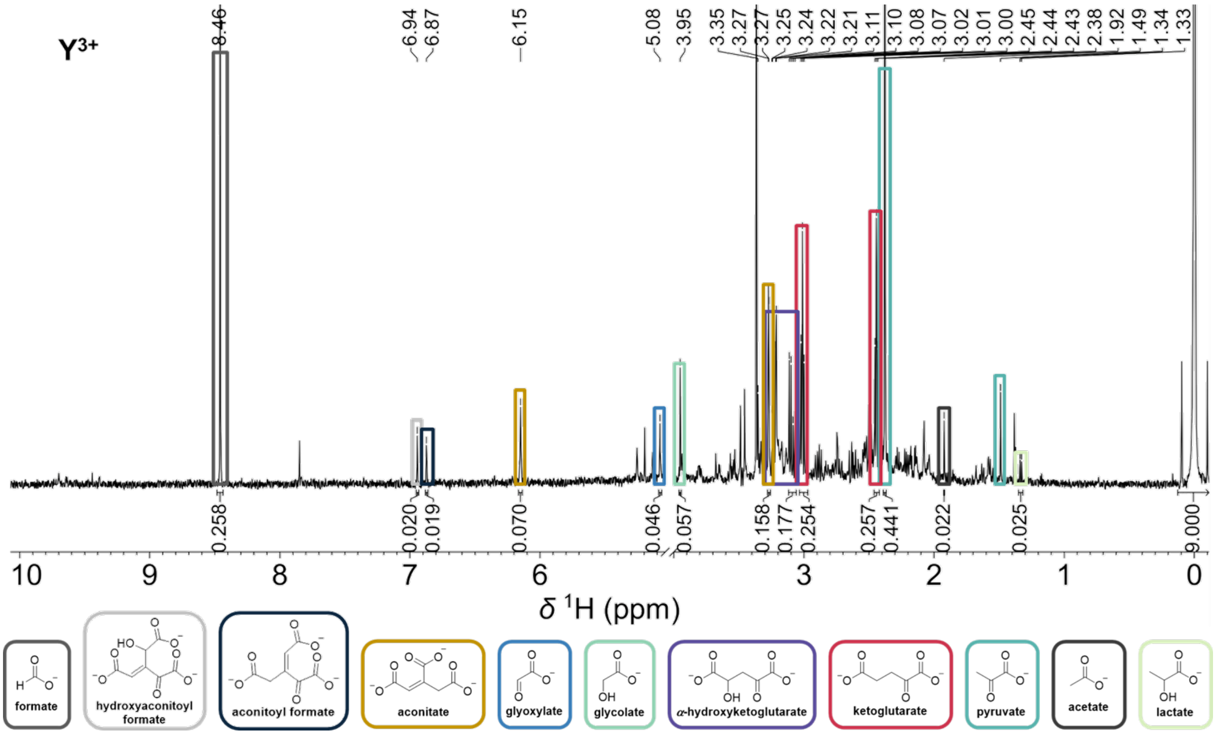

Figure S3. ^{1}H NMR spectrum with integral values of the reaction mixture containing YCl_3 after 3 h at 70 °C with the assignable products marked in boxes and the corresponding structures marked with the same color below.

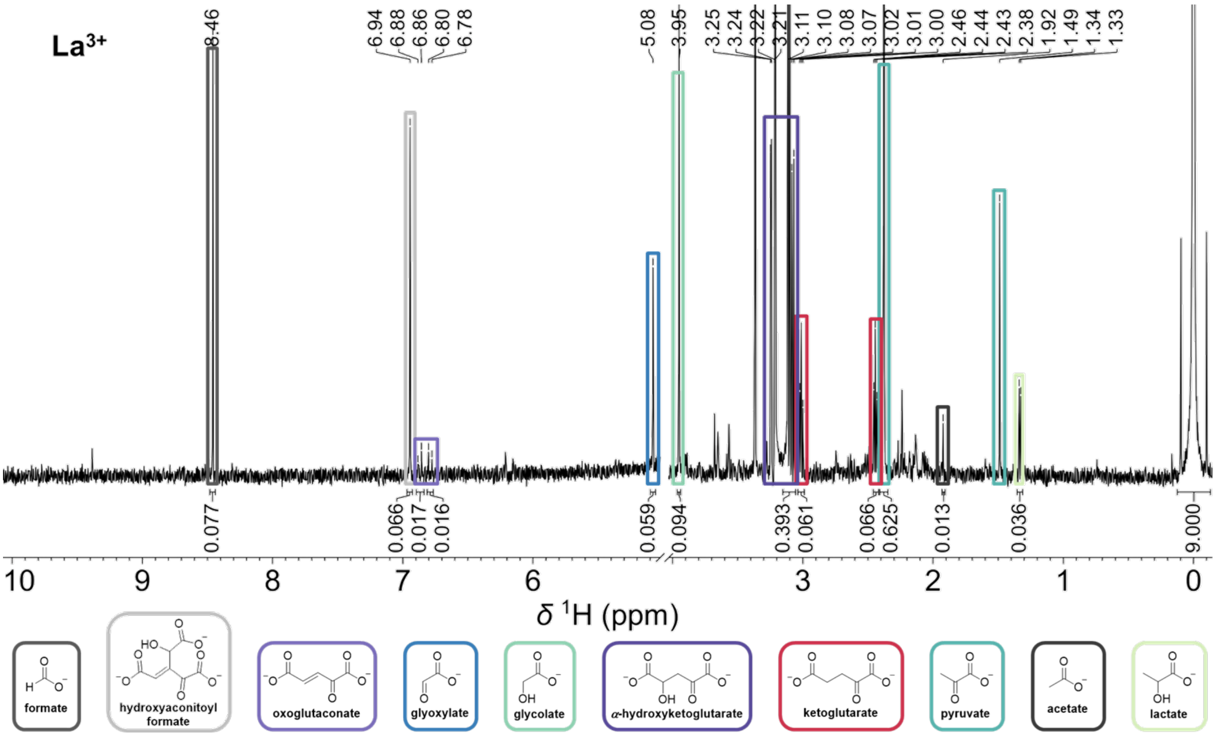

Figure S4. ^{1}H NMR spectrum with integral values of the reaction mixture containing $LaCl_3$ after 3 h at 70 °C with the assignable products marked in boxes and the corresponding structures marked with the same color below.

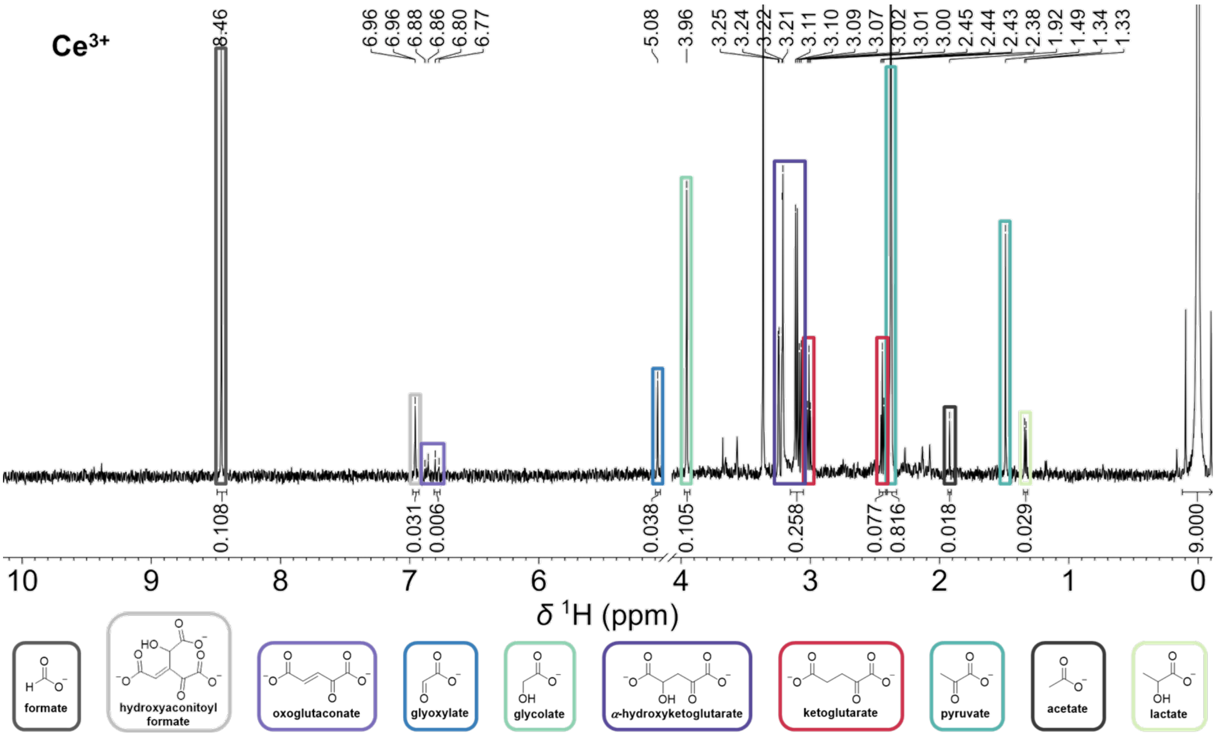

Figure S5. ^{1}H NMR spectrum with integral values of the reaction mixture containing $CeCl_3$ after 3 h at 70 °C with the assignable products marked in boxes and the corresponding structures marked with the same color below.

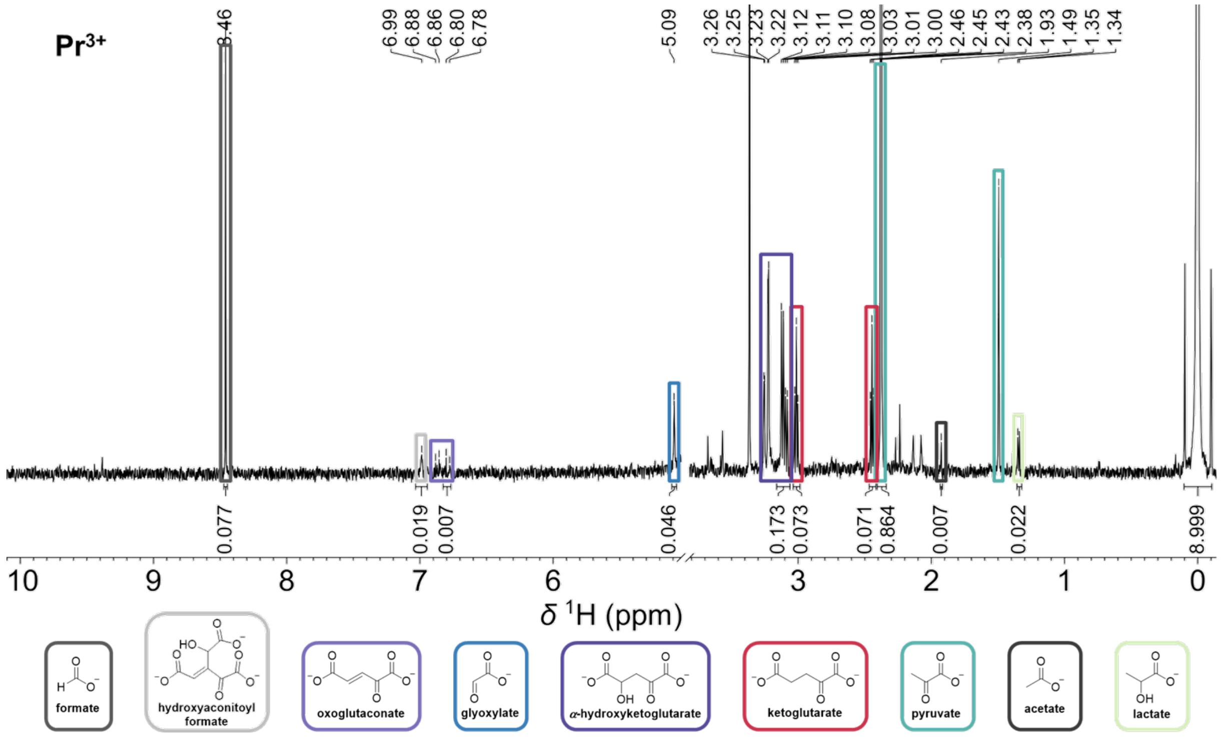

Figure S6. ^1H NMR spectrum with integral values of the reaction mixture containing $PrCl_3$ after 3 h at 70 °C with the assignable products marked in boxes and the corresponding structures marked with the same color below.

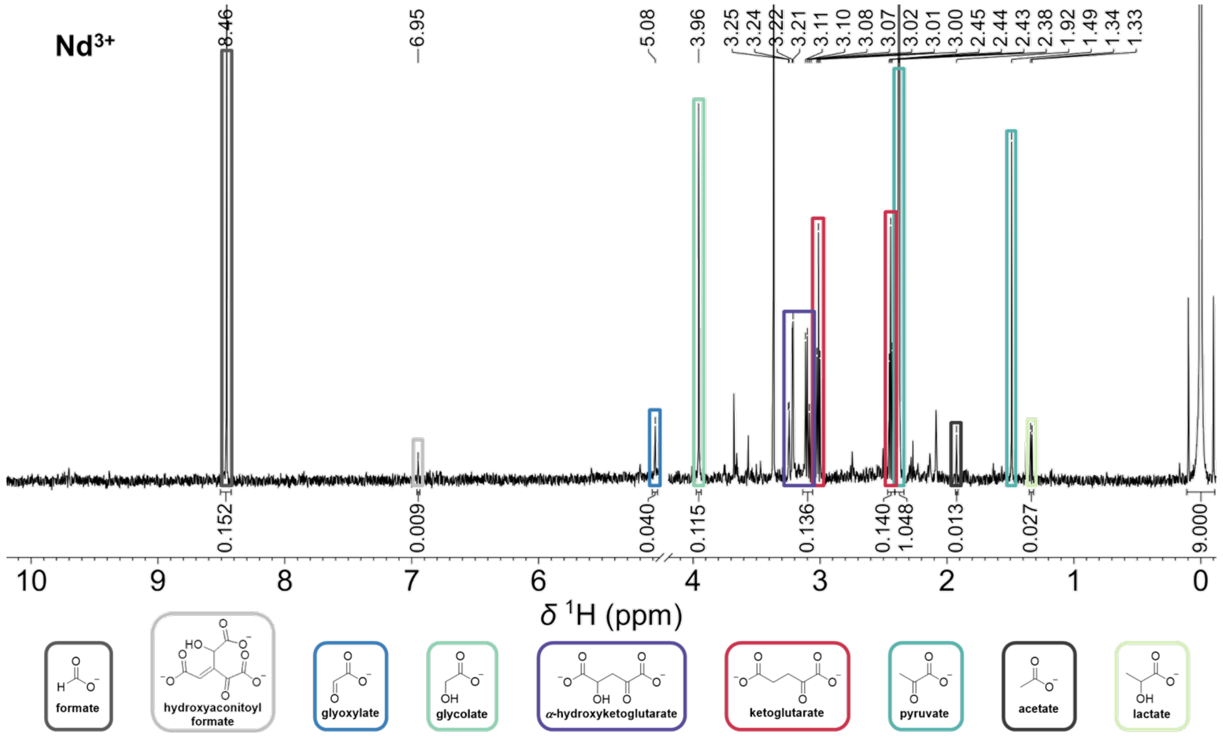

Figure S7. ^1H NMR spectrum with integral values of the reaction mixture containing $NdCl_3$ after 3 h at 70 °C with the assignable products marked in boxes and the corresponding structures marked with the same color below.

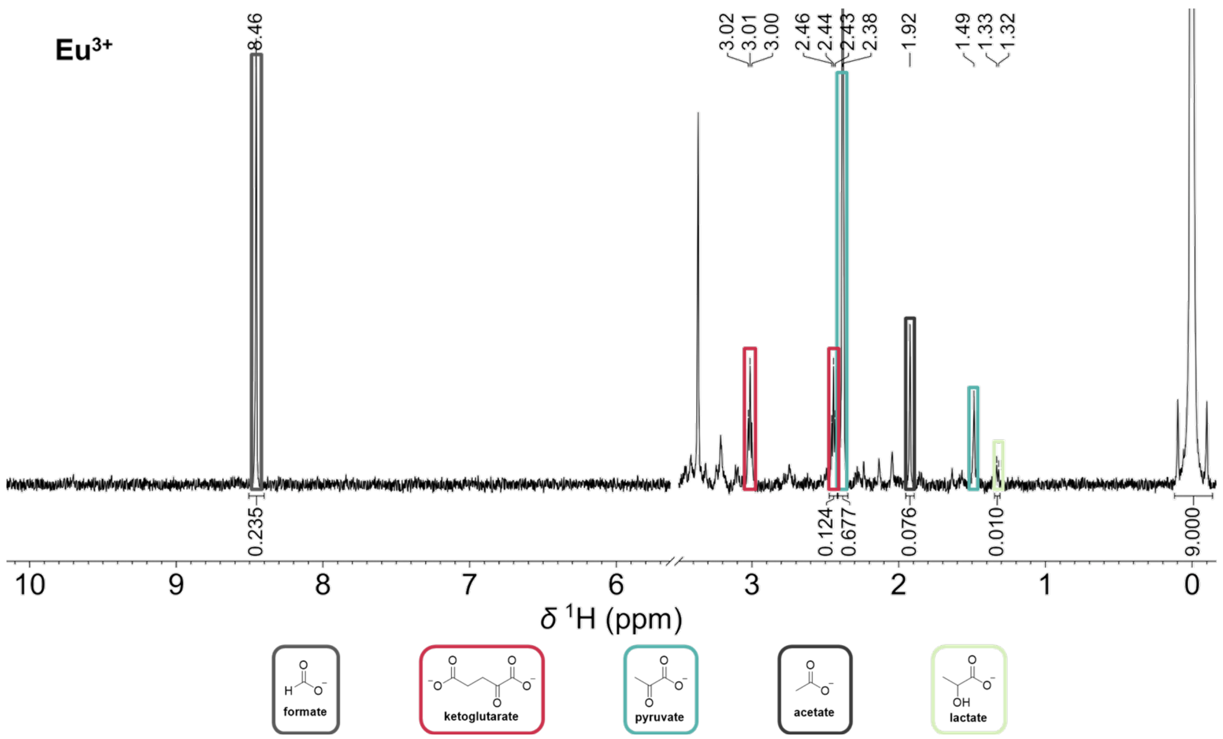

Figure S8. ^{1}H NMR spectrum with integral values of the reaction mixture containing $EuCl_3$ after 3 h at 70 °C with the assignable products marked in boxes and the corresponding structures marked with the same color below.

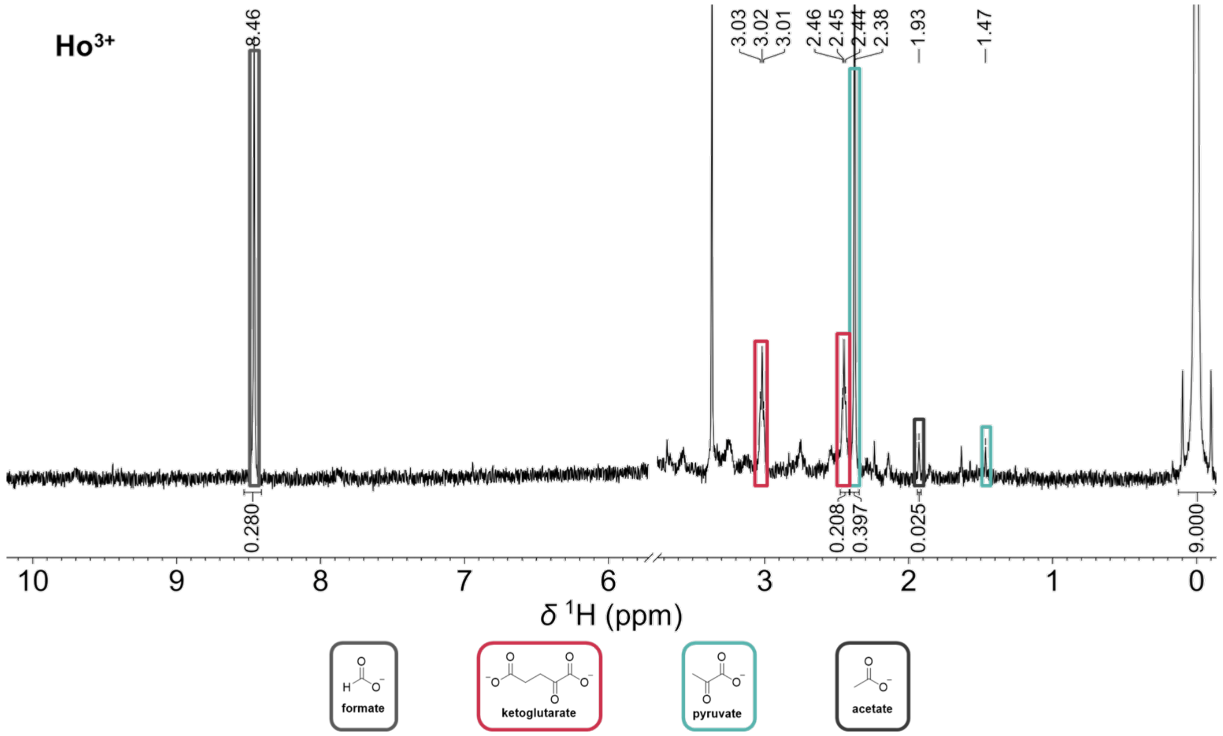

Figure S9. ^{1}H NMR spectrum with integral values of the reaction mixture containing $HoCl_3$ after 3 h at 70 °C with the assignable products marked in boxes and the corresponding structures marked with the same color below.

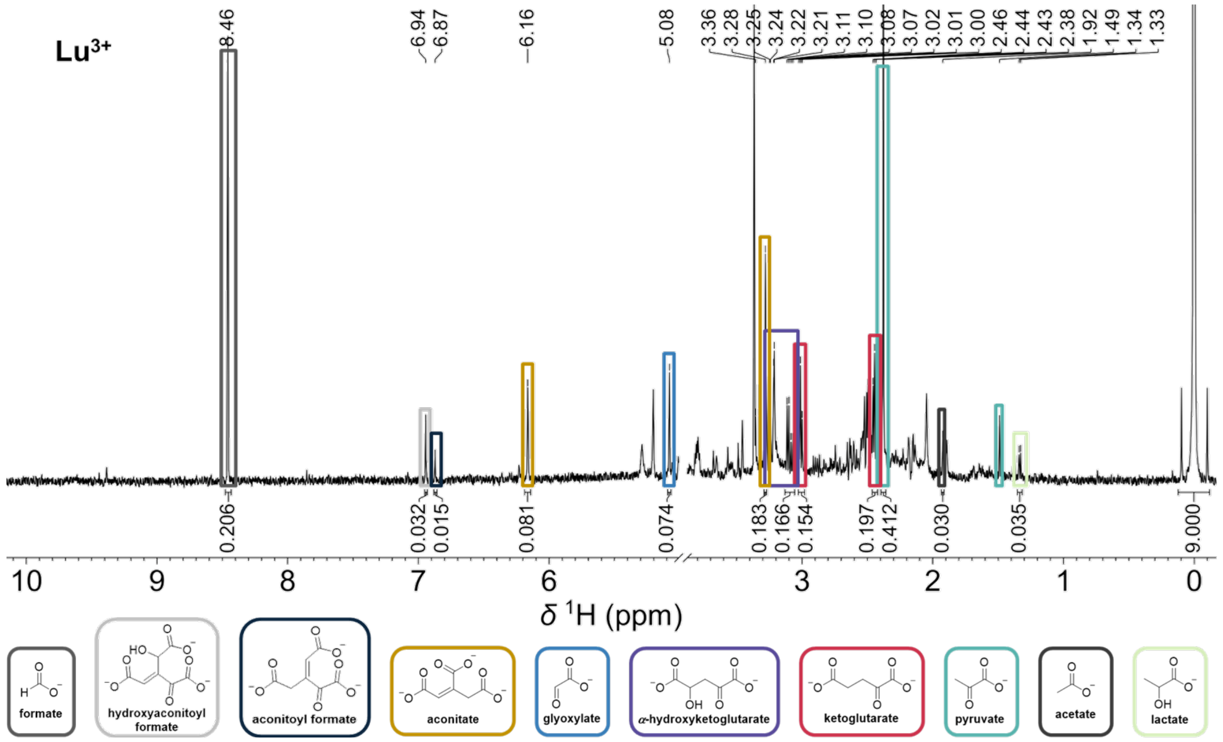

Figure S10. ^{1}H NMR spectrum with integral values of the reaction mixture containing $ScCl_3$ after 3 h at 70 °C with the assignable products marked in boxes and the corresponding structures marked with the same color below.

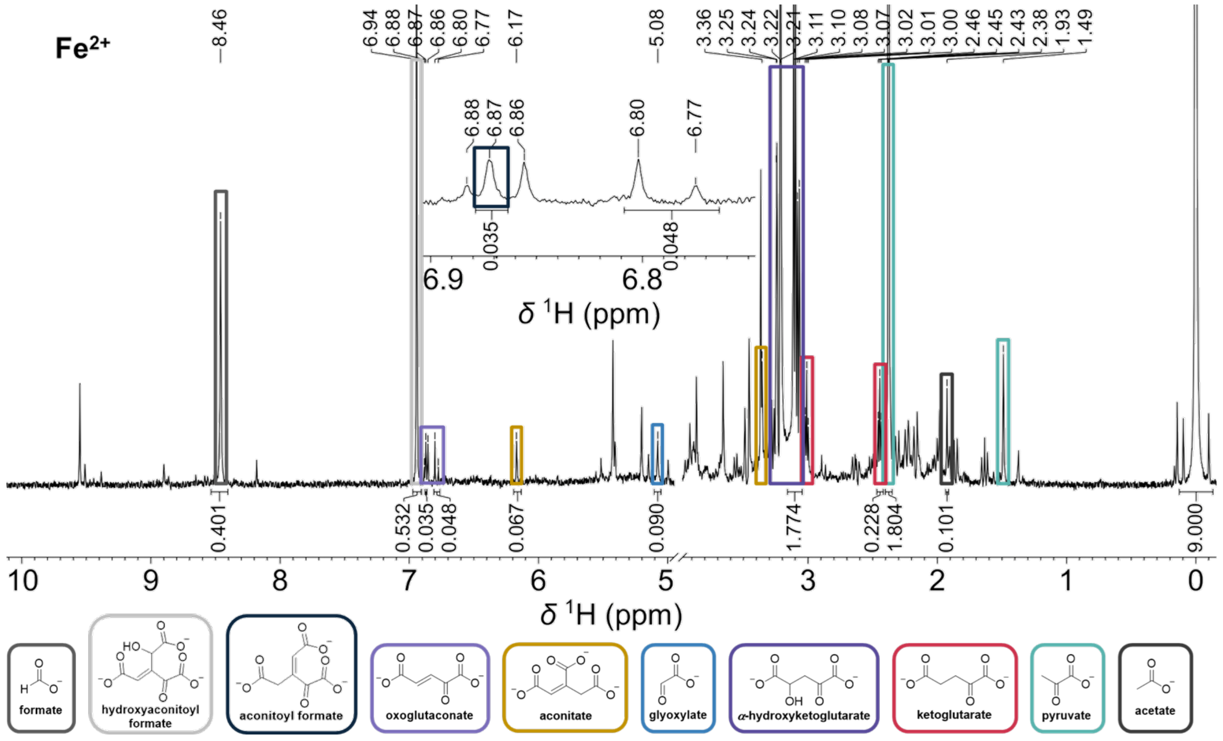

Figure S11. ^{1}H NMR spectrum with integral values of the reaction mixture containing $FeCl_2$ after 3 h at 70 °C with the assignable products marked in boxes and the corresponding structures marked with the same color below.

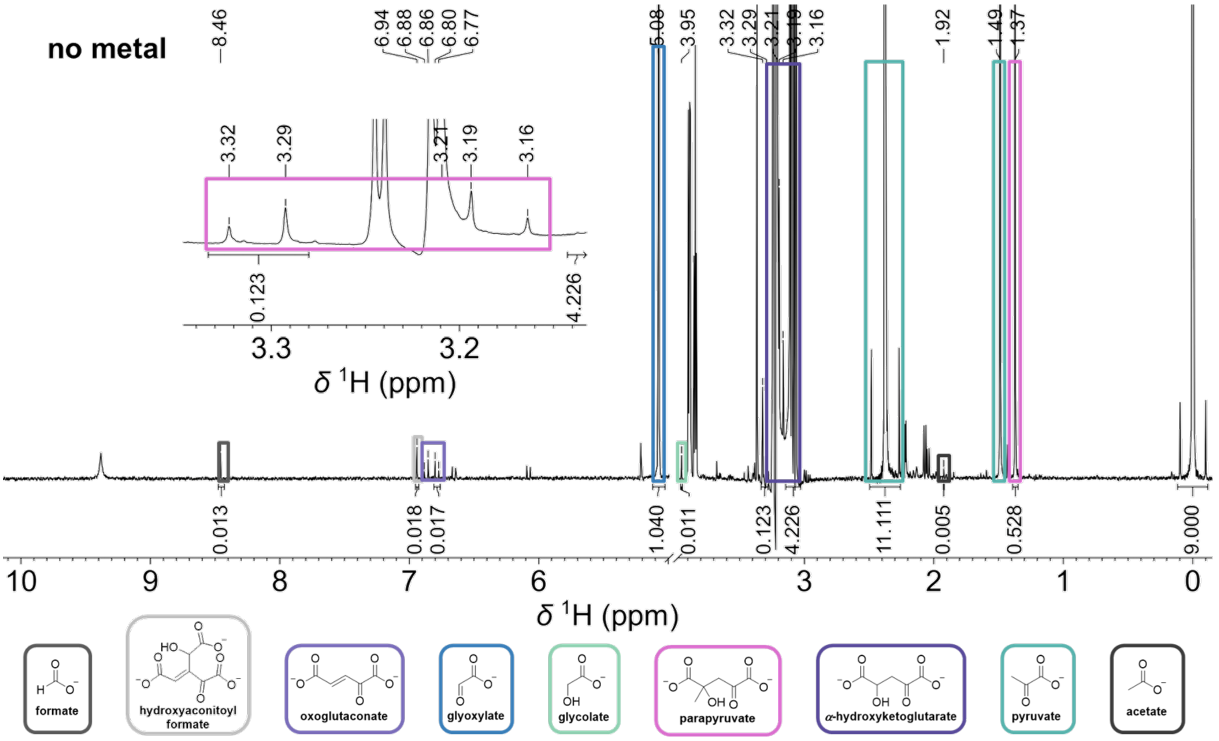

Figure S12. ^{1}H NMR spectrum with integral values of the reaction mixture containing no additional metal after 3 h at 70 °C with the assignable products marked in boxes and the corresponding structures marked with the same color below.

Table S2. Concentrations (mM) according to ^{1}H NMR of products observed in the reaction mixtures containing the various metal chlorides and no additional metal after 3 h at 70 °C.

**product**	*formate*	*acetate*	*glyoxylate*	*glycolate*	*pyruvate*	*lactate*
**metal salt**	NMR concentrations (mM)					
$ScCl_3$	0.05	0.02	0.34	2.57	0.03	0.27
YCl_3	0.72	0.02	0.13	0.08	0.41	0.02
$LaCl_3$	0.21	0.01	0.16	0.13	0.58	0.03
$CeCl_3$	0.30	0.02	0.11	0.15	0.76	0.03
$PrCl_3$	0.21	0.02	0.13	n.d.	0.80	0.02
$NdCl_3$	0.42	0.01	0.11	0.16	0.97	0.03
$EuCl_3$	0.80	0.09	n.d.	n.d.	0.64	0.04
$HoCl_3$	0.78	0.02	n.d.	n.d.	0.37	0
$LuCl_3$	0.57	0.03	0.21	n.d.	0.38	0.03
$FeCl_2$	1.11	0.09	0.25	n.d.	1.67	0
no metal	0.04	0.01	2.80	0.02	10.29	0
**product**	*oxoglutaconate*	*ketoglutarate*	*hkg*	*aconitate*	*aconityl formate*	*haf*
**metal salt**	NMR concentrations (mM)					
$ScCl_3$	0	0.05	0.28	0	0	0.04
YCl_3	0	0.35	0.49	0.19	0.05	0.06
$LaCl_3$	0.04	0.09	1.09	0	0.01	0.18
$CeCl_3$	0.02	0.11	0.72	0	0	0.09
$PrCl_3$	0.02	0.10	0.48	0	0	0.05
$NdCl_3$	0	0.19	0.38	0	0	0.03
$EuCl_3$	0	0.22	n.d.	0	0	0
$HoCl_3$	0	0.29	n.d.	0	0	0
$LuCl_3$	0	0.21	0.46	0.23	0.04	0.09
$FeCl_2$	0.13	0.32	4.93	0.19	0.10	1.48
no metal	0.05	0	11.74	0	0	0.05

Comparison of $FeSO_4$ and $La_2(SO_4)_3$ reactivity (time-resolved)

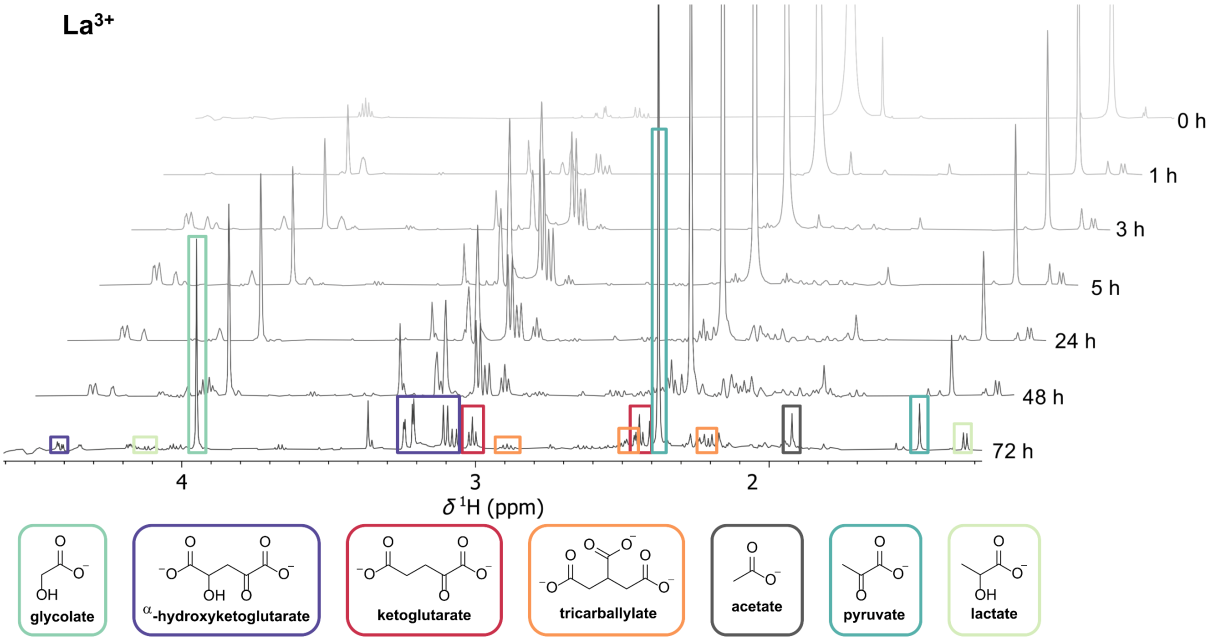

Figure S13. Time-resolved ^1H NMR spectra (higher field) of the reaction mixture in the La^{3+} reaction with the assignable products marked in boxes and the corresponding structures marked with the same color below.

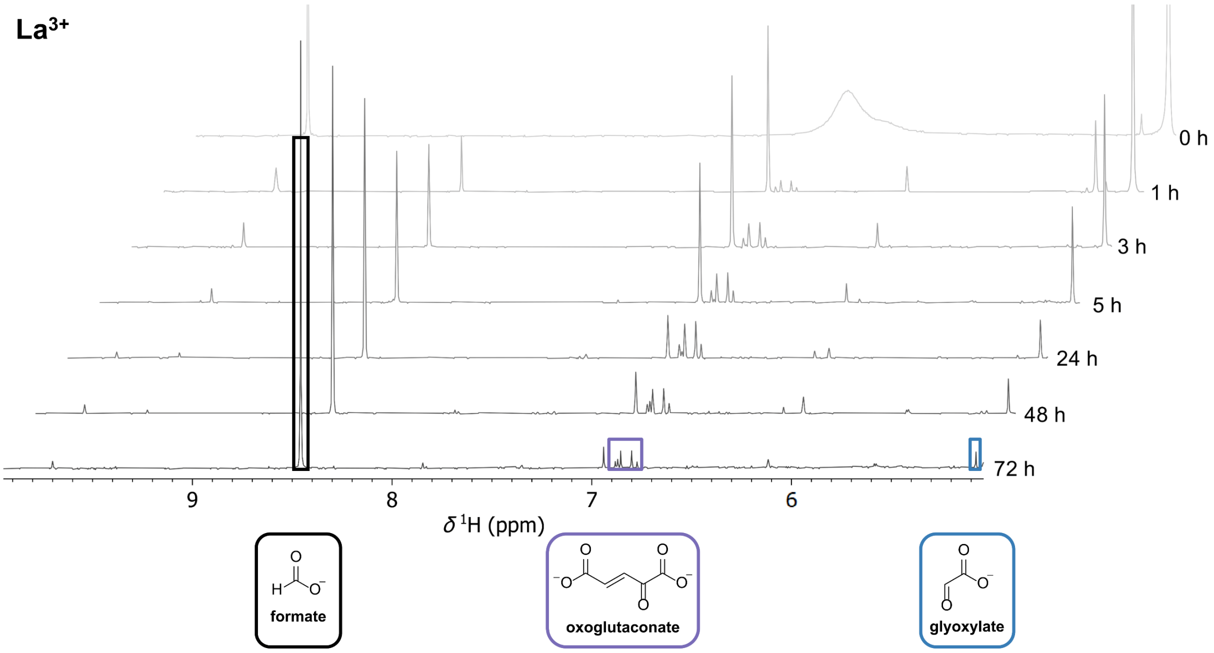

Figure S14. Time-resolved ^1H NMR spectra (higher field) of the reaction mixture in the La^{3+} reaction with the assignable products marked in boxes and the corresponding structures marked with the same color below.

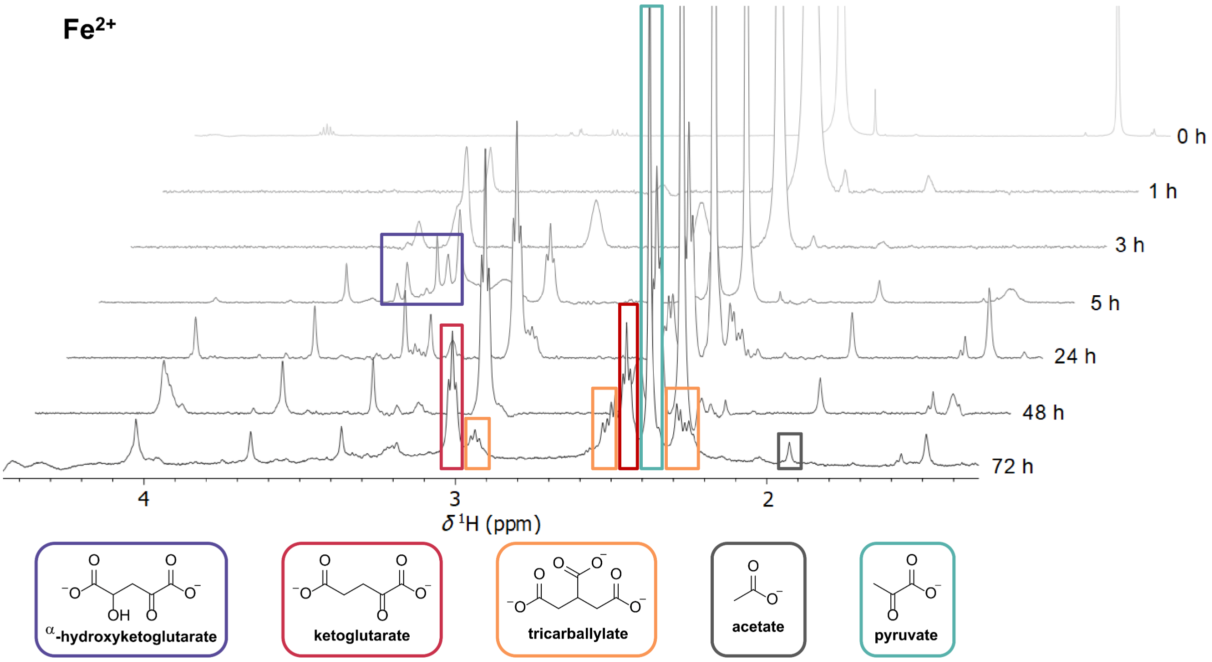

Figure S15. Time-resolved ^{1}H NMR spectra (higher field) of the reaction mixture in the Fe^{2+} reaction with the assignable products marked in boxes and the corresponding structures marked with the same color below.

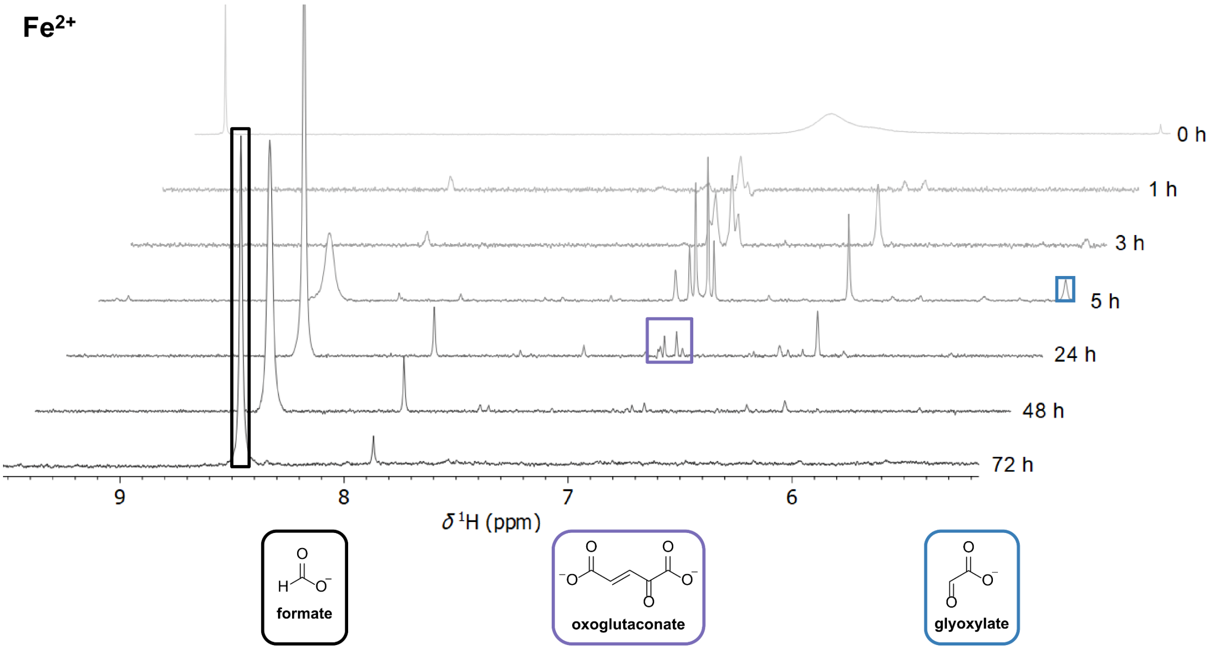

Figure S16. Time-resolved ^{1}H NMR spectra (lower field) of the reaction mixture in the Fe^{2+} reaction with the assignable products marked in boxes and the corresponding structures marked with the same color below.

Reaction with varying equivalents of La^{3+} for 3 h

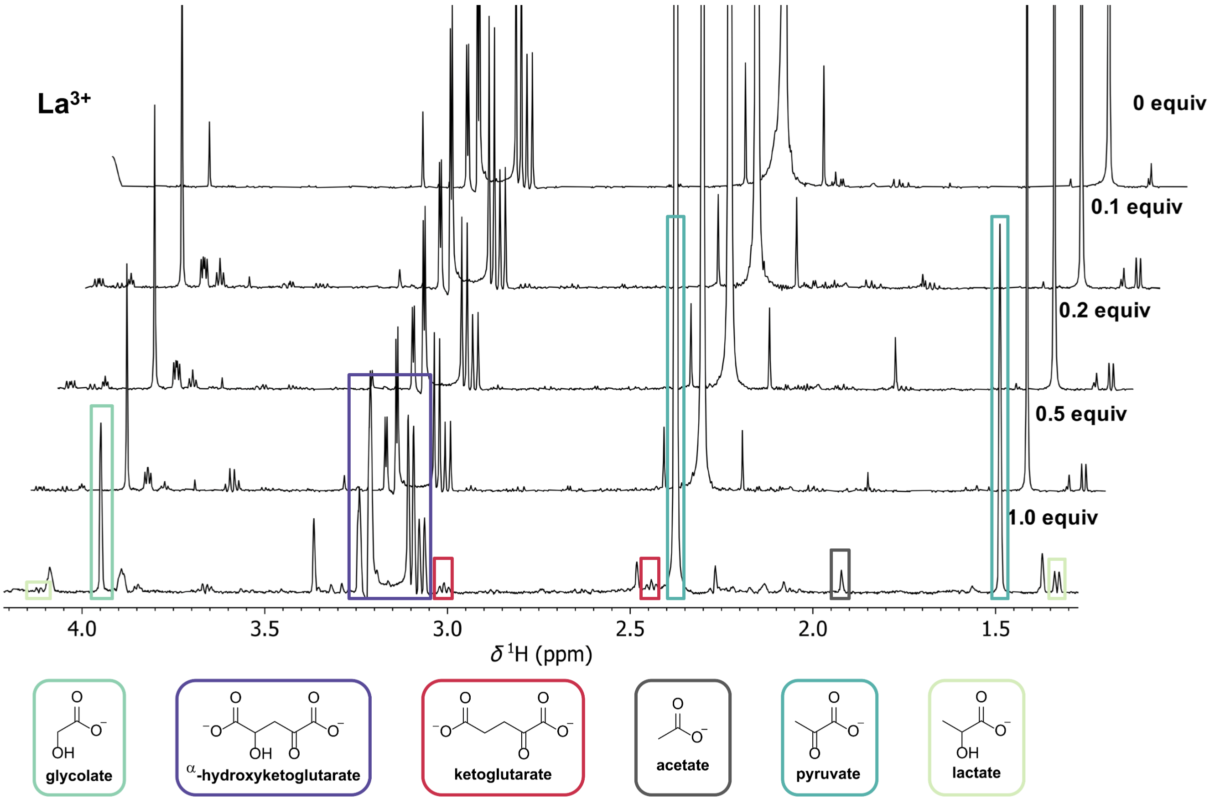

Figure S17. ¹H NMR spectra (higher field) of the reaction mixtures containing different amounts of La^{3+} after 3 h with the assignable products marked in boxes and the corresponding structures marked with the same color below.

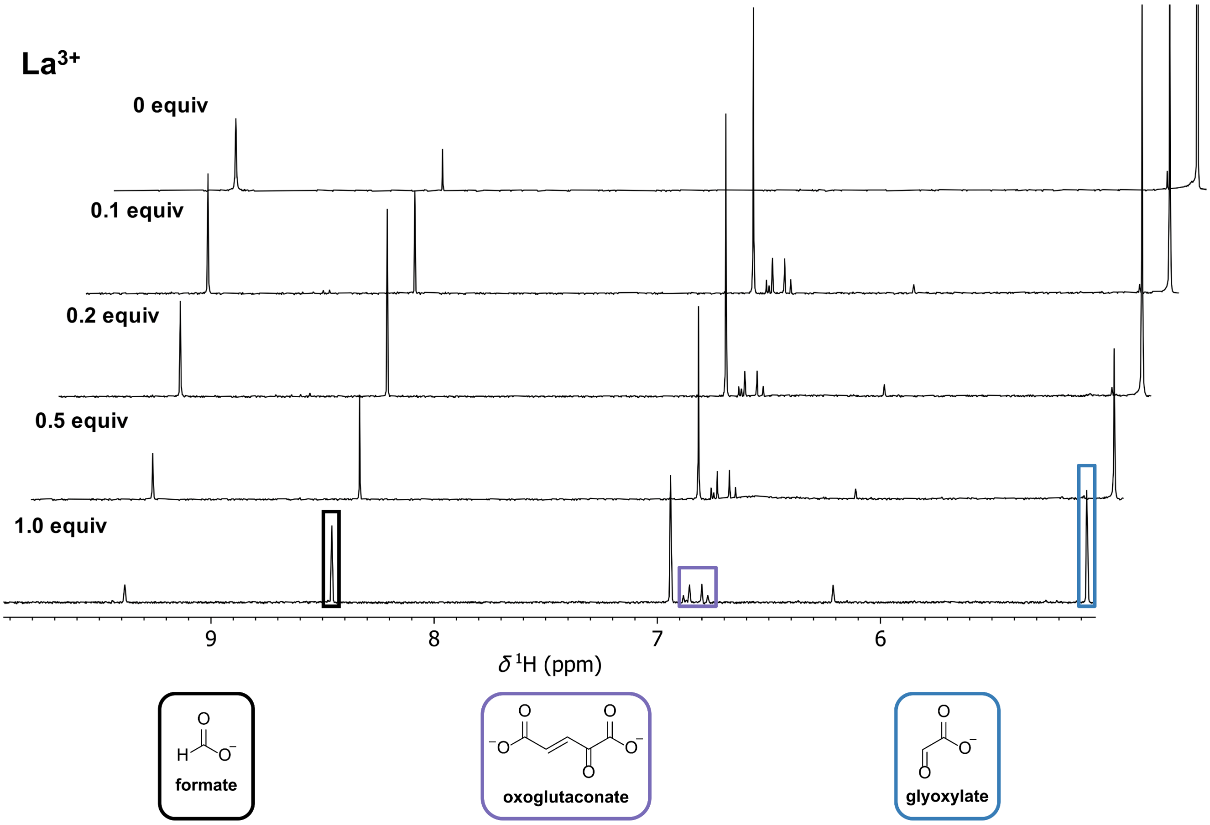

Figure S18. ¹H NMR spectra (lower field) of the reaction mixtures containing different amounts of La^{3+} after 3 h with the assignable products marked in boxes and the corresponding structures marked with the same color below.

Test of increased amount Chelex during workup of 72 h reaction

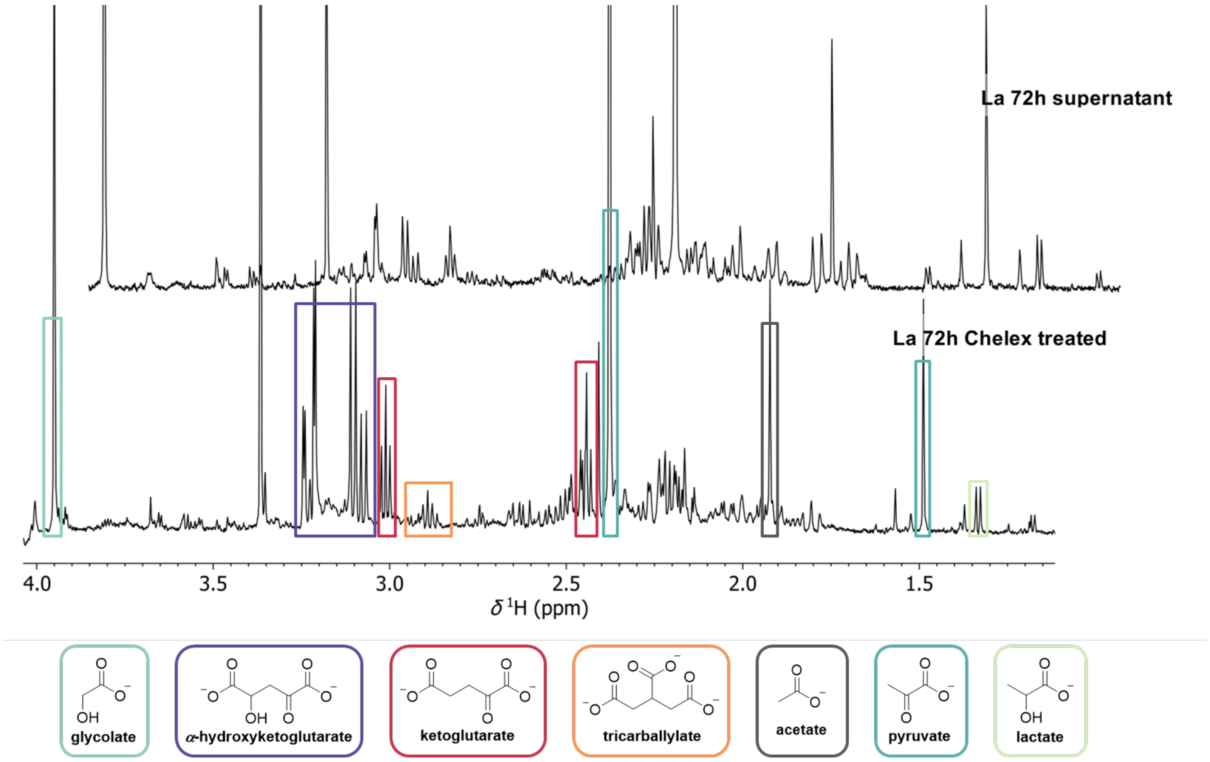

Figure S19. ¹H NMR spectra (higher field) of the supernatant (top) and the Chelex-treated resuspended reaction mixture containing $La_2(SO_4)_3$ after 72 h (bottom). The assignable products are marked in boxes and the corresponding structures are marked with the same color below.

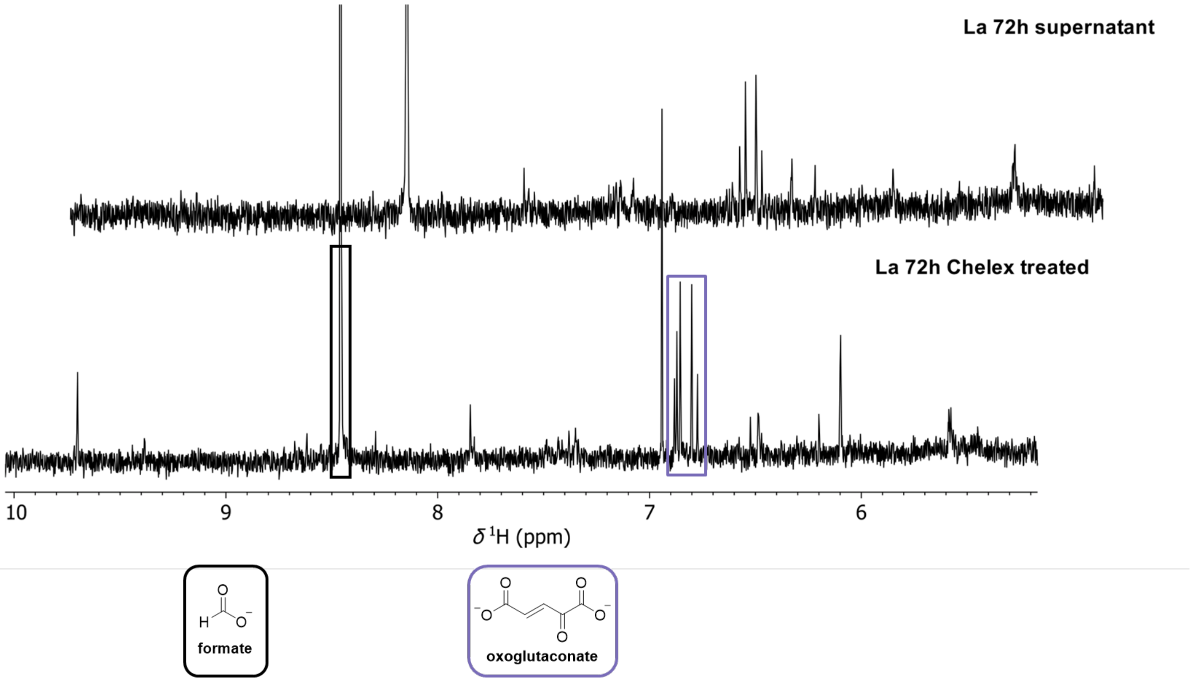

Figure S20. ¹H NMR spectra (lower field) of the supernatant (top) and the Chelex-treated (increased amount) resuspended reaction mixture containing $La_2(SO_4)_3$ after 72 h (bottom). The assignable products are marked in boxes and the corresponding structures are marked with the same color below.

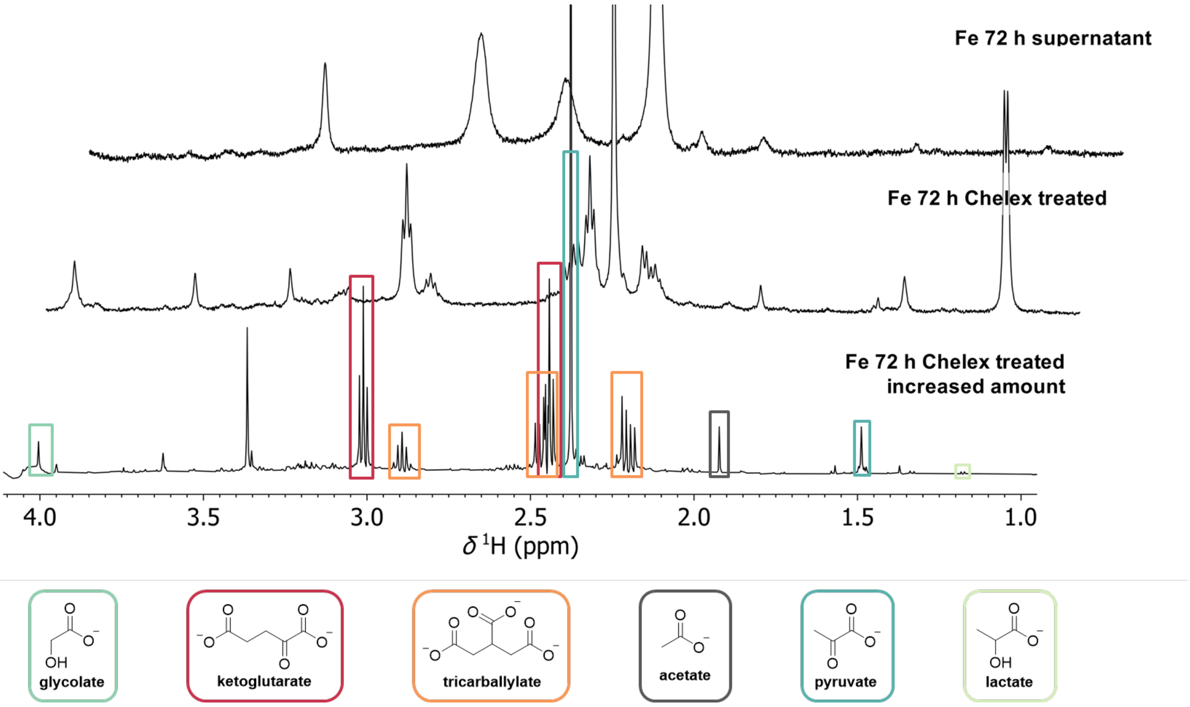

Figure S21. ^{1}H NMR spectra (higher field) of the supernatant (top) and the Chelex-treated (recommended amount middle, increased amount bottom) resuspended reaction mixture containing $FeSO_4$ after 72 h. The assignable products are marked in boxes and the corresponding structures are marked with the same color below.

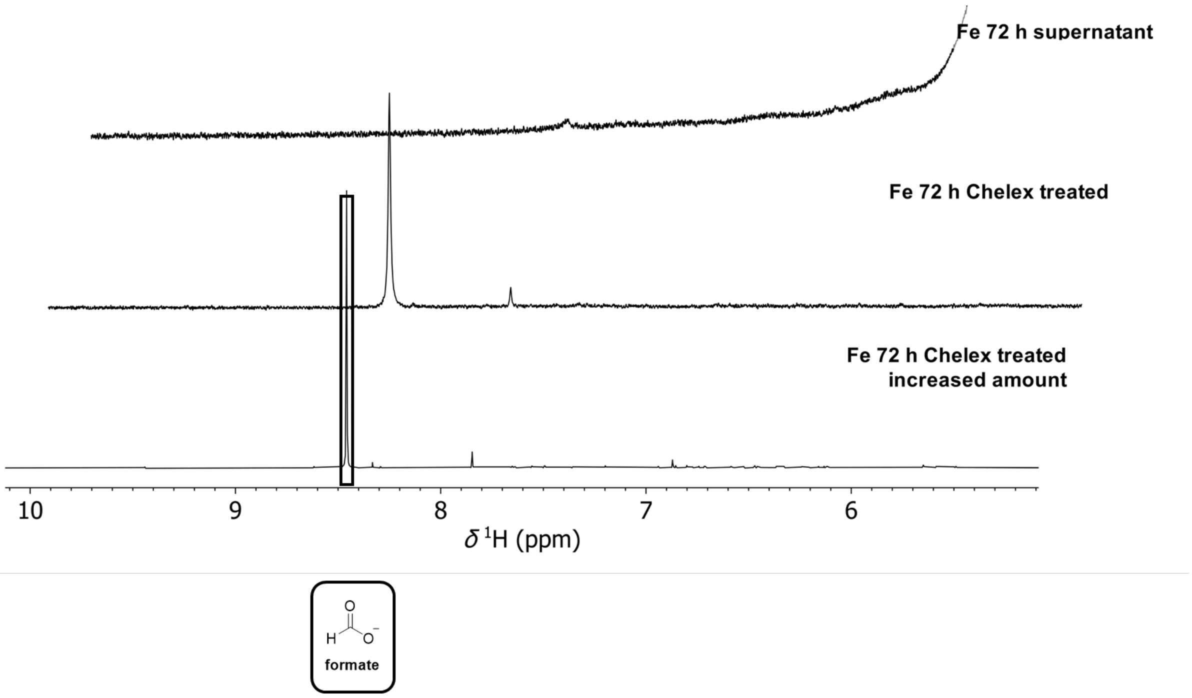

Figure S22. ^{1}H NMR spectra (lower field) of the supernatant (top) and the Chelex-treated (recommended amount middle, increased amount bottom) resuspended reaction mixture containing $FeSO_4$ after 72 h. The assignable products are marked in boxes and the corresponding structures are marked with the same color below.

Reaction with 10% La^{3+}, the precipitate or no metal addition for 72 h

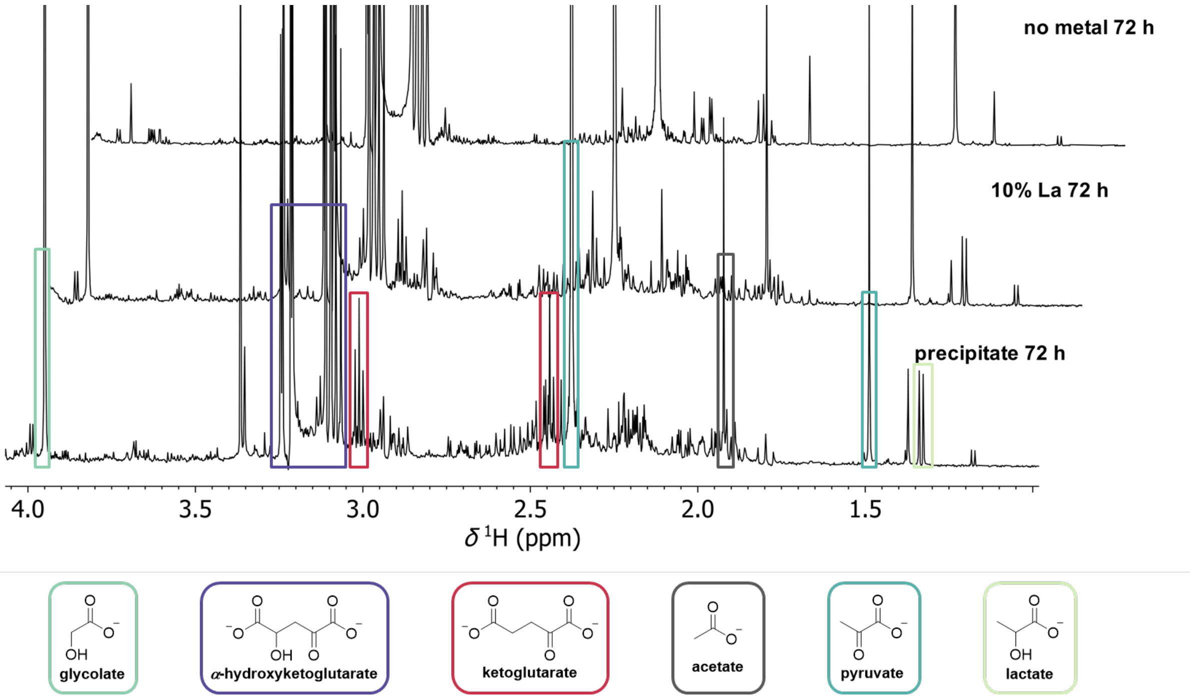

Figure S23. ^{1}H NMR spectra (higher field) of the reaction mixtures containing no added metal (top), 0.1 equiv of La^{3+} (middle) and the precipitate from a previous run (bottom). The assignable products are marked in boxes and the corresponding structures are marked with the same color below.

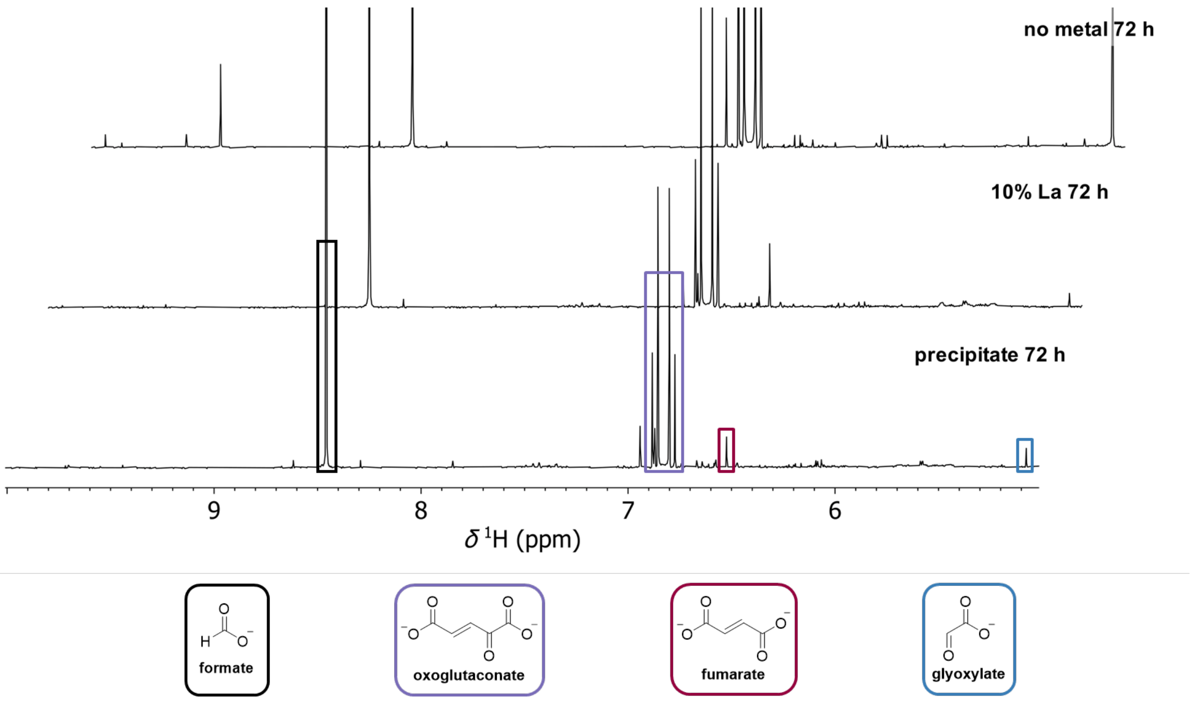

Figure S24. ^{1}H NMR spectra (lower field) of the reaction mixtures containing no added metal (top), 0.1 equiv of La^{3+} (middle) and the precipitate from a previous run (bottom). The assignable products are marked in boxes and the corresponding structures are marked with the same color below.

NMR Quantification after 72 h

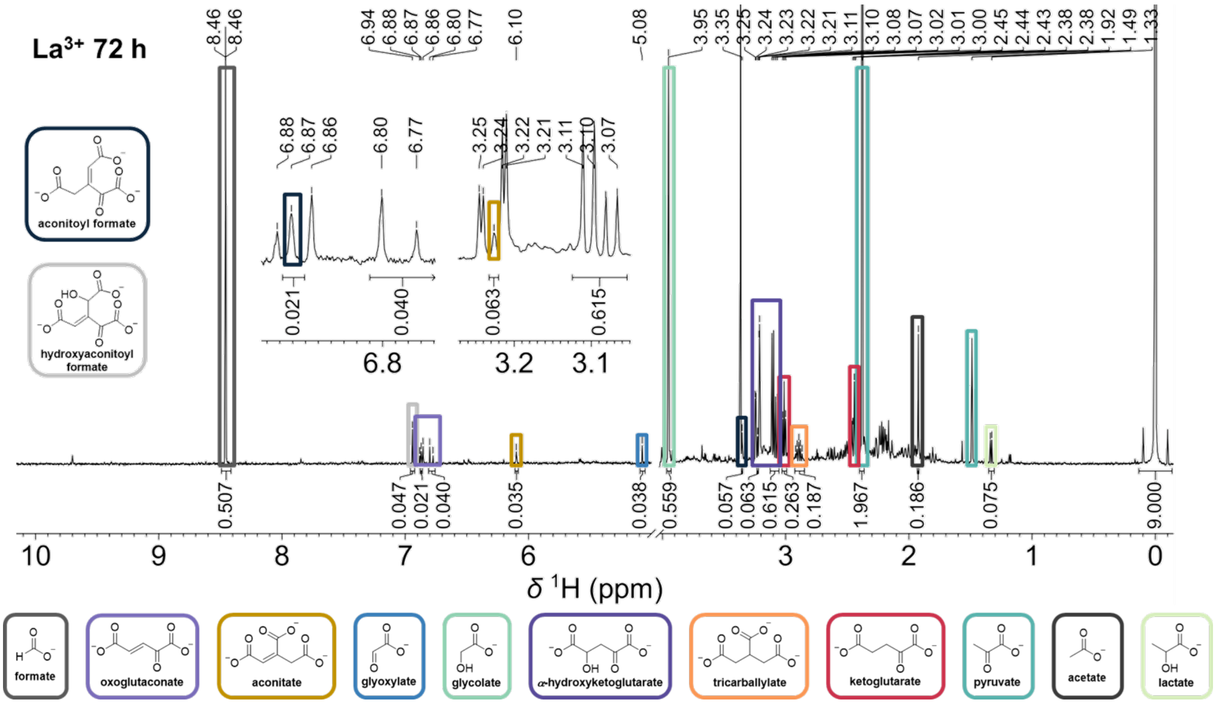

Figure S25. ^{1}H NMR spectrum with integral values of the reaction mixture containing an equimolar amount of $La_2(SO_4)_3$ after 72 h at 70 °C with the assignable products marked in boxes and the corresponding structures marked with the same color below.

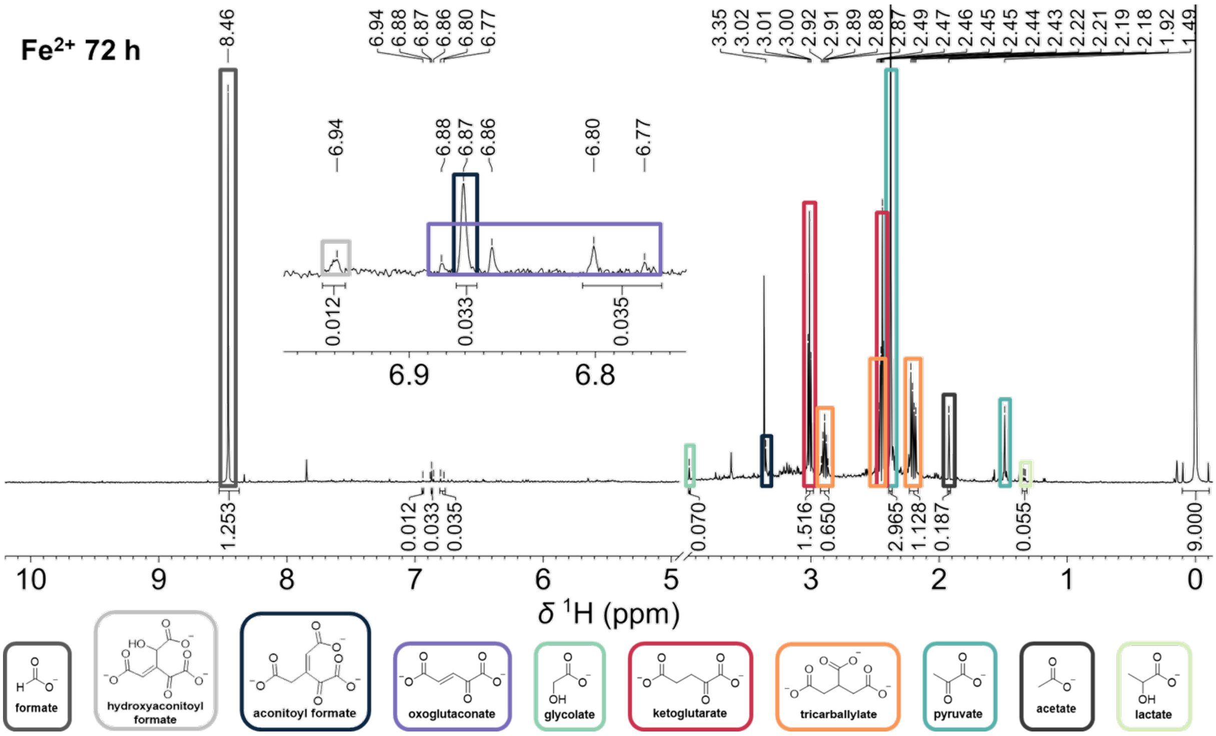

Figure S26. ^{1}H NMR spectrum with integral values of the reaction mixture containing an equimolar amount of $FeSO_4$ after 72 h at 70 °C with the assignable products marked in boxes and the corresponding structures marked with the same color below.

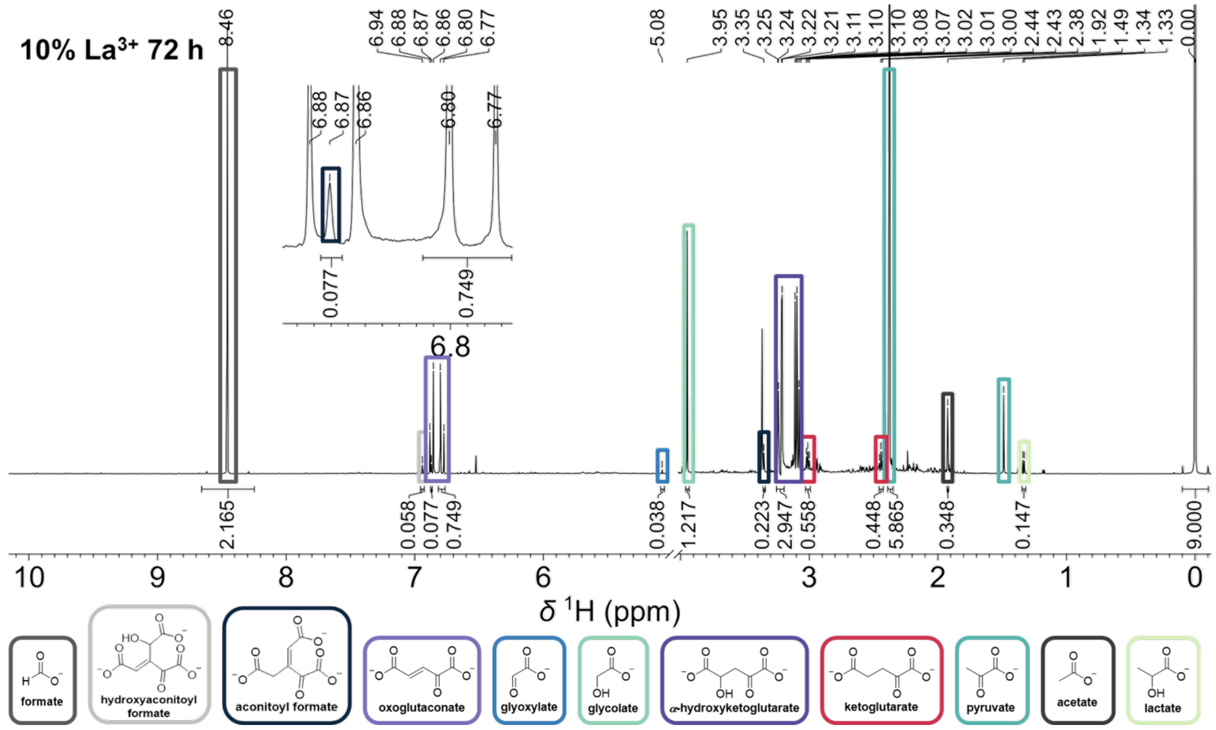

Figure S27. ^{1}H NMR spectrum with integral values of the reaction mixture containing 0.1 equiv of $La_2(SO_4)_3$ after 72 h at 70 °C with the assignable products marked in boxes and the corresponding structures marked with the same color below.

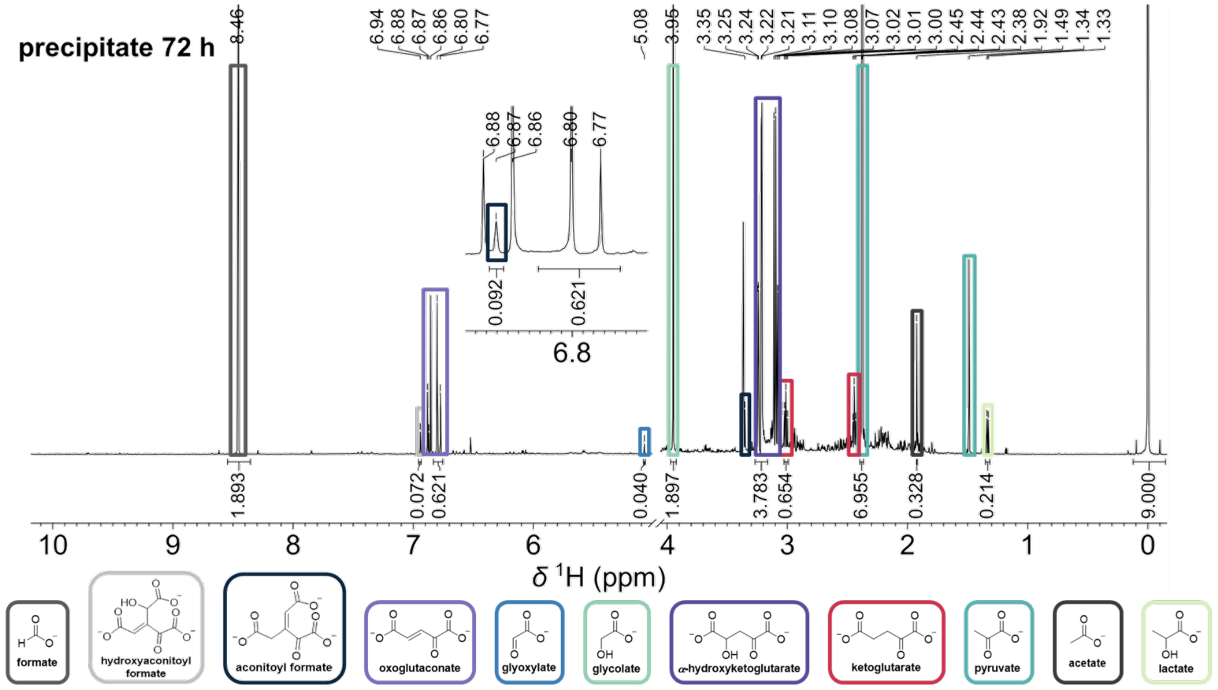

Figure S28. ^{1}H NMR spectrum with integral values of the reaction mixture containing an equimolar amount of La^{3+} in the form of the precipitate from a previous run after 72 h at 70 °C with the assignable products marked in boxes and the corresponding structures marked with the same color below.

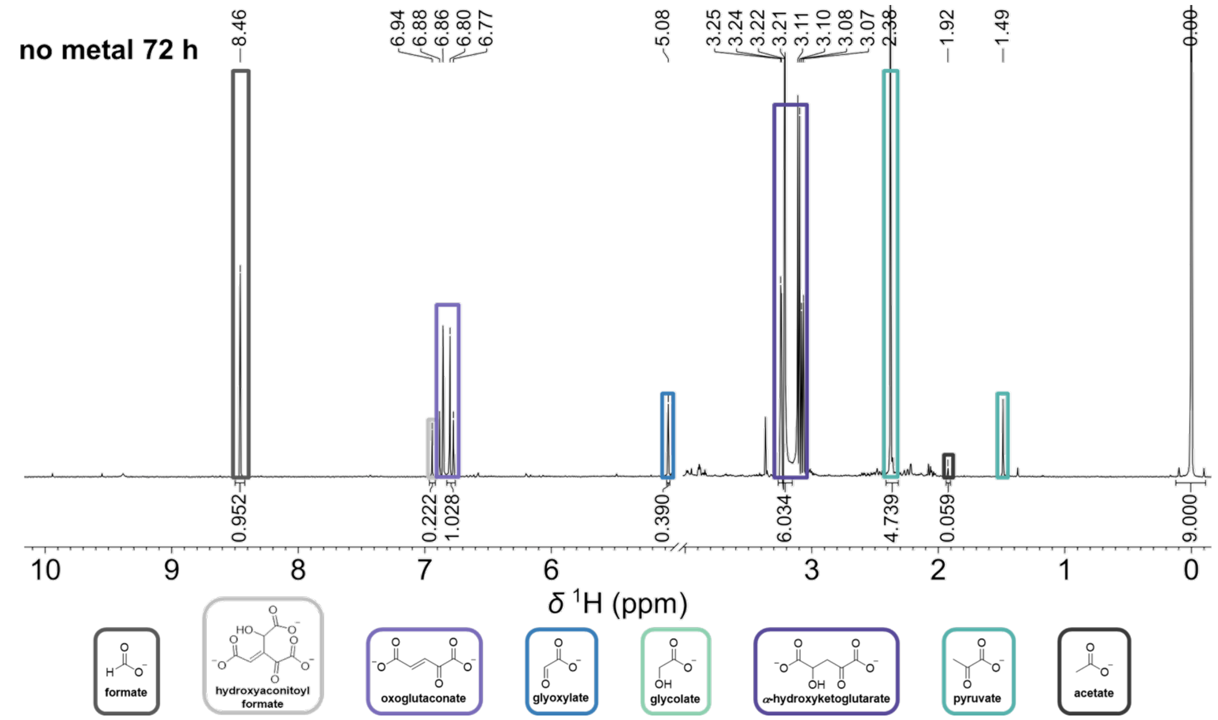

Figure S29. ^{1}H NMR spectrum with integral values of the reaction mixture containing no additional metal after 72 h at 70 °C with the assignable products marked in boxes and the corresponding structures marked with the same color below.

Table S3. Concentrations (mM) according to ^{1}H NMR of products observed in the reaction mixtures containing the various metal chlorides and no additional metal after 3 h at 70 °C.

**product**	*formate*	*acetate*	*glyoxylate*	*glycolate*	*pyruvate*	*lactate*
**metal salt**	NMR concentrations (mM)					
La^{3+} 72 h	1.40	0.17	0.11	0.77	1.83	0.07
Fe 72 h	3.45	0.17	0	0.10	2.72	0.05
10% La^{3+} 72 h	5.97	0.32	0.11	1.68	5.39	0.14
precipitate 72 h	5.22	0.30	0.11	2.61	6.39	0.20
no metal 72 h	2.62	0.05	1.08	0	4.35	0

**product**	*oxoglutaconate*	*ketoglutarate*	*hkg*	*aconitate*	*tricarballylate*	*aconityl formate*	*haf*
**metal salt**	NMR concentrations (mM)						
La^{3+} 72 h	0.11	0.36	1.70	0.10	0.52	0.06	0.13
Fe 72 h	0.10	2.09	4.18	0	1.79	0.09	0.03
10% La^{3+} 72 h	2.06	0.77	8.12	0	0	0.21	0.16
precipitate 72 h	1.71	0.90	10.42	0	0	0.25	0.20
no metal 72 h	2.83	0	16.63	0	0	0	0.62

Reaction of La^{3+} with glyoxylate or pyruvate only for 3 h

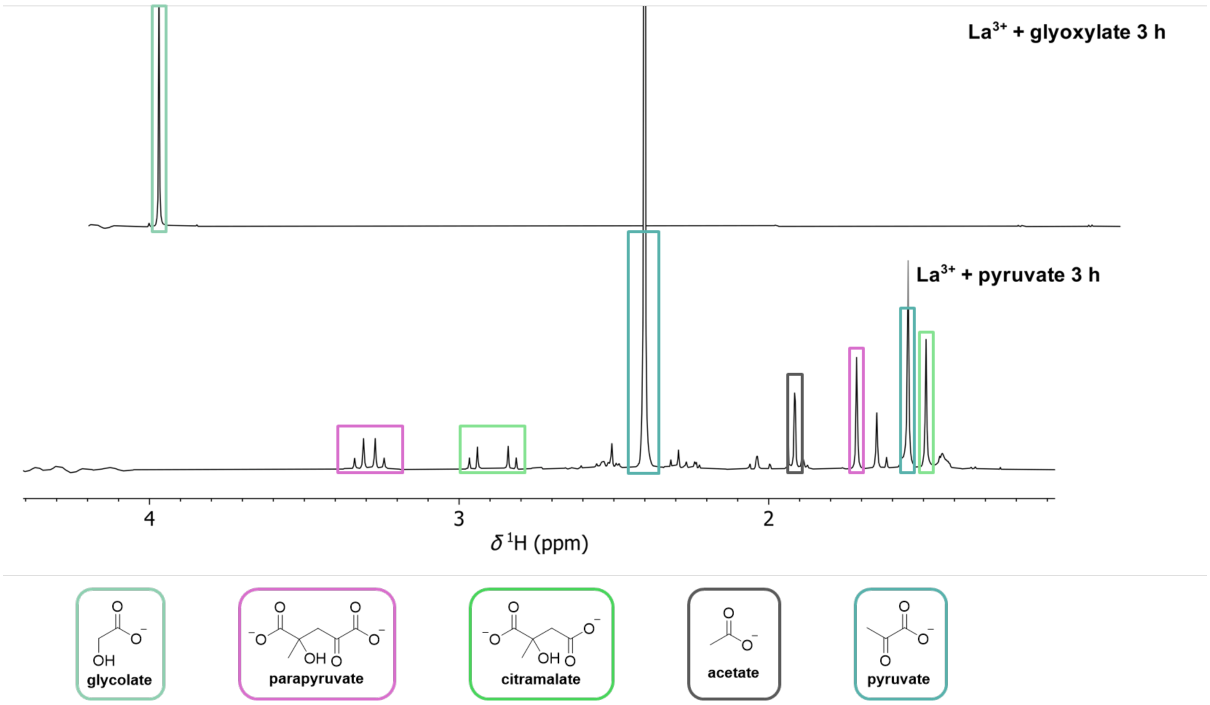

Figure S30. ^{1}H NMR spectra (higher field) of the reaction mixtures containing only $La_2(SO_4)_3$ and glyoxylate (top) and analogous containing only $La_2(SO_4)_3$ and pyruvate (bottom). The assignable products are marked in boxes and the corresponding structures are marked with the same color below.

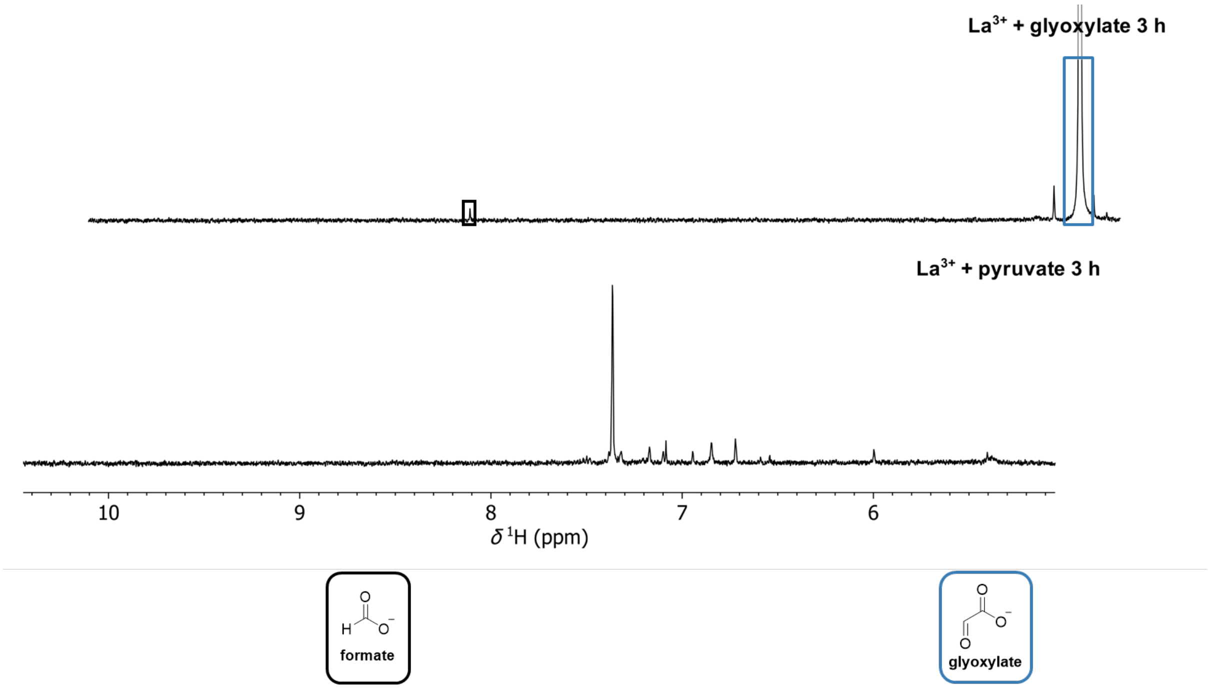

Figure S31. ^{1}H NMR spectra (lower field) of the reaction mixtures containing only $La_2(SO_4)_3$ and glyoxylate (top) and analogous containing only $La_2(SO_4)_3$ and pyruvate (bottom). The assignable products are marked in boxes and the corresponding structures are marked with the same color below.

GC-MS Data

REE Chlorides vs. $FeCl_2$ vs. no Metal

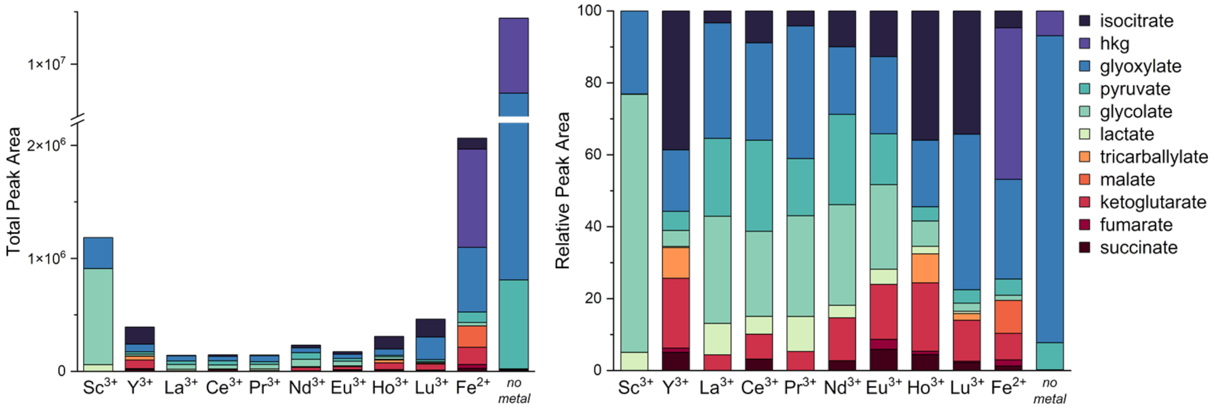

Figure S32. Absolute and relative peak areas of reaction product gas chromatograms of glyoxlate and pyruvate in the presence of different REE chlorides, ferrous chloride and no metal ions after 3 h at 70 °C (hkg = α-hydroxyketoglutarate).

Table S4. Absolute peak areas of the assignable products in GC-MS chromatograms of the reactions containing the various REE chlorides, $FeCl_2$ and no metal for comparison after 3 h at 70 °C (hkg = α-hydroxyketoglutarate).

**product**	*pyruvate*	*glycolate*	*lactate*	*succinate*	*fumarate*	*glyoxylate*
RT	4.981	5.287	5.412	5.474	5.521	6.33
**metal salt**						
$ScCl_3$	1502.32	849613.89	59810.27	0	0	273063.69
YCl_3	20827.75	17446.98	1248.50	19844.10	4721.22	67089.60
$LaCl_3$	30839.77	42414.58	12429.38	0	0	45700.98
$CeCl_3$	37049.36	34657.37	7243.87	4675.86	0	39623.28
$PrCl_3$	23017.08	40615.34	14100.51	0	0	53457.50
$NdCl_3$	58242.92	64951.50	8067.97	6351.84	0	43628.85
$EuCl_3$	24605.73	40996.01	7334.97	10305.08	4806.64	37354.14
$HoCl_3$	12254.66	21823.83	6491.53	13875.03	2748.80	57325.39
$LuCl_3$	17200.34	10523.26	2796.04	10298.51	1708.78	200064.01
$FeCl_2$	92616.54	30011.84	0	26520.60	34661.24	572472.74
no metal	787310.39	5427.80	0	8887.58	7957.10	8914172.80

**product**	*ketoglutarate*	*malate*	*tricarballylate*	*isocitrate*	*hkg*
RT	6.667	7.638	7.835	7.876	8.644
**metal salt**					
$ScCl_3$	0	0	0	0	0
YCl_3	76161.30	0	33489.15	151553.13	0
$LaCl_3$	6235.54	0	0	4709.47	0
$CeCl_3$	10113.51	0	0	12950.35	0
$PrCl_3$	7694.16	0	0	6013.26	0
$NdCl_3$	27693.26	0	0	23108.54	0
$EuCl_3$	26635.91	0	0	22128.47	0
$HoCl_3$	58956.68	0	24915.48	111253.09	0
$LuCl_3$	52759.99	0	8640.43	158490.93	0
$FeCl_2$	152503.41	188732.59	0	96210.96	870365.56
no metal	0	0	0	0	715827.96

Time-resolved Reaction with $La_2(SO_4)_3$ and $FeCl_2$

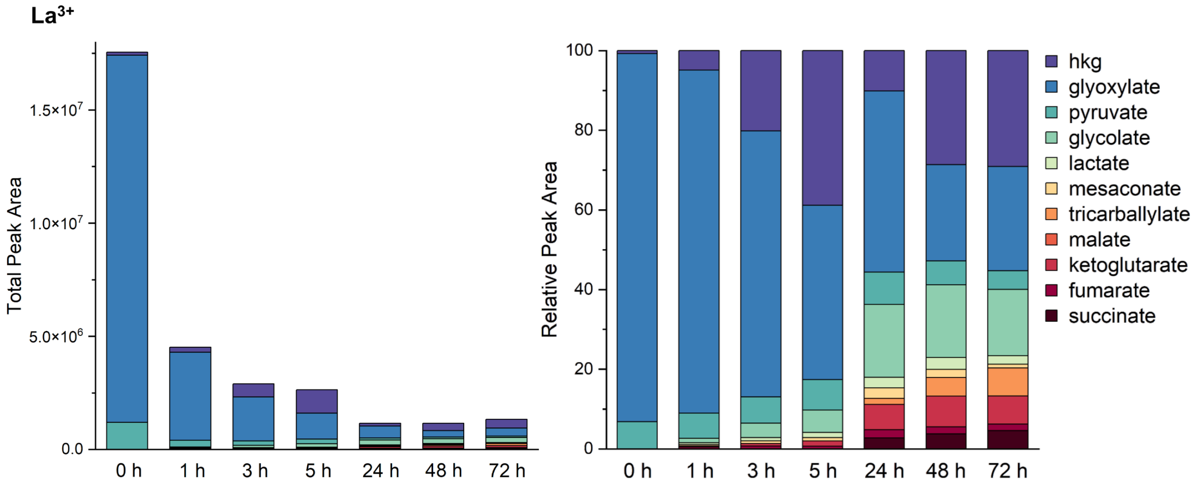

Figure S33. Total and relative peak areas of the detected products in samples from the reaction mixture in the presence of $La_2(SO_4)_3$ at different points in time. The first column (0 h) represents the starting materials before metal addition.

Table S5. Absolute peak areas of the assignable products in GC-MS chromatograms of the reaction containing $La_2(SO_4)_3$ after different points in time at 70 °C (hkg = *α*-hydroxyketoglutarate).

**product**	*pyruvate*	*glycolate*	*lactate*	*succinate*	*fumarate*	*glyoxylate*
RT	4.981	5.287	5.412	5.474	5.521	6.33
**time**						
0 h	1199329.00	0	0	0	0	16224909.00
1 h	287953.81	50053.38	21452.86	0	20977.95	3893655.30
3 h	192411.84	104788.05	23165.01	0	21693.78	1942365.70
5 h	202318.29	147781.55	34429.31	0	17772.91	1154813.54
24 h	94554.67	211631.85	30695.20	32013.86	23347.00	526896.86
48 h	70867.45	211970.54	35015.87	44333.32	19923.50	281714.22
72 h	62356.39	221518.51	28843.47	61199.16	21703.69	349177.88
**product**	*ketoglutarate*	*malate*	*tricarb-allylate*	*isocitrate*	*mesaconate*	*hkg*
RT	6.667	7.638	7.835	7.876	8.063	8.644
**time**						
0 h	0	0	0	0	0	126921.00
1 h	11367.59	0	0	0	15292.83	219412.32
3 h	17549.35	0	0	0	19949.26	585192.45
5 h	34127.52	0	0	0	22447.57	1022724.45
24 h	73717.10	0	17528.26	0	31038.48	117606.12
48 h	89670.49	0	55109.90	0	23875.12	334126.31
72 h	94085.25	0	93627.96	0	12708.44	387239.45

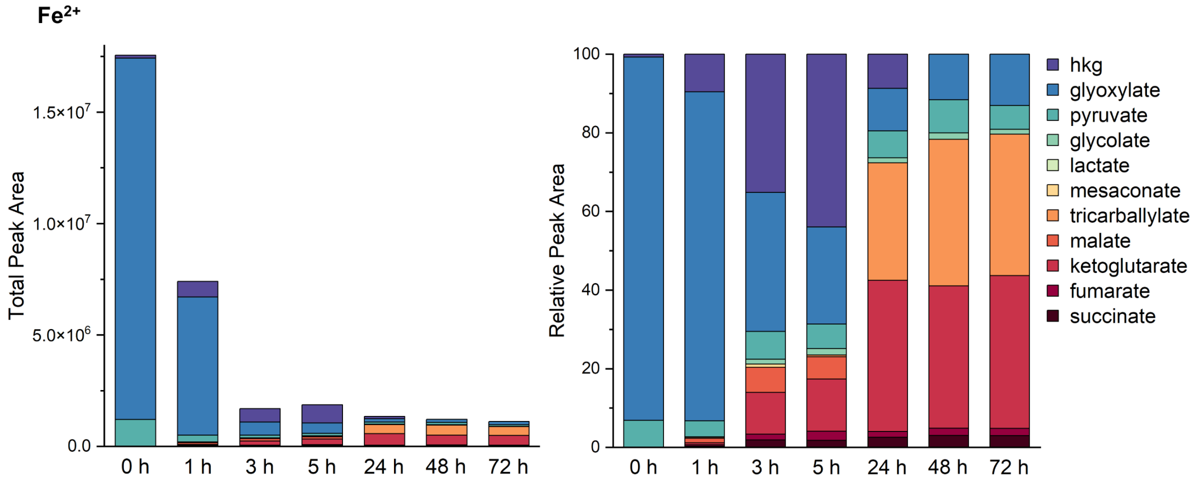

Figure S34. Total and relative peak areas of the detected products in samples from the reaction mixture in the presence of $FeSO_4$ at different points in time. The first column (0 h) represents the starting materials before metal addition. after certain periods of time.

Table S6. Absolute peak areas of the assignable products in GC-MS chromatograms of the reaction containing $FeSO_4$ after different points in time at 70 °C (hkg = *α*-hydroxyketoglutarate).

**product**	*pyruvate*	*glycolate*	*lactate*	*succinate*	*fumarate*	*glyoxylate*
RT	4.981	5.287	5.412	5.474	5.521	6.33
**time**						
0 h	1199329.00	0	0	0	0	16224909.00
1 h	301626.49	12281.46	0	21240.05	26578.37	6196823.23
3 h	119210.22	21252.11	0	31398.08	24816.78	597295.45
5 h	116439.92	31196.57	0	33499.57	42836.52	461302.90
24 h	92397.97	17042.37	0	34476.13	19958.26	145923.85
48 h	102691.25	19695.61	0	36884.01	22638.52	140576.46
72 h	67498.82	13713.65	0	33233.33	20446.45	145449.54

**product**	*keto-glutarate*	*malate*	*tricarb-allylate*	*isocitrate*	*mesaconate*	*hkg*
RT	6.667	7.638	7.835	7.876	8.063	8.644
**time**						
0 h	0	0	0	0	0	126921.00
1 h	36397.07	85572.53	0	0	17655.1	706840.61
3 h	179828.22	108519.98	0	0	12839.73	594169.25
5 h	248341.62	105686.92	7761.99	0	0	820096.02
24 h	519286.82	0	403791.17	0	0	117606.12
48 h	439504.74	0	453660.28	0	0	0
72 h	432858.19	0	401133.57	0	0	0

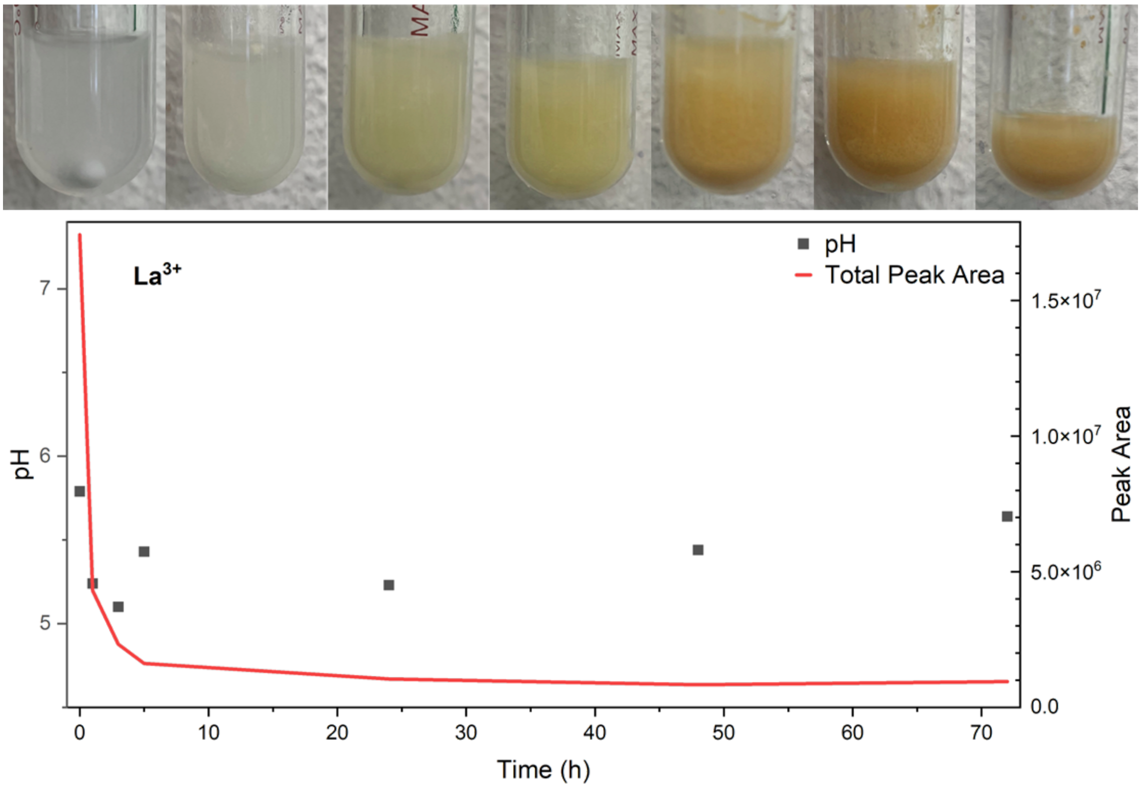

Figure S35. The course of pH and total GC-MS peak area over time for the La^{3+} reaction. Above are shown pictures of the reaction mixture from left to right, before metal addition (0 h), after 1 h, 3 h, 5 h, 24 h, 48 h, 72 h, respectively.

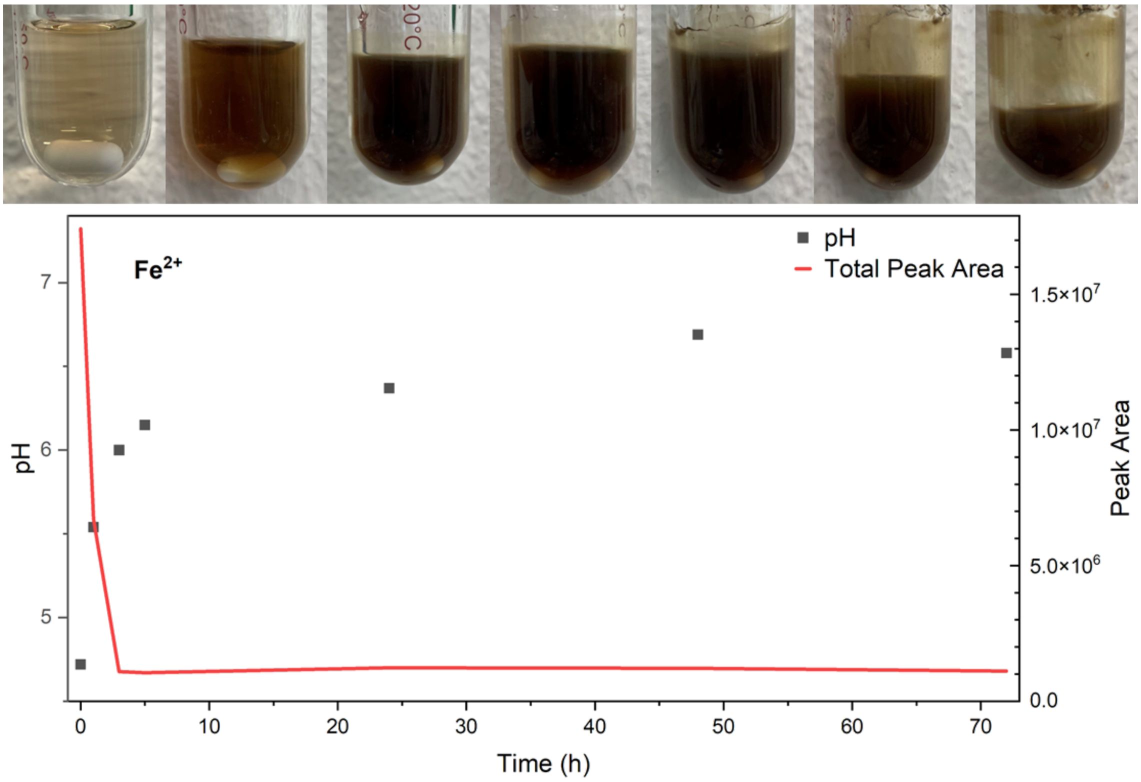

Figure S36. The course of pH and total GC-MS peak area over time for the Fe^{2+} reaction. Above are shown pictures of the reaction mixture from left to right, before metal addition (0 h), after 1 h, 3 h, 5 h, 24 h, 48 h, 72 h, respectively.

Gas Chromatograms

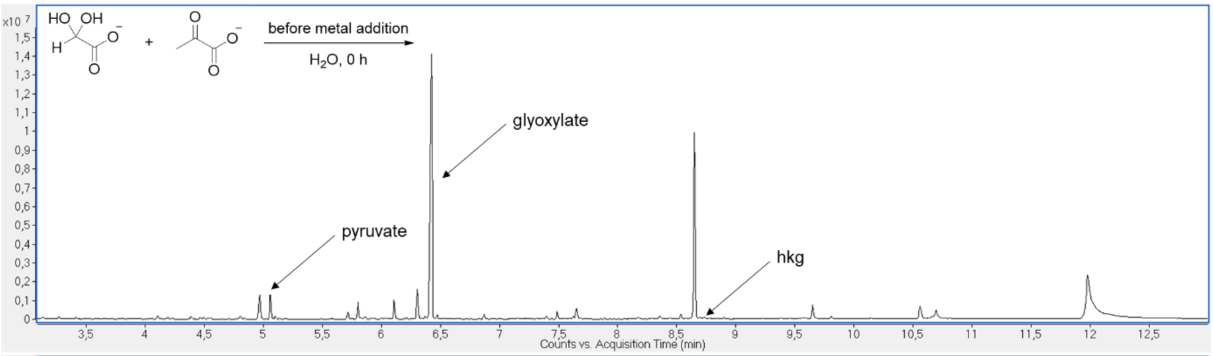

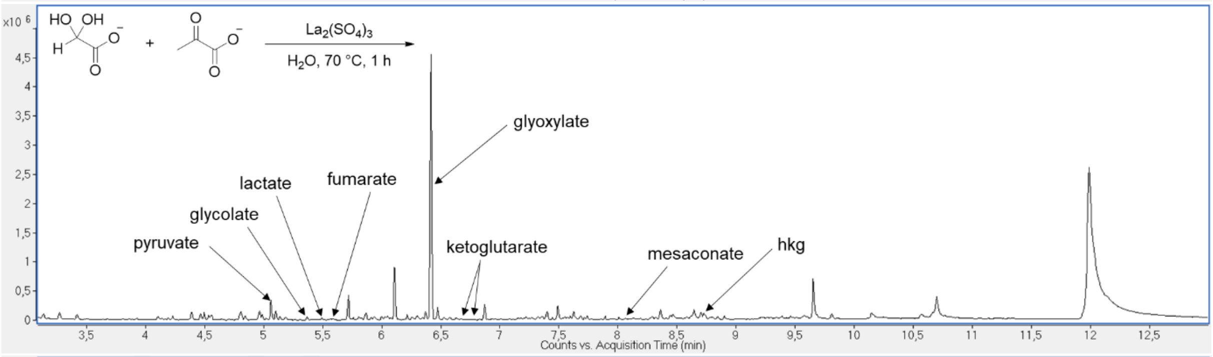

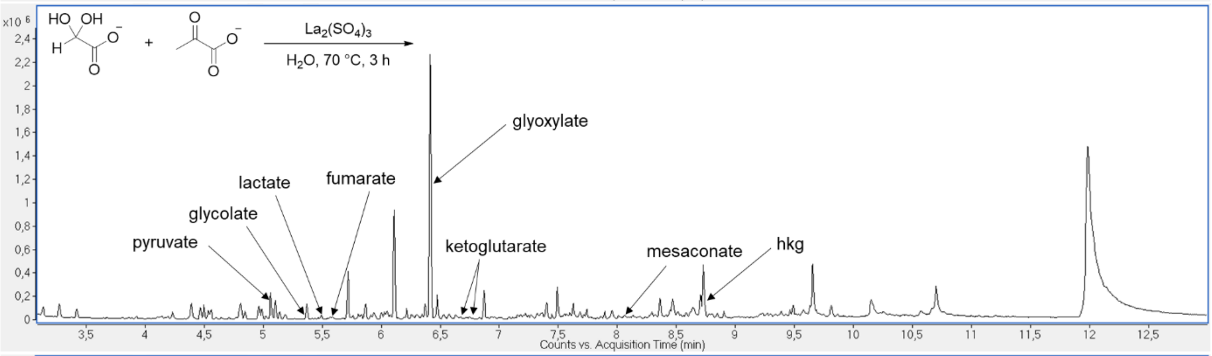

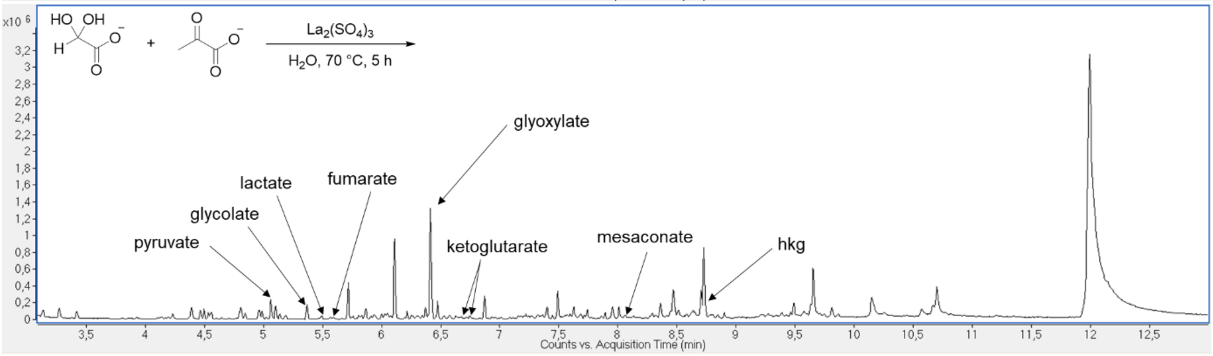

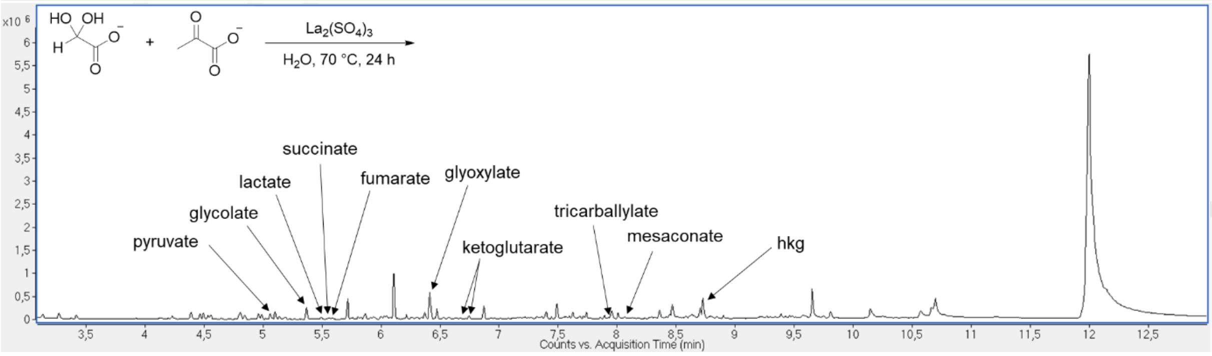

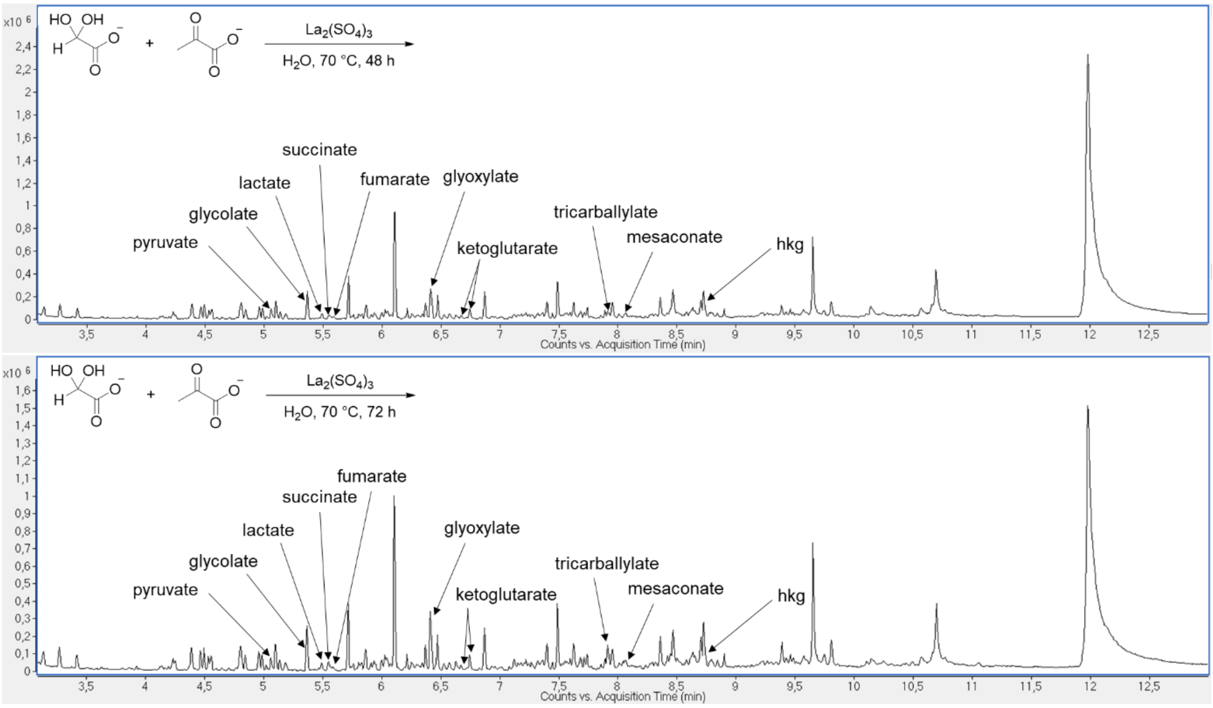

Figure S37. Gas chromatograms of derivatized samples taken from the reaction mixture containing $La_2(SO_4)_3$ at different points in time.

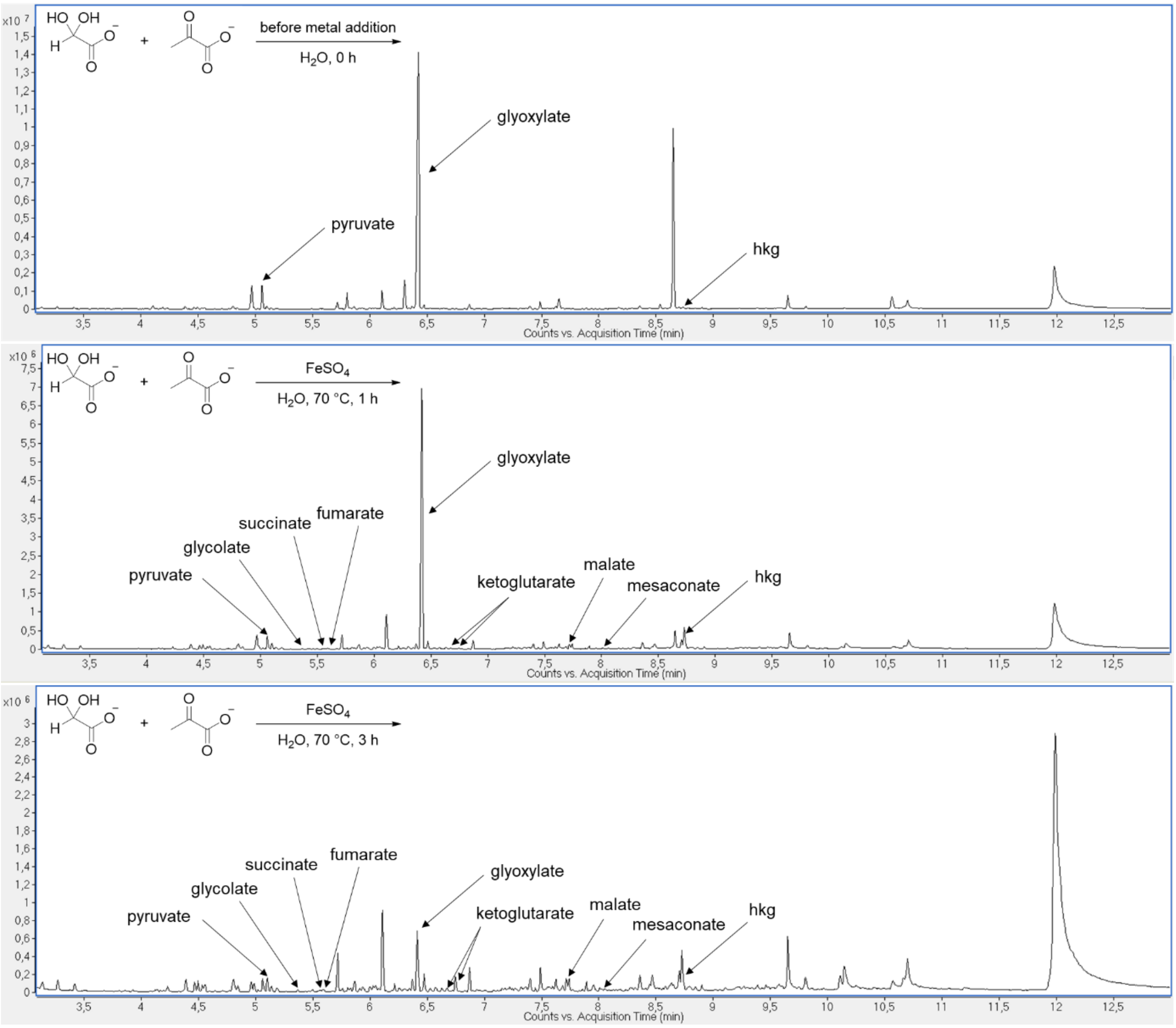

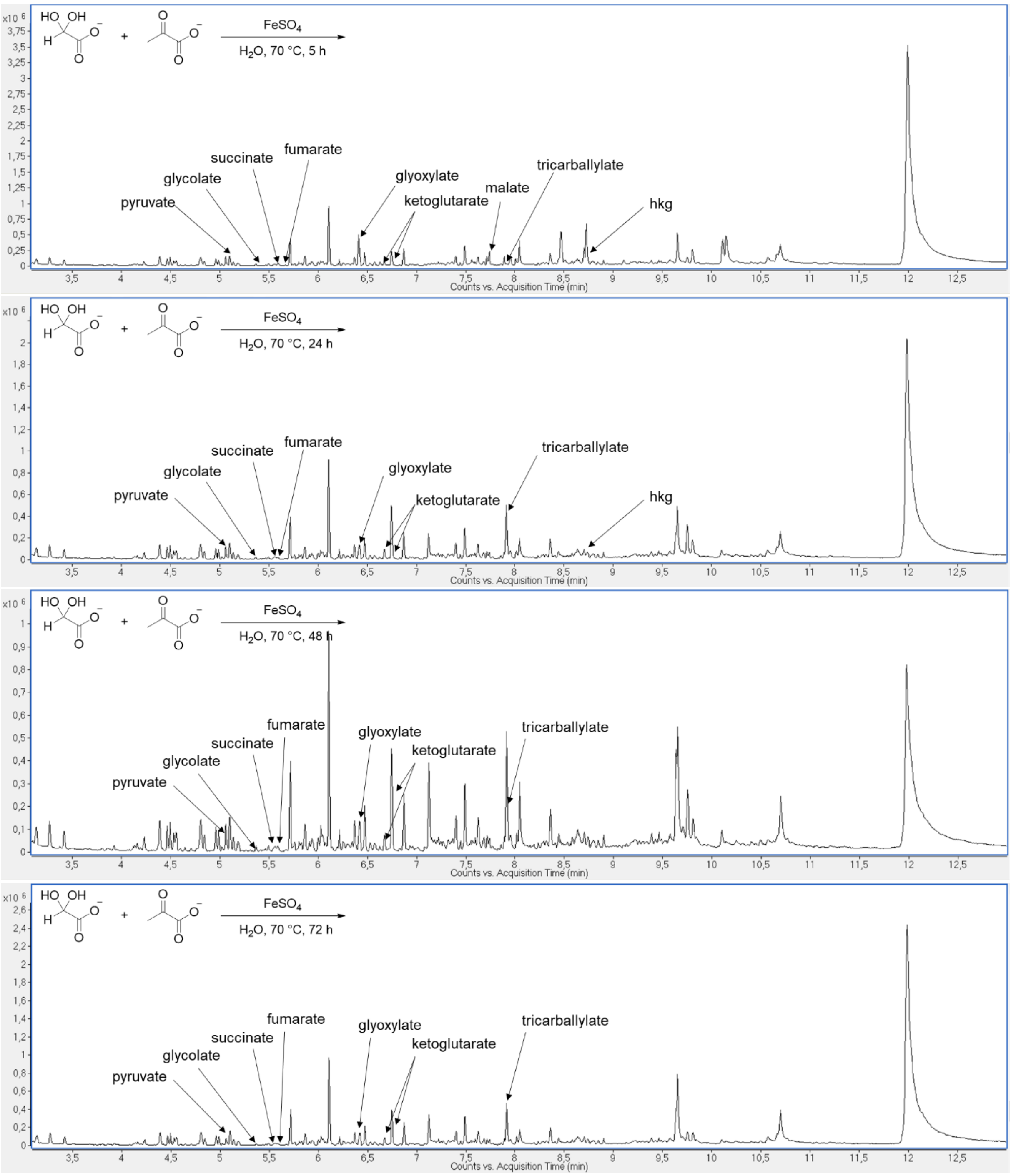

Figure S38. Gas chromatograms of derivatized samples taken from the reaction mixture containing $FeSO_4$ at different points in time.

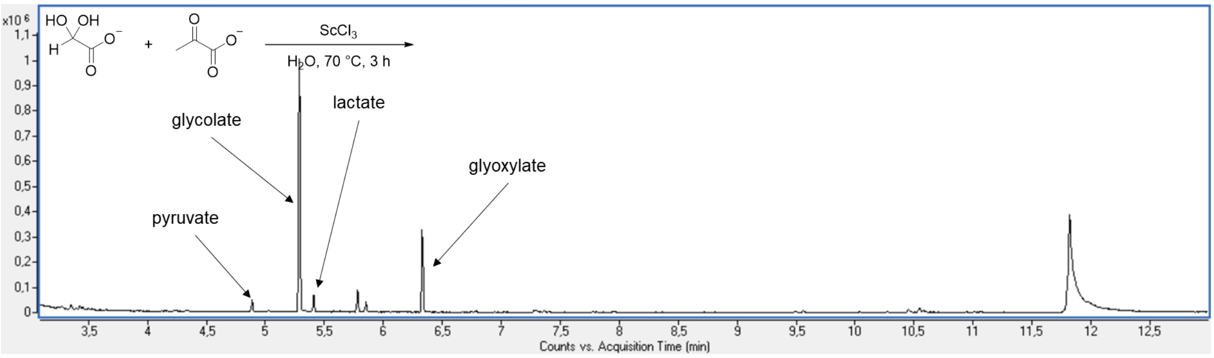

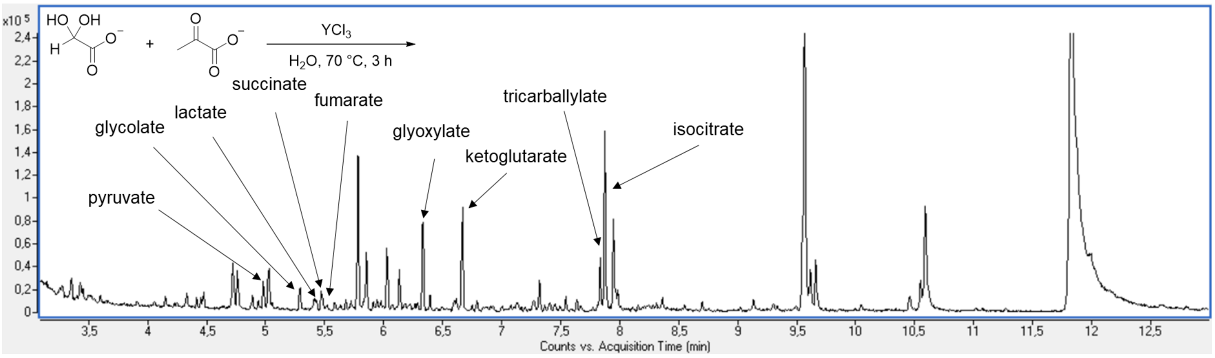
x10 5
YCl_3
H_2O, 70 °C, 3 h
pyruvate
glycolate
lactate
succinate
fumarate
glyoxylate
ketoglutarate
tricarballylate
isocitrate
Counts vs. Acquisition Time (min)

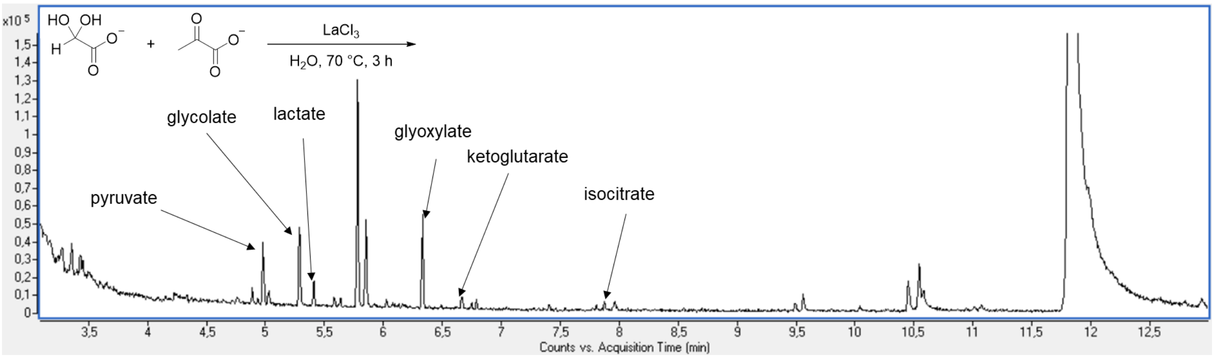
x10 5
$LaCl_3$
H_2O, 70 °C, 3 h
pyruvate
glycolate
lactate
glyoxylate
ketoglutarate
isocitrate
Counts vs. Acquisition Time (min)

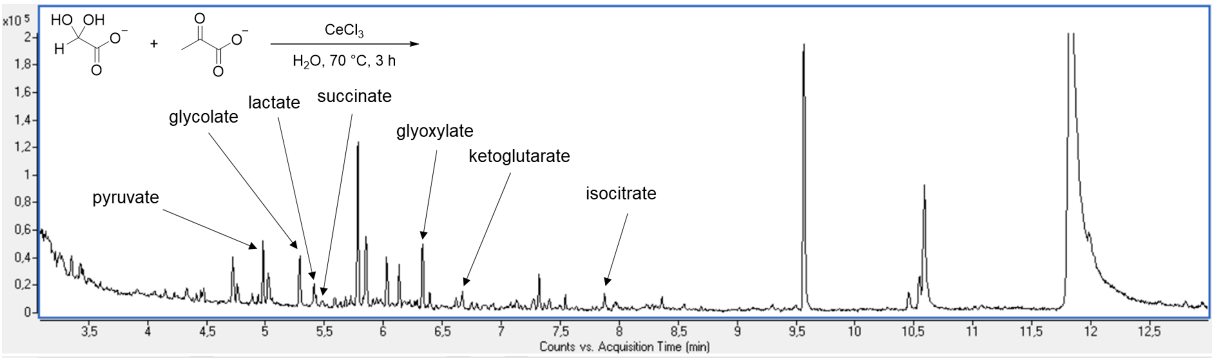
x10 5
$CeCl_3$
H_2O, 70 °C, 3 h
pyruvate
glycolate
lactate
succinate
glyoxylate
ketoglutarate
isocitrate
Counts vs. Acquisition Time (min)

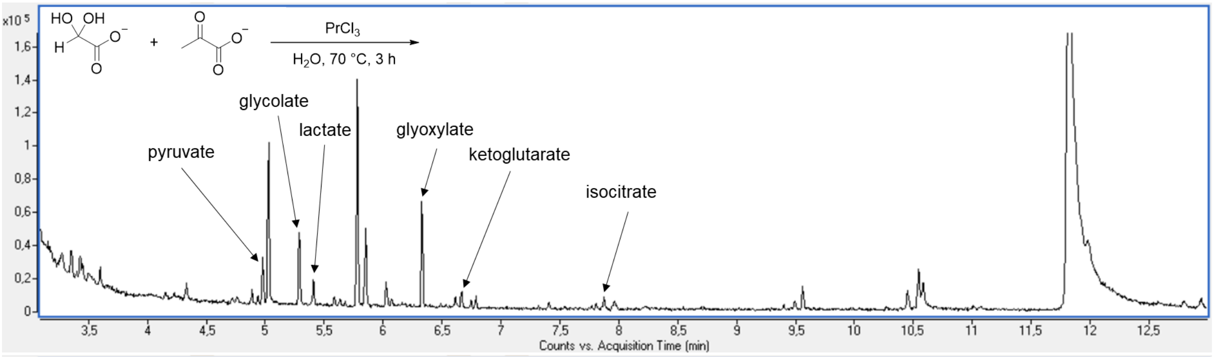
x10 5
$PrCl_3$
H_2O, 70 °C, 3 h
pyruvate
glycolate
lactate
glyoxylate
ketoglutarate
isocitrate
Counts vs. Acquisition Time (min)

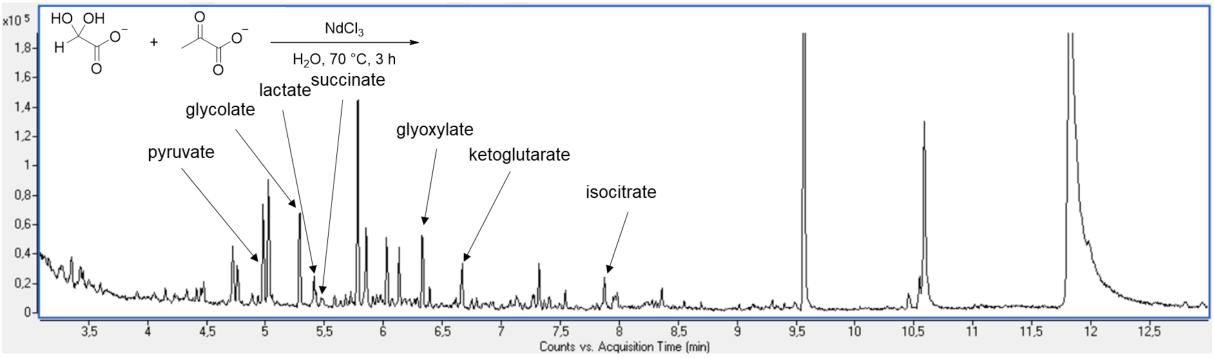
x10 5
$NdCl_3$
H_2O, 70 °C, 3 h
pyruvate
glycolate
lactate
succinate
glyoxylate
ketoglutarate
isocitrate
Counts vs. Acquisition Time (min)

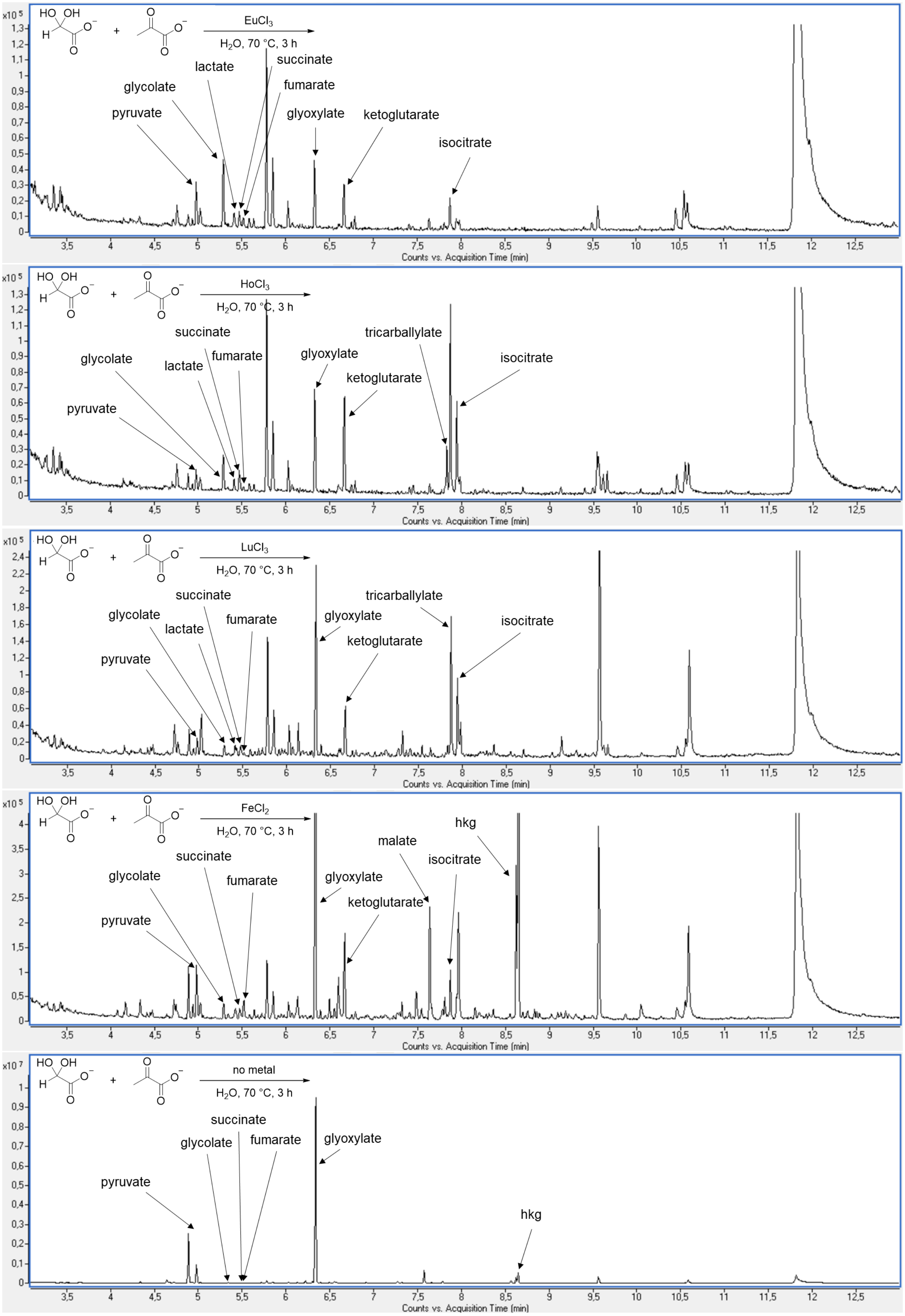

Figure S39. Gas chromatograms of derivatized samples taken from the reaction mixture containing different REE chlorides, iron(II) chloride and without any metal after 3 h.

Rare-earth Chlorides *vs.* $FeCl_2$

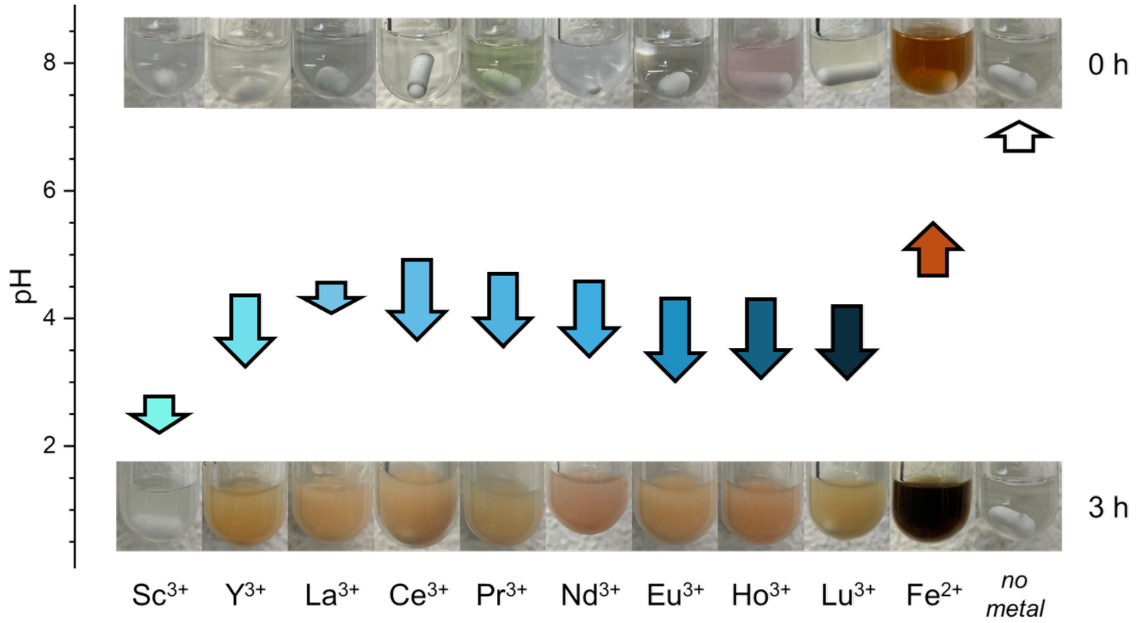

Figure S40. Comparison of pH change of the reactions containing the various metal ions and no metal ion for comparison. The arrow for each ion starts at the pH value after metal addition (0 h) and ends at the value after 3 h at 70 °C.

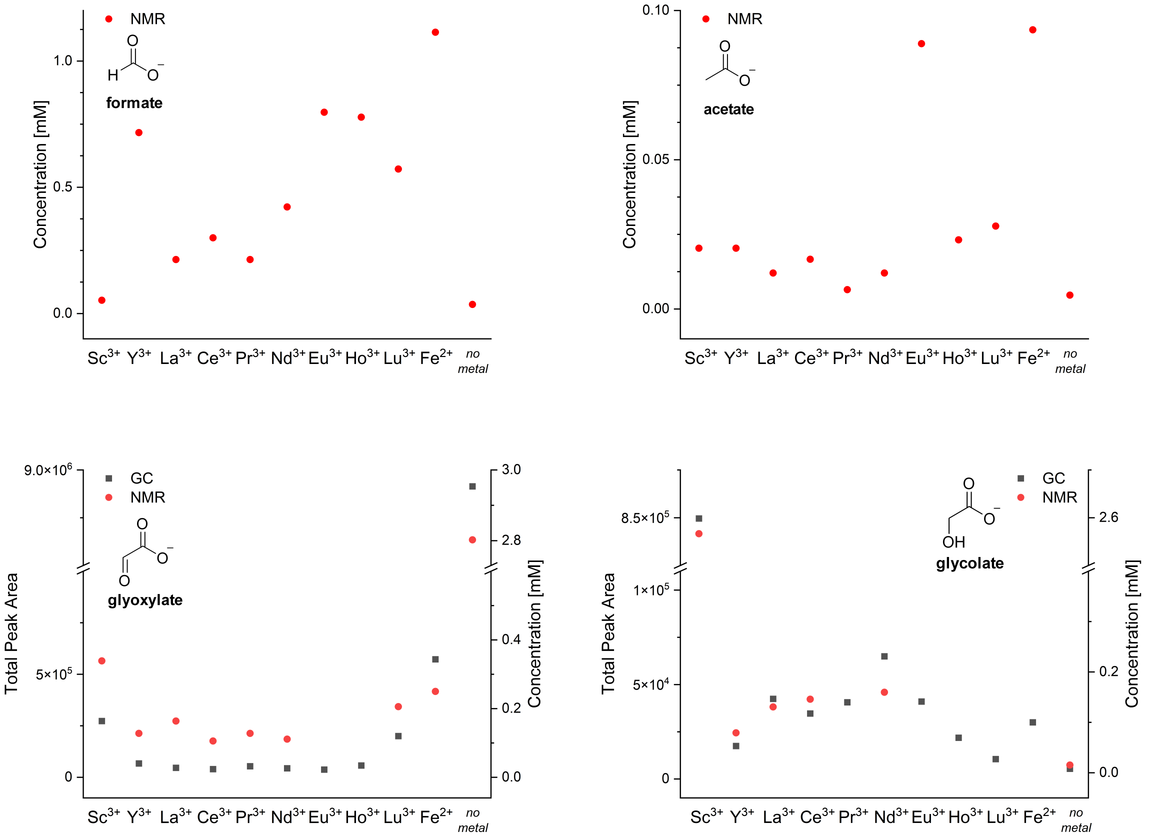

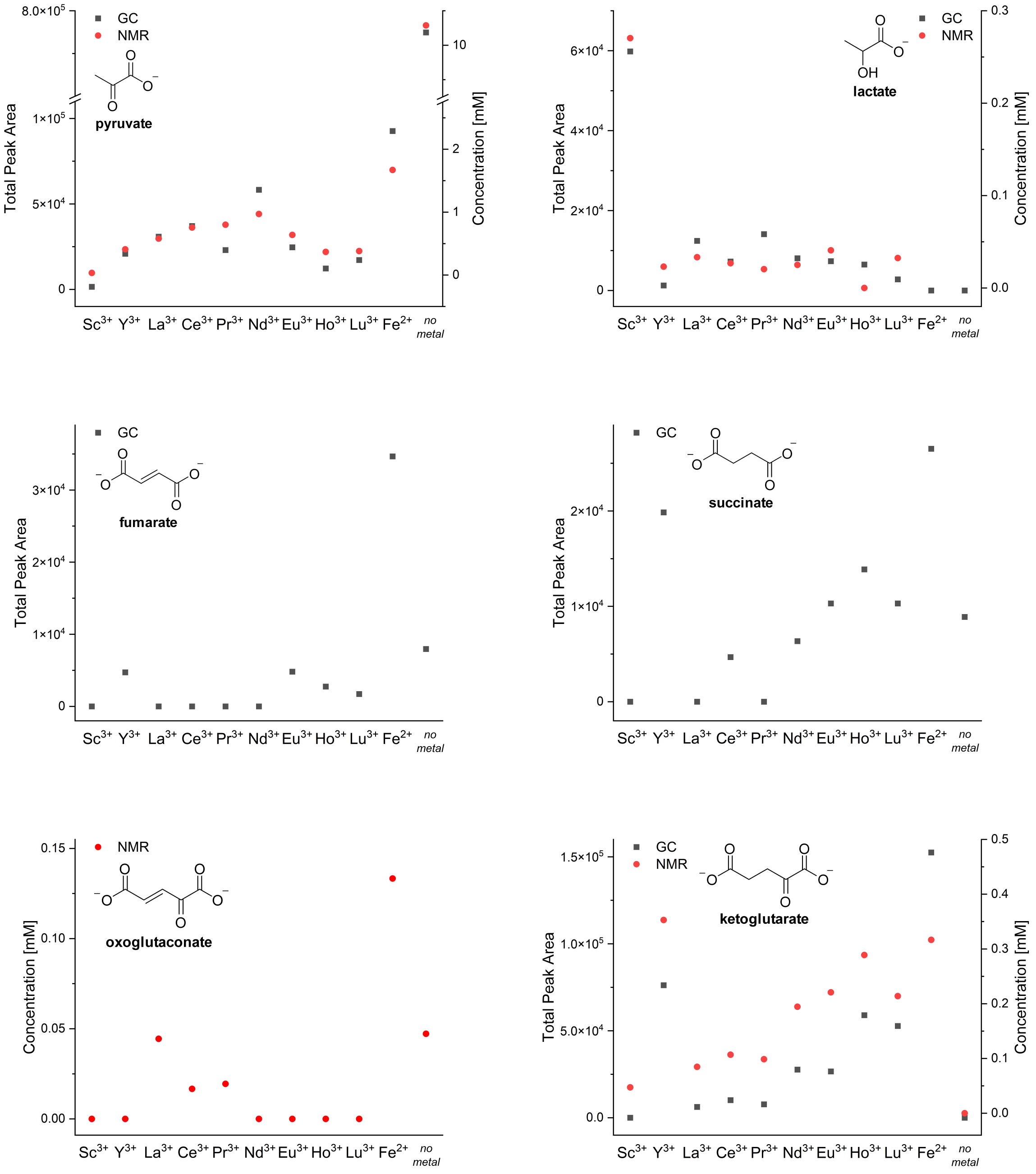
GC
NMR
pyruvate
lactate
fumarate
succinate
oxoglutaconate
ketoglutarate
Total Peak Area
Concentration [mM]
Sc3+ Y3+ La3+ Ce3+ Pr3+ Nd3+ Eu3+ Ho3+ Lu3+ Fe2+ no metal

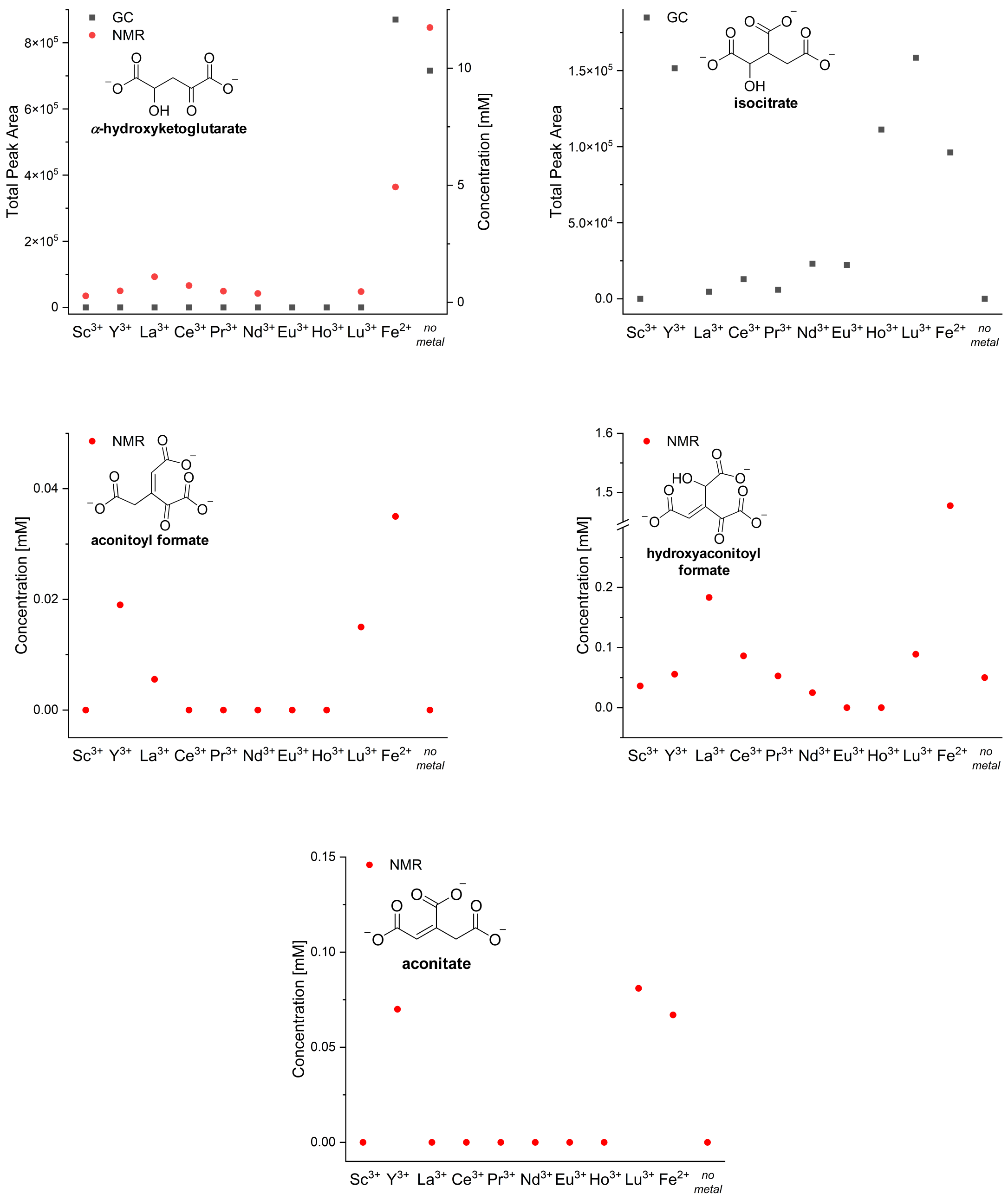

Figure S41. Total peak areas of the assignable products according to GC-MS and concentrations (mM) according to NMR of the reactions containing the various metal ions and no added metal ion for comparison after 3 h at 70 °C. The products which could be detected with both methods are plotted together in one diagram. In the cases of glyoxylate and glycolate, the respective resonance in the ^{1}H NMR is close to the suppressed water resonance and could not be integrated reliably for all reactions. In the case of *α*-hydroxyketoglutarate, spectral broadening as a result of trace amounts of paramagnetic ions present in solution due to insufficient removal makes reliable integration impossible for the reactions containing Eu^{3+} and Ho^{3+}.

IR Spectra

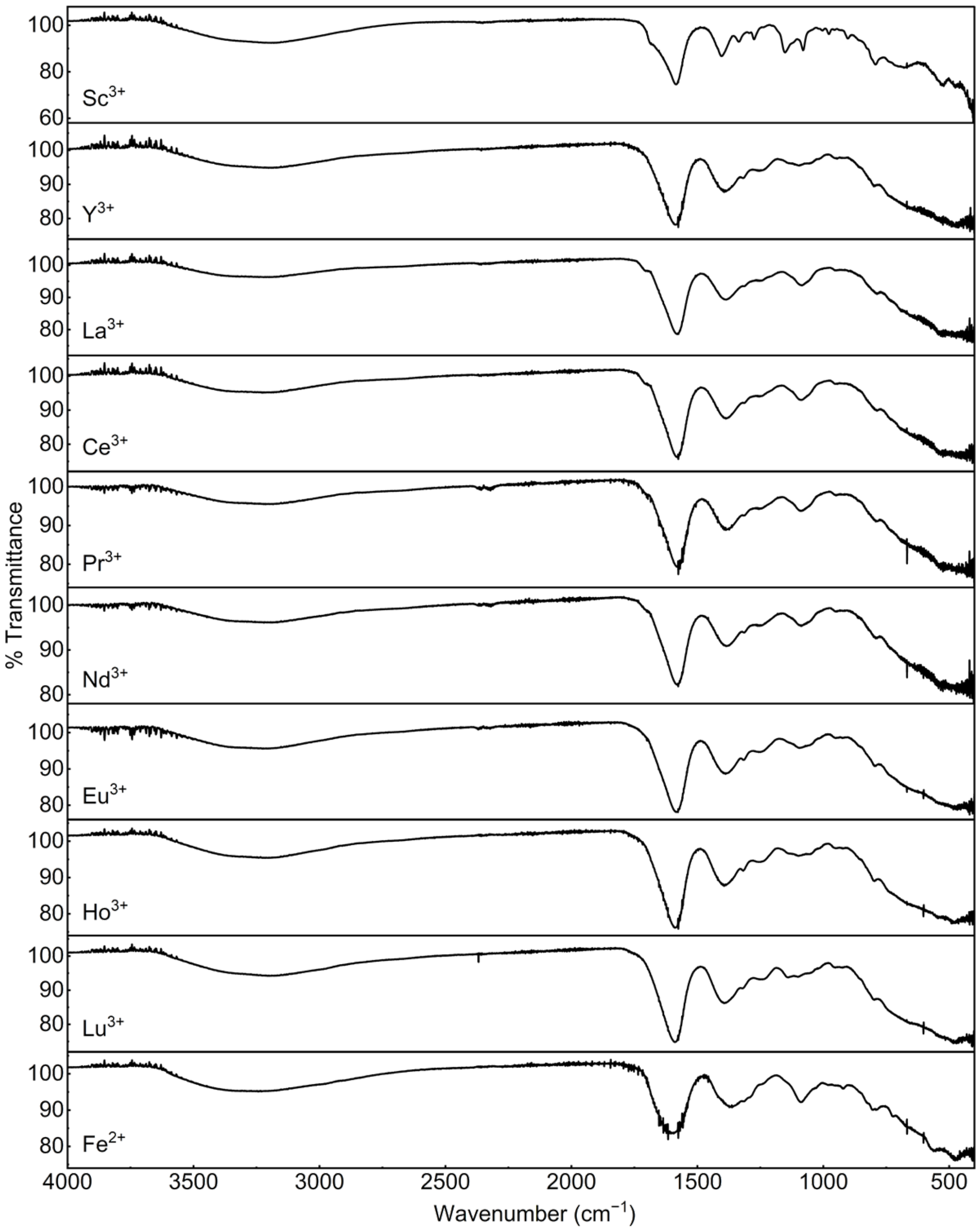

Figure S42. IR spectra of the precipitates collected after 3 h of the reactions containing the REE chlorides and $FeCl_2$.

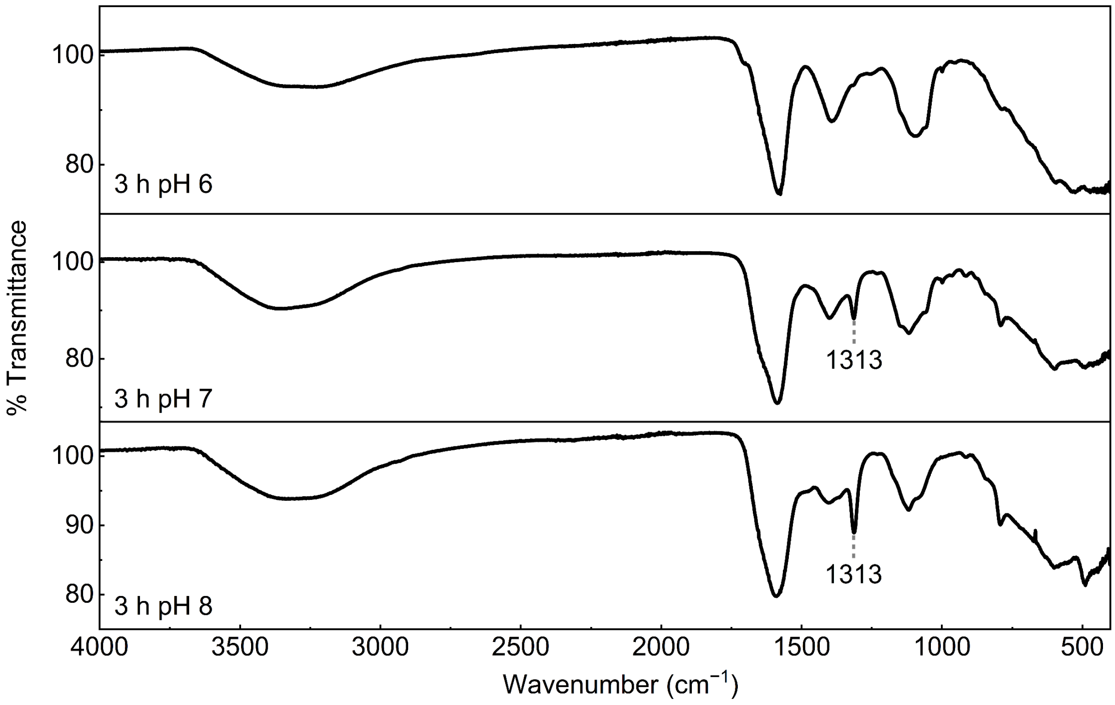

Figure S43. IR spectra of the precipitate collected after 3 h of reactions in the presence of $La_2(SO_4)_3$ with different starting pH values.

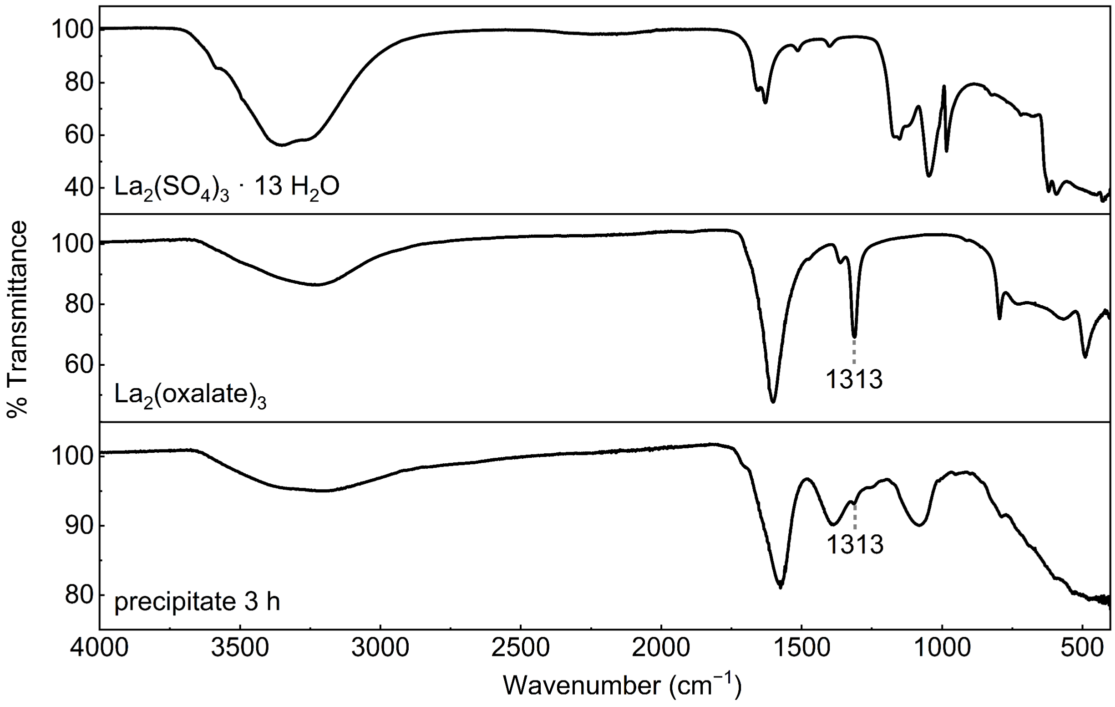

Figure S44. IR spectrum of the precipitate collected after 3 h of reaction in the presence of $La_2(SO_4)_3$ with spectra of $La_2(SO_4)_3{\cdot}H_2O$ and lanthanum(III) oxalate for comparison.

Comparison of the La^{3+}-mediated Reaction Network with the biological Krebs Cycle

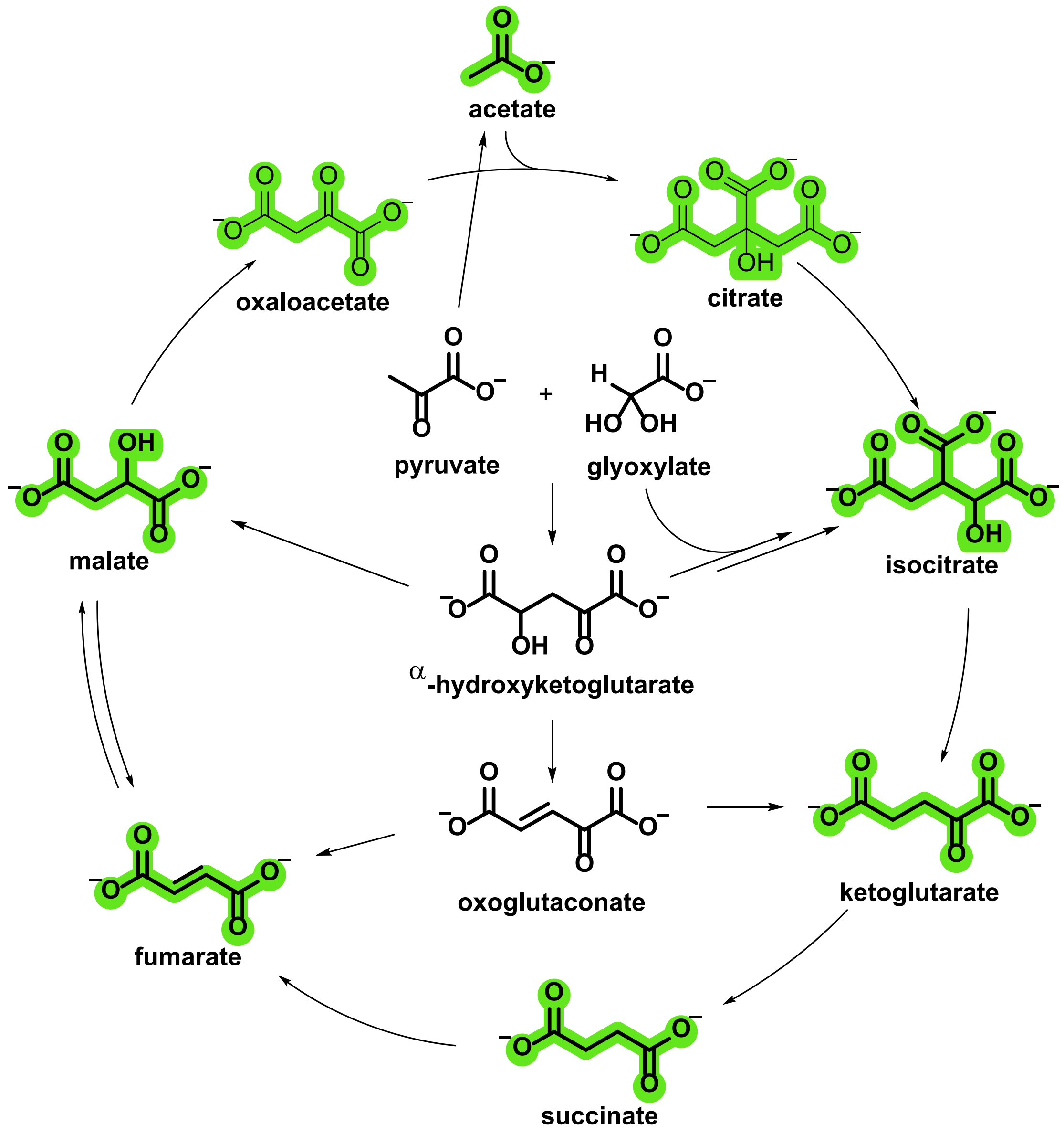

Scheme S1. Comparison of the intermediates of the biological Krebs cycle (highlighted in green) with the intermediates formed in the reaction network involving La^{3+} (depicted in bold).

Possible mechanism of pyruvate reduction:

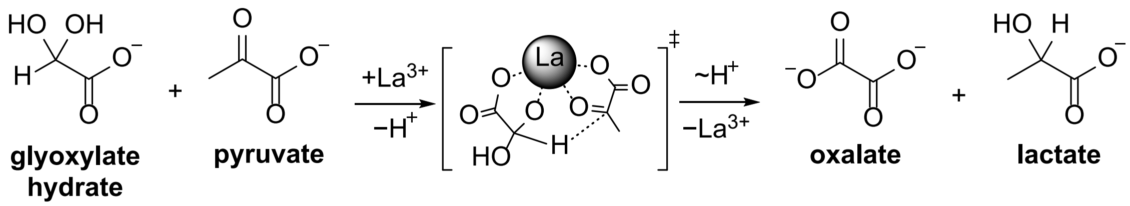

Scheme S2. Possible mechanism of a La^{3+}-mediated crossed Cannizzaro reaction of glyoxylate hydrate with pyruvate. Further coordination around the metal center is omitted due to the lack of supporting data.

References

[1] K. B. Muchowska, S. J. Varma, J. Moran, *Nature* **2019**, *569*, 104–107.
[2] Y.-K. Su, G. N. Short, S. A. Miller, *Green Chem.* **2023**, *25*, 6200–6206.
[3] V. Hélaine, J. Rossi, T. Gefflaut, S. Alaux, J. Bolte, *Adv. Synth. Catal.* **2001**, *343*, 692–697.
[4] Y.-K. Su, G. N. Short, S. A. Miller, *Green Chem.* **2023**, *25*, 6200–6206.
